# Comparative Effectiveness of Digital Exercise-Based Interventions for Improving Balance in Older Adults: Systematic Review and Network Meta-Analysis With GRADE Certainty of Evidence

**DOI:** 10.2196/101231

**Published:** 2026-10-28

**Authors:** Fangbo Li, Xuecheng Li, Qingfang Li, Xiaoqin Gong, Kexin Shi, Yongyu Huang, Xiaowei Feng, Dong Zhang

**Affiliations:** 1 School of Physical Education and Sports Science, South China Normal University, Guangzhou, Guangdong, China; 2 Guangzhou Sport University, Guangzhou, Guangdong, China; 3 School of Football, Hainan Normal University, Haikou, Hainan, China; 4 College of Physical Education, Quanzhou Normal University, Main Campus: No. 398, Donghai Street, Fengze District, Quanzhou, Fujian, 362000, China, 86 18669866186

**Keywords:** network meta-analysis, technology-based rehabilitation, older adults, balance, accidental falls, telerehabilitation, Grading of Recommendations Assessment, Development, and Evaluation, GRADE approach

## Abstract

**Background:**

Technology-based rehabilitation is increasingly used for balance training in older adults. A 2023 network meta-analysis (NMA) found only exergaming with motion capture superior to no treatment; however, the evidence has since expanded, and no GRADE (Grading of Recommendations Assessment, Development, and Evaluation) appraisal has been applied.

**Objective:**

This study aimed to update the comparative effectiveness of 5 technology categories—exergaming with balance platforms (EBP), exergaming with motion capture (EMC), other serious games, interventions with wearables, and telerehabilitation (Tele)—versus conventional exercise (CE), and no treatment (NT), applying GRADE adapted for NMA.

**Methods:**

A systematic search of 4 databases from inception through April 2025, with an identical supplementary update search to January 31, 2026, identified 60 randomized controlled trials (N=3469; mean age 73.1, SD 5.2; range 63.3-86.2 y). Fifty-seven trials (2758 analyzed participants) contributed to 4 prespecified networks (all balance, functional scales, Timed Up and Go [TUG] test, and gait speed). A random-effects NMA was fitted using multivariate meta-regression. Treatment hierarchies were summarized by the surface under the cumulative ranking curve (SUCRA) and the probability of being best; certainty of evidence was rated with GRADE adapted for NMA.

**Results:**

In the all-balance network, Tele (standardized mean difference [SMD] 0.69, 95% CI 0.08‐1.29; *P*=.03), EBP (SMD 0.63, 95% CI 0.31‐0.95; *P*<.001), EMC (SMD 0.58, 95% CI 0.22‐0.95; *P*=.002), and CE (SMD 0.35, 95% CI 0.03‐0.68; *P*=.03) were superior to NT, with consistent findings in the TUG network (EBP 0.81, 95% CI 0.42‐1.20; Tele 0.82, 95% CI 0.16‐1.49; EMC 0.79, 95% CI 0.36‐1.21; all *P*<.05). Tele ranked first by SUCRA (76.3%), but its probability of being best was only approximately 41%, and the estimate rested on 5 trials (n=198). No technology-based intervention was superior to CE in any network. Certainty of evidence was moderate for EBP versus NT and EMC versus NT in the TUG network only; the 37 other comparisons were low or very low. Dropout did not differ between technology-based and control arms (odds ratio 1.10, 95% CI 0.87‐1.40; *P*=.41).

**Conclusions:**

Tele, EBP, and EMC were each associated with improved balance versus NT, although none exceeded CE. The first-place SUCRA ranking of Tele is preliminary. Certainty was moderate only for EBP and EMC versus NT on the TUG test; the remaining evidence is too uncertain for firm clinical recommendations. As no included trial reported falls, these findings are not direct evidence of fall prevention. Larger trials with standardized comparator intensity and posttraining follow-up are required.

## Introduction

### Background

Population aging is a major global health challenge. The global population aged ≥60 years is projected to surpass 2.2 billion by the late 2070s [1]. Falls are among the most common threats to health and independence in later life. The World Health Organization estimates that between 28% and 35% of adults aged ≥65 years experience at least one fall per year, a rate that rises to 32% to 42% among those aged >70 years [2]. The Global Burden of Disease Study 2021 documents approximately 45.7 million new falls among older adults annually, with an estimated 37.3 million requiring medical attention [3]. Falls also increase the risk of mortality and reduce quality of life through fear of falling, functional decline, and institutionalization [4].

Exercise is the best-supported intervention for fall prevention in older adults. The 2022 World Guidelines for Falls Prevention and Management assign a strong recommendation to multicomponent exercise programs targeting balance, strength, and functional mobility [4]. However, adherence to conventional exercise (CE) programs is often poor, and motivation, access, and travel to clinical settings are common barriers [5]. Technology-based approaches have attracted interest because they may improve engagement and reach people who cannot attend in person.

The rationale for technology-based rehabilitation derives largely from augmented sensory feedback. Augmented feedback (ie, extrinsic information about movement outcomes) supports motor learning and postural adaptation [6]. In balance training, real-time feedback on postural sway promotes lasting changes in motor control by facilitating corrective movement strategies [7]. Digital technologies deliver this feedback through several distinct modalities.

Exergaming with balance platforms (EBP) provides real-time feedback on center-of-pressure displacement via devices such as the Nintendo Wii Balance Board. Exergaming with motion capture (EMC) captures whole-body kinematics to generate on-screen visual feedback, as implemented via the Microsoft Kinect. Other serious games (OSG) are purpose-built rehabilitation software increasingly delivered through head-mounted displays. Interventions with wearables (IW) provide haptic or auditory postural feedback via body-worn sensors. Telerehabilitation (Tele) encompasses remotely supervised exercise programs delivered via digital communication platforms, with uptake accelerating substantially after the COVID-19 pandemic [8].

### Prior Work and Rationale for an Update

Most previous systematic reviews and meta-analyses have examined each technology category separately, so their relative effectiveness and their place within fall prevention programs remain unclear [9,10]. Network meta-analysis (NMA) combines direct and indirect evidence in a single model and can rank treatments even where head-to-head comparisons are absent from the primary literature [11]. Cieślik et al [12] published the first NMA in this field in 2023 and found that EMC was the only technology significantly superior to no treatment (Timed Up and Go [TUG]: standardized mean difference [SMD] −0.70, 95% CI −1.16 to −0.23), while Tele, serious games, and wearable interventions lacked sufficient evidence for definitive conclusions.

The evidence base has expanded substantially since that review’s search cutoff in June 2023. Newer randomized controlled trials (RCTs) have tested Tele protocols in larger samples and over longer periods than the trials available to the original review [12], head-mounted virtual reality systems have been studied more widely as platforms for serious games, and wearable sensors now support home-based programs [13,14]. The original NMA also did not apply GRADE (Grading of Recommendations Assessment, Development, and Evaluation), so no formal certainty rating exists for any pairwise comparison [12]. An updated synthesis is therefore warranted to incorporate the newer evidence and to formally assess the certainty of evidence.

### Objectives

We aimed to (1) quantify the comparative effectiveness of 5 categories of technology-based rehabilitation intervention (EBP, EMC, OSG, IW, and Tele) relative to CE and no treatment (NT) across 4 prespecified outcome networks (all balance outcomes, functional scales, TUG, and gait speed); (2) establish a probabilistic treatment hierarchy using the surface under the cumulative ranking curve (SUCRA) and the probability of being the best treatment; and (3) appraise the certainty of evidence for each comparison using the GRADE framework adapted for NMA [15].

## Methods

### Study Design and Registration

This study was designed as a systematic review and NMA, conducted in accordance with the PRISMA (Preferred Reporting Items for Systematic Reviews and Meta-Analyses) 2020 statement and its NMA extension (PRISMA-NMA) [16,17]. The completed PRISMA 2020 and PRISMA-NMA checklists are provided in Checklists 1 and 2, respectively. The protocol was registered with PROSPERO (International Prospective Register of Systematic Reviews; CRD420261380567) and constitutes an updated and expanded synthesis of the original review by Cieślik et al [12].

As the PROSPERO record was completed after the primary searches had been executed, this review is reported as a retrospectively registered update rather than as a prospectively registered de novo review. No eligibility criterion, outcome definition, or analytical decision was altered after the authors had inspected the effect estimates. Two departures from the registered protocol occurred and are reported here in full. First, the protocol specified the *netmeta* R package with P-score ranking, whereas the primary analysis was implemented in Stata using SUCRA; SUCRA and the netmeta P-score are numerically near-equivalent frequentist ranking metrics, and the treatment hierarchy was unaffected by this change. Second, the meta-regressions of training dose and of CE intensity, the treatment-ranking probabilities, and the quantitative transitivity summary were added in response to peer review; these are reported throughout as post hoc exploratory analyses and are interpreted accordingly.

### Literature Search

A systematic search was conducted across 4 electronic databases (PubMed, Embase, Cochrane Central Register of Controlled Trials, and Web of Science) from inception through April 2025. Database-specific search strategies combined controlled vocabulary terms with free-text synonyms covering older adult populations (aged ≥60 y), technology-based interventions (virtual reality, exergaming, telerehabilitation, and wearables), and balance and functional mobility outcomes. A randomized-design filter was applied in PubMed, Embase, and Web of Science; the Cochrane Central Register of Controlled Trials contains only trial records and required none. Each strategy also carried an exclusion block to remove records indexed with the neurological, neurodegenerative, orthopedic, oncological, and other comorbid conditions listed under eligibility, as well as reviews, meta-analyses, and study protocols. The complete search strings for each database, with the date each was executed, are provided in Table S1 in Multimedia Appendix 1.

As peer review and revision extended into 2026, an identical supplementary update search was executed across all 4 databases on January 31, 2026, to capture records that were published, or made available online ahead of print, after the primary search date. Screening and data extraction for the supplementary search followed the same procedures as for the primary search. The record counts, screening numbers, and PRISMA flow diagram reported in the “Results” section represent the combined yield of the primary and supplementary searches. The supplementary search contributed 4 additional eligible trials [18-21], all of which were incorporated into the quantitative synthesis; no study identified after the primary search was used for contextual discussion only. One of these trials appears with a 2026 citation year because it was assigned to a 2026 print issue after online publication [18]; it was retrieved by the supplementary search.

### Eligibility Criteria and Study Selection

Studies were eligible if they (1) enrolled community-dwelling or facility-dwelling adults aged ≥60 years without neurodegenerative, neurological, major orthopedic, or oncological comorbidities; (2) examined an eligible technology-based rehabilitation intervention with at least one control group; (3) reported at least one prespecified primary outcome; and (4) adopted a randomized design. Studies were excluded if the intervention involved robotic exoskeletons or if data were reported only as conference abstracts without peer review. Two independent reviewers (FL and XL) screened all records, and disagreements were resolved through discussion with a third reviewer (DZ). The number of full-text articles excluded at the eligibility stage, broken down by reason for exclusion, is provided in Table S2 in Multimedia Appendix 1.

### Intervention Classification

Eligible technology-based interventions were classified into 5 mutually exclusive categories following the taxonomy of Cieślik et al [12]: (1) EBP, real-time center-of-pressure feedback devices (eg, Wii Balance Board); (2) EMC, systems capturing full-body kinematics for on-screen visual feedback (eg, Microsoft Kinect); (3) OSG, rehabilitation software not relying on balance platforms or motion capture; (4) IW, exercise programs using wearable sensor feedback; and (5) Tele, remotely supervised exercise delivered via digital communication platforms. Two control categories were designated: CE and NT.

Classification was performed independently by 2 reviewers for every arm of every included trial, with disagreements resolved by discussion and, where necessary, by a third reviewer. Where an intervention displayed features of more than 1 category, assignment followed the dominant source of augmented feedback: interventions delivering feedback derived primarily from center-of-pressure displacement were assigned to EBP even when delivered remotely, whereas interventions whose defining feature was synchronous remote supervision were assigned to Tele. Arms combining a technology with CE in a factorial manner (2 arms in 2 trials) could not be assigned to a single node and were excluded from the networks, while the remaining arms of those trials were retained. The as-extracted description and resulting node assignment for every arm of every included trial are reported in Table S3 in Multimedia Appendix 1, so that each classification decision can be checked.

### Outcome Measures and Network Structure

Outcomes were prespecified into 4 analytical networks: (1) network 1 (all balance), aggregating results from all eligible balance-related instruments, including the TUG, Berg Balance Scale (BBS), Mini-Balance Evaluation Systems Test (Mini-BESTest), Short Physical Performance Battery (SPPB), Fullerton Advanced Balance Scale (FAB), Tinetti Performance-Oriented Mobility Assessment (POMA), Functional Reach Test, single-leg stance, center-of-pressure (COP) parameters, and sit-to-stand tests; (2) network 2 (functional scales), restricted to multidimensional clinical scales (BBS, Mini-BESTest, SPPB, FAB, and Tinetti/POMA); (3) network 3 (TUG), the TUG test and its equivalent, the 8-Foot Up-and-Go test; and (4) network 4 (gait speed), quantitative gait speed from 4- or 10-m walk tests. Where a trial reported more than 1 instrument eligible for network 1, a single instrument per trial was selected using a prespecified hierarchy (TUG, followed by BBS, Mini-BESTest, SPPB, and other instruments), so that no trial contributed more than 1 estimate to any network. The secondary outcome was participant acceptability, defined as the all-cause dropout rate.

Network 1 is a composite of instruments that measure different constructs (dynamic functional mobility, multidimensional clinical rating scales, static postural sway, and lower-limb function). It was retained because it maximizes the evidence contributing to each comparison, but it is reported alongside, and not in preference to, the construct-specific networks; network 3 (TUG) provides the most clinically interpretable single-construct estimates and is the basis for the principal clinical conclusions of this review.

### Data Extraction

Data were extracted by 1 reviewer and independently verified by a second reviewer. For each eligible arm, we recorded the number of participants analyzed for the outcome of interest, together with the number randomized, the postintervention mean, and the postintervention SD. Analyzed rather than randomized denominators were used in the effect-size calculations because postintervention means and SDs are reported for analyzed participants; the 2 denominators differ appreciably in a minority of trials (most notably in the largest included trial [22], in which 503 participants were randomized and 226 contributed postintervention balance data), and both are reported per trial in Table S4 in Multimedia Appendix 1. Postintervention means and SDs were used throughout, as combining posttest and change-score SMDs within a single analysis is not recommended when SMD is the effect measure [23]. Studies reporting only change-from-baseline scores without accompanying postintervention values were therefore excluded from network synthesis. For studies reporting only median and IQR, values were estimated using the formula of Wan et al [24]. Dropout counts were extracted per randomized arm.

We also sought to extract adverse events, technology-related difficulties (eg, device malfunction, connectivity failure, and cybersickness), and participant-reported satisfaction or acceptability to characterize the feasibility and user burden of each modality. These data were too sparsely reported to be synthesized and are summarized descriptively in the “Results” section.

### Risk of Bias Assessment

The methodological quality of included trials was evaluated by 2 independent reviewers using version 2 of the Cochrane Risk of Bias tool (RoB 2) [25], covering 5 domains: randomization (D1), deviations from intended interventions (D2), missing outcome data (D3), outcome measurement (D4), and selective reporting (D5). Overall risk was classified as low risk, some concerns, or high risk. As blinding of participants is structurally impossible in exercise trials, an elevated D2 rating attributable solely to the absence of participant blinding was not by itself treated as a reason to rate down for risk of bias in the GRADE assessment. Instead, following the reasoning that the relevant question is whether the absence of blinding plausibly influenced the result [15], D2 was treated as a source of serious risk of bias only where the trial reports also indicated differential cointerventions, differential attention or contact time, or an intention-to-treat principle that was not followed; D4 was rated based on the presence of blinded outcome assessment; and D5 was rated based on the availability of a prespecified protocol or registration. Domain-level judgments for every trial are provided in Table S5 and Figure S1 in Multimedia Appendix 1.

### Statistical Analysis

The SMD (Hedges *g*) and its SE were calculated for each pairwise arm comparison from postintervention means and pooled postintervention SDs. For outcomes where lower scores indicate better performance (TUG, COP, and sit-to-stand time), the SMD sign was reversed to ensure consistent positive directionality across all outcomes.

All analyses were performed in Stata/SE (version 15.1; StataCorp LLC) using the network suite [26]. Data were structured in the augmented format to accommodate multiarm trials [27], so that the correlation between effect estimates from the same trial was respected and no participant was counted more than once within a comparison; 8 trials contributed 3 arms each to network 1, and the remainder contributed 2. The NMA was implemented as a multivariate meta-analysis via the mvmeta command [28], fitting a random-effects consistency model with restricted maximum likelihood estimation. The between-study SD (τ) was assumed common across comparisons, and between-study covariances were modeled with an exchangeable correlation structure (ρ=0.5) [26,27]. NT was specified as the reference category.

Transitivity was evaluated by comparing the distribution across treatment nodes of mean age, intervention duration, training frequency, total sessions, session length, and study setting (community vs facility dwelling), summarized in Table S6 and Figure S2 in Multimedia Appendix 1. Modifiers reported too inconsistently to evaluate—baseline balance impairment, fall risk or history, frailty, sex distribution, supervision level, comparator intensity, prior technology familiarity, and outcome measurement protocol—are identified as a limitation of the transitivity appraisal. Between-study heterogeneity was quantified by τ and τ². Global statistical inconsistency was assessed using the design-by-treatment interaction model. Local inconsistency for each pairwise contrast was evaluated by node splitting [29-31], decomposing each estimate into its direct and indirect components and testing their agreement.

Treatment hierarchies were estimated by computing SUCRA from 5000 parametric bootstrap draws. SUCRA summarizes a rank distribution and carries no information about the magnitude or the certainty of the underlying effects, so the probability that each intervention was the best treatment was also estimated from the multivariate normal distribution of the network estimates [11] and is reported alongside SUCRA throughout. The post hoc analyses added during peer review—the random-effects meta-regressions of training dose and of CE intensity, the ranking probabilities, and the quantitative transitivity summary—were conducted in Python (version 3.12; Python Software Foundation) using numpy, scipy, and pandas, with figures produced in Matplotlib, and each was cross-checked for numerical agreement against the primary Stata NMA. In the dose meta-regressions, study-level SMDs were regressed on intervention duration, training frequency, total sessions, session length, weekly training minutes, and mean age; the direct CE versus NT effect was separately regressed on CE dose parameters.

Small-study effects were assessed visually using comparison-adjusted funnel plots [32] generated via netfunnel. Formal regression-based testing of funnel plot asymmetry was not undertaken because no comparison in any network was informed by 10 or more trials conventionally regarded as the minimum for such a test to be interpretable; the consequences of this limitation for the assessment of publication bias are considered in the “Discussion” section. As these methods have very low power when fewer than 10 trials contribute to a comparison, they were interpreted as exploratory in networks 2 and 4, and no comparison was rated down for publication bias on the basis of funnel plot appearance alone.

A post hoc sensitivity analysis examined the source of the global inconsistency in network 3 (TUG), where 4 trials provided direct NT versus CE evidence. As any exclusion threshold is arbitrary, 2 specifications were run, and both are reported: specification 1 removed the 2 trials whose direct effect exceeded SMD 2.0, and specification 2 removed the 3 trials exceeding SMD 1.0. All 4 are 3-arm trials, so removal also withdraws technology arms; the effect of each specification on every estimate, every SUCRA value, and the direct-indirect discrepancy is reported in full (Table S7, Multimedia Appendix 1), with ranking stability assessed by the Spearman correlation of SUCRA before and after removal. These analyses characterize the influence of the discrepant contrast; they were not used to select a model, and the primary analysis retains all trials.

GRADE certainty was rated for all pairwise comparisons using the framework adapted for NMA [15], starting at high certainty. The following decision rules were fixed before rating and applied uniformly. Imprecision was rated down 1 level where the 95% CI included the null or a trivial effect (|SMD|<0.20), and 2 levels where it additionally spanned appreciable benefit and harm (|SMD|>0.50) or fewer than 3 trials contributed. In network 1, imprecision was judged against the 95% prediction interval rather than the CI because the heterogeneity of a composite outcome makes the CI understate the uncertainty that applies to any single instrument; all network 1 prediction intervals included the null. Inconsistency was rated down where the design-by-treatment interaction test was significant and node splitting localized the discrepancy to that comparison. Indirectness was not rated down anywhere because all trials were randomized comparisons in the target population using the intended outcomes. Publication bias was rated down only where funnel plot asymmetry coincided with a plausible mechanism and at least 10 contributing trials. Two reviewers rated independently and reached a final rating by consensus.

## Results

### Study Selection and Characteristics

The primary and supplementary searches together yielded 2459 records (PubMed: n=408, 16.6%; Embase: n=775, 31.5%; Web of Science: n=491, 20%; and Cochrane: n=785, 31.9%), of which 1213 (49.3%) were duplicates. After title and abstract screening of 1246 unique records, 188 (15.1%) full-text articles were assessed for eligibility; 128 (68.1%) were excluded (incorrect study type, n=21, 16.4%; incorrect outcomes, n=62, 48.4%; incorrect intervention, n=26, 20.3%; incorrect participants, n=19, 14.8%). Sixty RCTs [18-22,33-87] involving 3469 randomized participants met all inclusion criteria (Figure 1). Four of these trials [18-21] were identified by the supplementary update search.

Fifty-seven (95%) of the 60 included trials (randomized: n=3301; and analyzed arms: n=2758) contributed data to at least one of the 4 networks. Three trials could not contribute quantitative data; they were retained in the systematic review but not included in any network: 1 reported outcomes as change scores only [37]; 1 reported balance solely as a composite *z* score that could not be mapped onto any single network instrument [67]; and 1 randomized participants to 2 arms that were both classified into the same network node, so that it contributed no between-node contrast [56]. All 3 are described in Table S4 in Multimedia Appendix 1.

Ten included trials [18-21,43,68,71,76,78,86] were published, or appeared online, after the June 2023 search cutoff of the original review [12]. Sample sizes ranged from 12 to 503 randomized participants (median 43, IQR 34-64), and the mean age was 73.1 (SD 5.2; range 63.3‐86.2) years (unweighted mean of trial means). EBP (21 arms) and EMC (18 arms) were the most frequently studied categories in the all-balance network (Table 1). Tele trials were much larger on average (mean 124 randomized participants across the 6 Tele trials); although the mean number of participants contributing to each Tele arm in network 1 was 39.6, the largest Tele trial having supplied postintervention data for only a subset of those randomized [22]. Participant characteristics and intervention details for all included studies are summarized in Table S4 in Multimedia Appendix 1.

**Figure 1.**
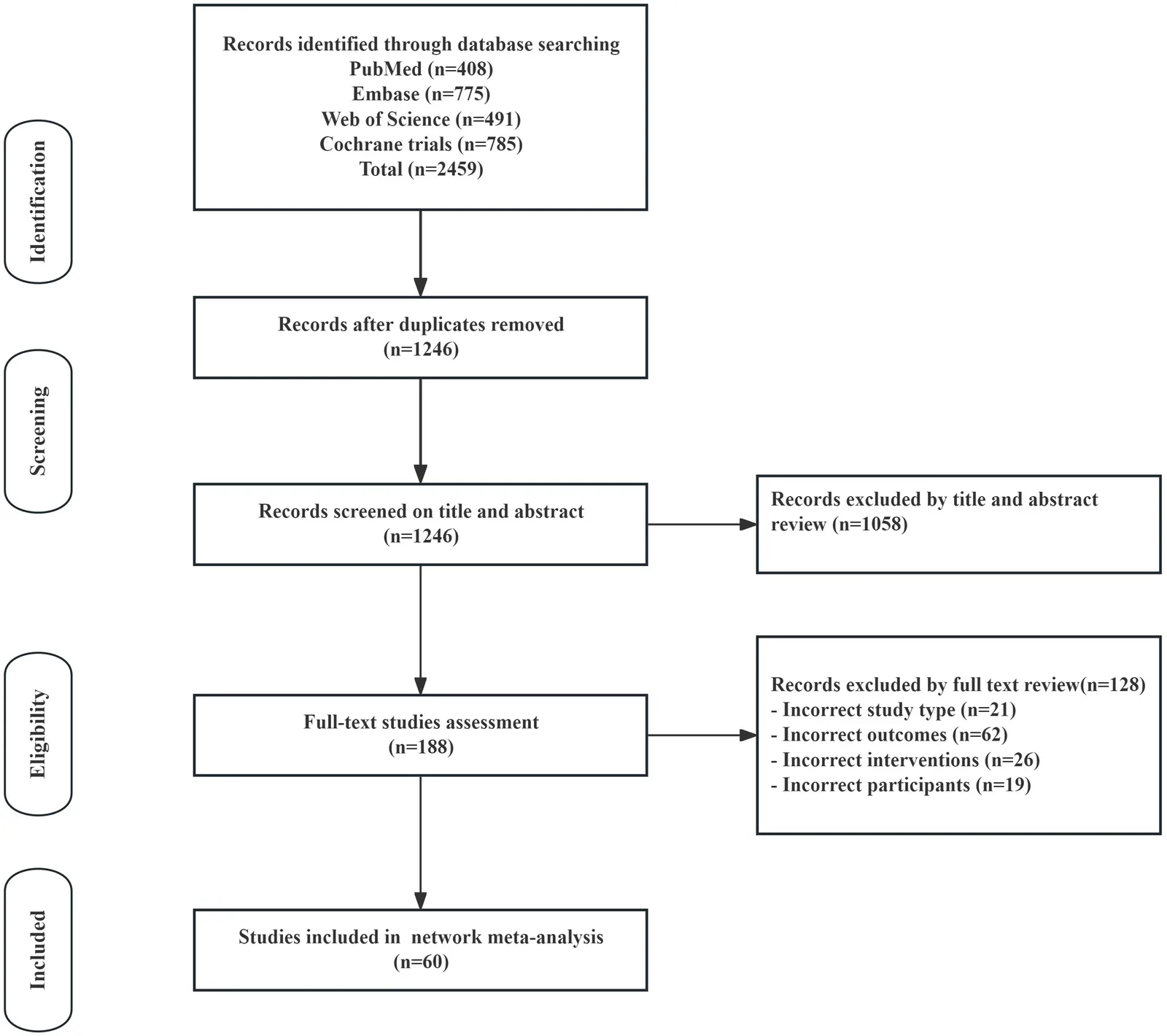
PRISMA (Preferred Reporting Items for Systematic Reviews and Meta-Analyses) flow diagram of study identification, screening, and inclusion, showing the combined yield of the primary search (to April 2025) and the supplementary update search (to January 31, 2026).

**Table 1.** Summary of trial arms and participants by intervention category in the primary (all-balance) network^a^.

Intervention category	Trial arms (N=118), n (%)	Participants (N=2710), n (%)	Participants per arm, mean
Conventional exercise	29 (24.6)	581 (21.4)	20.0
Exergaming, balance platform	21 (17.8)	431 (15.9)	20.5
Exergaming, motion capture	18 (15.3)	360 (13.3)	20.0
Other serious games	10 (8.5)	318 (11.7)	31.8
Telerehabilitation	5 (4.2)	198 (7.3)	39.6
Interventions with wearables	3 (2.5)	29 (1.1)	9.7
No treatment	32 (27.1)	793 (29.3)	24.8

a Full per-study characteristics for all 60 included trials are provided in Table S4 in Multimedia Appendix 1. Values reflect arm-level contributions to network 1 (all balance outcomes). Network 1 included 55 (91.6%) of the 60 included trials; 2 further trials contributed only to the gait-speed network (network 4), and 3 trials contributed no quantitative data to any network. The 118 arms in the column header were contributed by 55 trials separately because 8 trials contributed 3 arms each and the remainder contributed 2.

### Risk of Bias

RoB 2 assessment rated 2 (3%) of 60 studies at low overall risk of bias, 50 (83%) as having raised some concerns, and 8 (13%) at high risk. The predominant source of concern was D2 (deviations from intended interventions; 58/60, 97%, studies rated as having some concerns or high risk), attributable in large part to the structural impossibility of blinding participants to exercise interventions. D4 (outcome measurement) was rated as high risk in 6 (10%) studies, primarily reflecting the absence of blinded outcome assessors. Domain-level judgments for every trial are reported in Table S5 in Multimedia Appendix 1, and the RoB 2 traffic-light plot is presented as Figure S1 in Multimedia Appendix 1.

### Network Structure

Network plots for all 4 analytical networks are presented in Figure 2. Network 1 (all balance) incorporated 55 trials, network 2 (functional scales) incorporated 26 trials, network 3 (TUG) incorporated 41 trials, and network 4 (gait speed) incorporated 12 trials. All 4 networks comprised a single fully connected component. The CE and NT nodes served as the principal hubs across all networks. Direct evidence for comparisons between active treatments was available for only a small proportion of dyads; the number of trials and participants contributing direct evidence to each contrast is tabulated in Table S8 in Multimedia Appendix 1.

**Figure 2.**
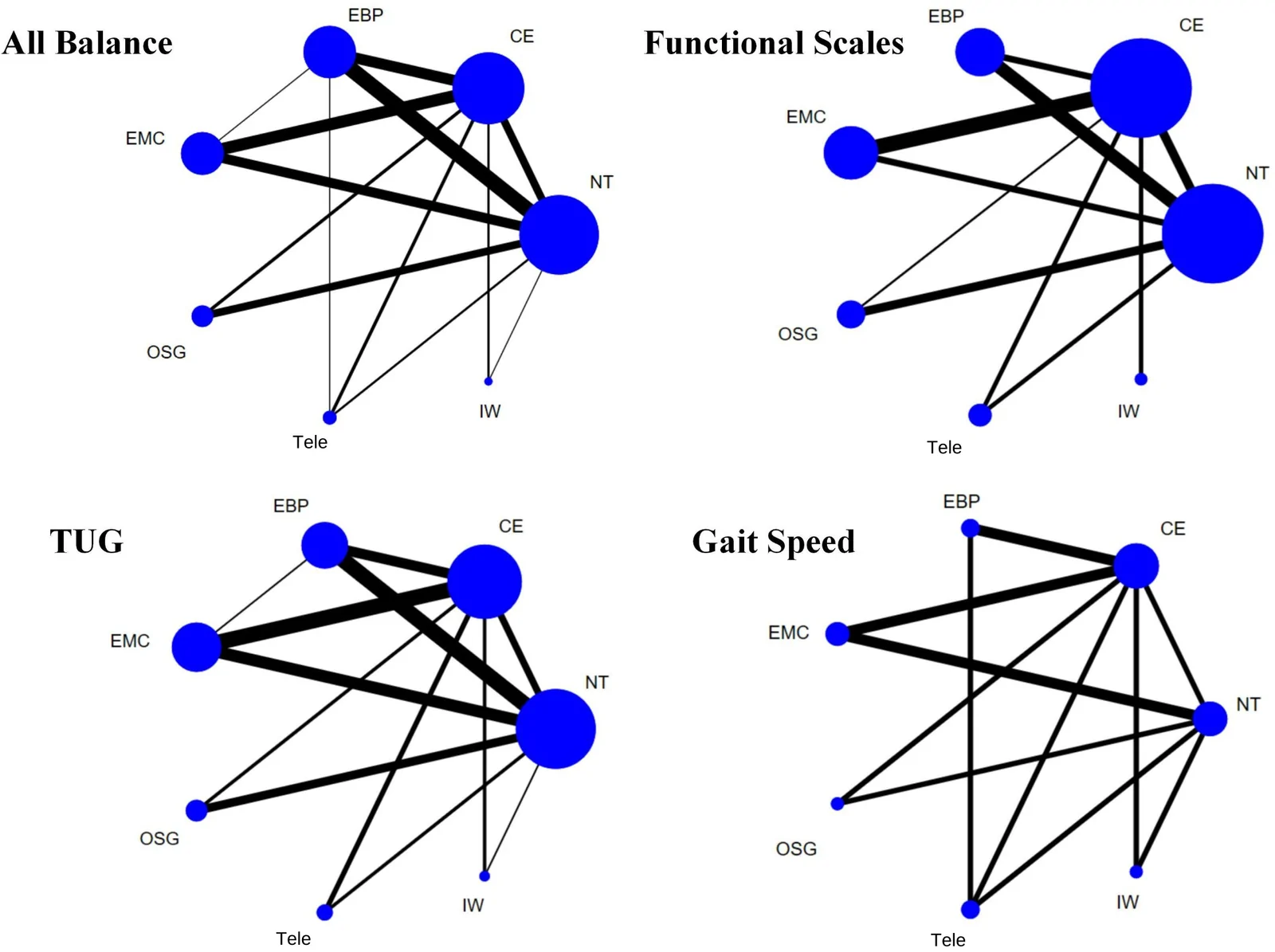
Network plots presenting the direct evidence structure for each of the 4 analytical networks. CE: conventional exercise; EBP: exergaming with balance platforms; EMC: exergaming with motion capture; IW: interventions with wearables; NT: no treatment; OSG: other serious games; Tele: telerehabilitation; TUG: Timed Up and Go.

### NMA Results

Main results for all 4 networks are presented in Table 2. Complete league tables are provided in Figure 3, and all-pairwise forest plots are provided in Figure 4.

**Table 2.** Network meta-analysis results: effect sizes (Hedges *g* SMD^a^), SUCRA^b^ rankings, and GRADE^c^ certainty of evidence for all treatment comparisons, sorted by SUCRA within each network.

Intervention	Studies, n	Participants, n^d^	SMD versus NT^e^ (95% CI)	SMD versus CE^f^ (95% CI)	SUCRA, %	GRADE certainty
Network 1: all balance outcomes (55 studies; global consistency test *χ*²_₁₃_=16.1; *P*=.25)						
Tele^g^	5	198	0.69 (0.08 to 1.29)^h^	0.34 (−0.26 to 0.93)	76.3	⊕⊕○○ Low
EBP^i^	21	431	0.63 (0.31 to 0.95)^j^	0.28 (−0.07 to 0.63)	75.2	⊕⊕○○ Low
EMC^k^	18	360	0.58 (0.22 to 0.95)^j^	0.23 (−0.11 to 0.57)	68.7	⊕⊕○○ Low
OSG^l^	10	318	0.41 (−0.01 to 0.83)	0.06 (−0.41 to 0.52)	47.2	⊕⊕○○ Low
IW^m^	3	29	0.33 (−0.57 to 1.23)	−0.02 (−0.90 to 0.86)	41.2	⊕○○○ Very low
CE	29	581	0.35 (0.03 to 0.68)^h^	—^n^	36.6	⊕⊕○○ Low
Network 2: functional scales (26 studies; global consistency test *χ*²*₉*=13.8; *P*=.13)						
IW	2	14	1.10 (−0.28 to 2.48)	1.00 (−0.25 to 2.25)	88.9	⊕○○○ Very low
OSG	5	163	0.41 (−0.25 to 1.06)	0.31 (−0.48 to 1.10)	62.9	⊕⊕○○ Low
EMC	9	183	0.37 (−0.29 to 1.03)	0.27 (−0.24 to 0.79)	60.9	⊕⊕○○ Low
EBP	7	113	0.18 (−0.44 to 0.80)	0.08 (−0.61 to 0.78)	43.4	⊕⊕○○ Low
Tele	3	175	0.14 (−0.68 to 0.97)	0.05 (−0.79 to 0.88)	39.7	⊕⊕○○ Low
CE	15	286	0.10 (−0.47 to 0.67)	—	32.5	⊕⊕○○ Low
Network 3: Timed Up and Go test (41 studies; global consistency test *χ*²_₁₂_=28.1; *P*=.005; inconsistency localized to NT vs CE)						
EBP	14	307	0.81 (0.42 to 1.20)^j^	0.21 (−0.21 to 0.64)	74.8	⊕⊕⊕○ Moderate
Tele	4	190	0.82 (0.16 to 1.49)^h^	0.23 (−0.41 to 0.87)	72.5	⊕⊕○○ Low
EMC	14	272	0.79 (0.36 to 1.21)^j^	0.19 (−0.19 to 0.57)	71.7	⊕⊕⊕○ Moderate
CE	22	442	0.60 (0.21 to 0.98)^j^	—	47.2	⊕⊕○○ Low^o^
IW	3	29	0.48 (−0.42 to 1.38)	−0.12 (−1.00 to 0.77)	41.3	⊕○○○ Very low
OSG	7	245	0.49 (−0.005 to 0.978)	−0.11 (−0.66 to 0.44)	39.5	⊕⊕○○ Low
Network 4: gait speed (12 studies; supplementary and hypothesis-generating only; all comparisons nonsignificant)						
EMC	3	62	1.51 (−0.68 to 3.70)	0.36 (−1.78 to 2.49)	70.6	⊕○○○ Very low
CE	7	144	1.15 (−0.82 to 3.13)	—	62.0	⊕○○○ Very low
EBP	3	32	0.94 (−1.81 to 3.70)	−0.21 (−2.37 to 1.95)	53.6	⊕○○○ Very low
Tele	3	165	0.91 (−1.53 to 3.35)	−0.24 (−2.49 to 2.00)	52.6	⊕○○○ Very low
IW	2	21	0.85 (−1.83 to 3.52)	−0.31 (−3.00 to 2.39)	50.4	⊕○○○ Very low
OSG	2	99	0.39 (−2.23 to 3.00)	−0.76 (−3.39 to 1.86)	38.8	⊕○○○ Very low

a SMD: standardized mean difference (Hedges *g*; positive values favor the row treatment).

b SUCRA: surface under the cumulative ranking curve.

c GRADE: Grading of Recommendations Assessment, Development, and Evaluation (⊕⊕⊕⊕ high, ⊕⊕⊕○ moderate, ⊕⊕○○ low, ⊕○○○ very low).

d Number of participants in the respective intervention arm across contributing studies, summed at the arm level; the participant counts in Table 3 are the sum of both arms of each pairwise comparison and therefore differ from those reported here.

e NT: no treatment.

f CE: conventional exercise.

g Tele: telerehabilitation.

h *P*<.05 vs NT.

i EBP: exergaming with balance platforms.

j *P*<.01 vs NT. Exact *P* values for every comparison are reported in Table S9 in Multimedia Appendix 1.

k EMC: exergaming with motion capture.

l OSG: other serious games.

m IW: interventions with wearables.

n Not applicable.

o Certainty additionally rated down 1 level for inconsistency (significant direct-indirect discrepancy on node splitting, localized to the NT vs CE comparison).

**Figure 3.**
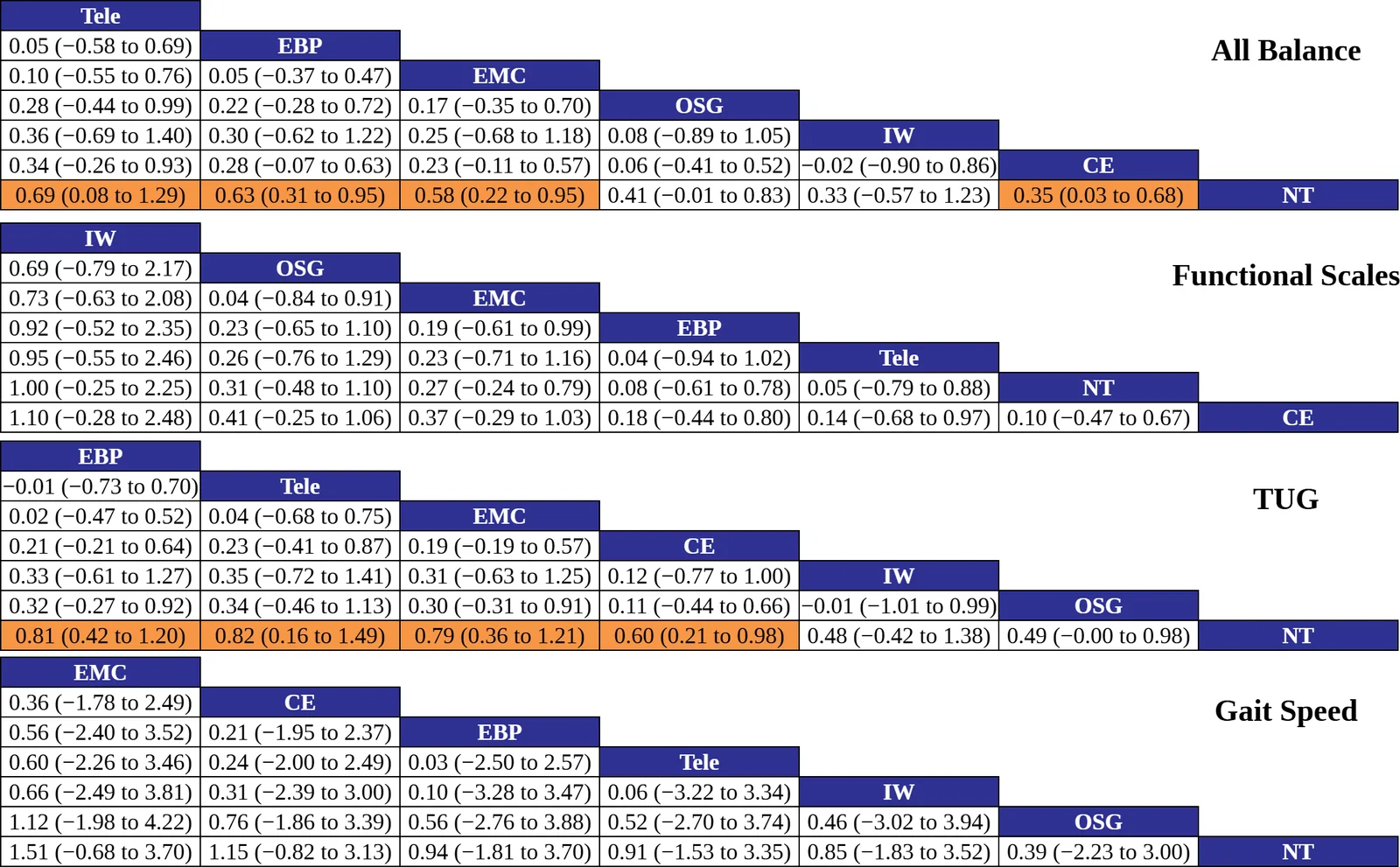
League tables presenting all pairwise network meta-analysis effect estimates (standardized mean difference, 95% CI) for the 4 analytical networks. In the league tables, orange cells indicate statistically significant treatment comparisons, and white cells indicate nonsignificant comparisons. CE: conventional exercise; EBP: exergaming with balance platforms; EMC: exergaming with motion capture; IW: interventions with wearables; NT: no treatment; OSG: other serious games; Tele: telerehabilitation; TUG: Timed Up and Go.

**Figure 4.**
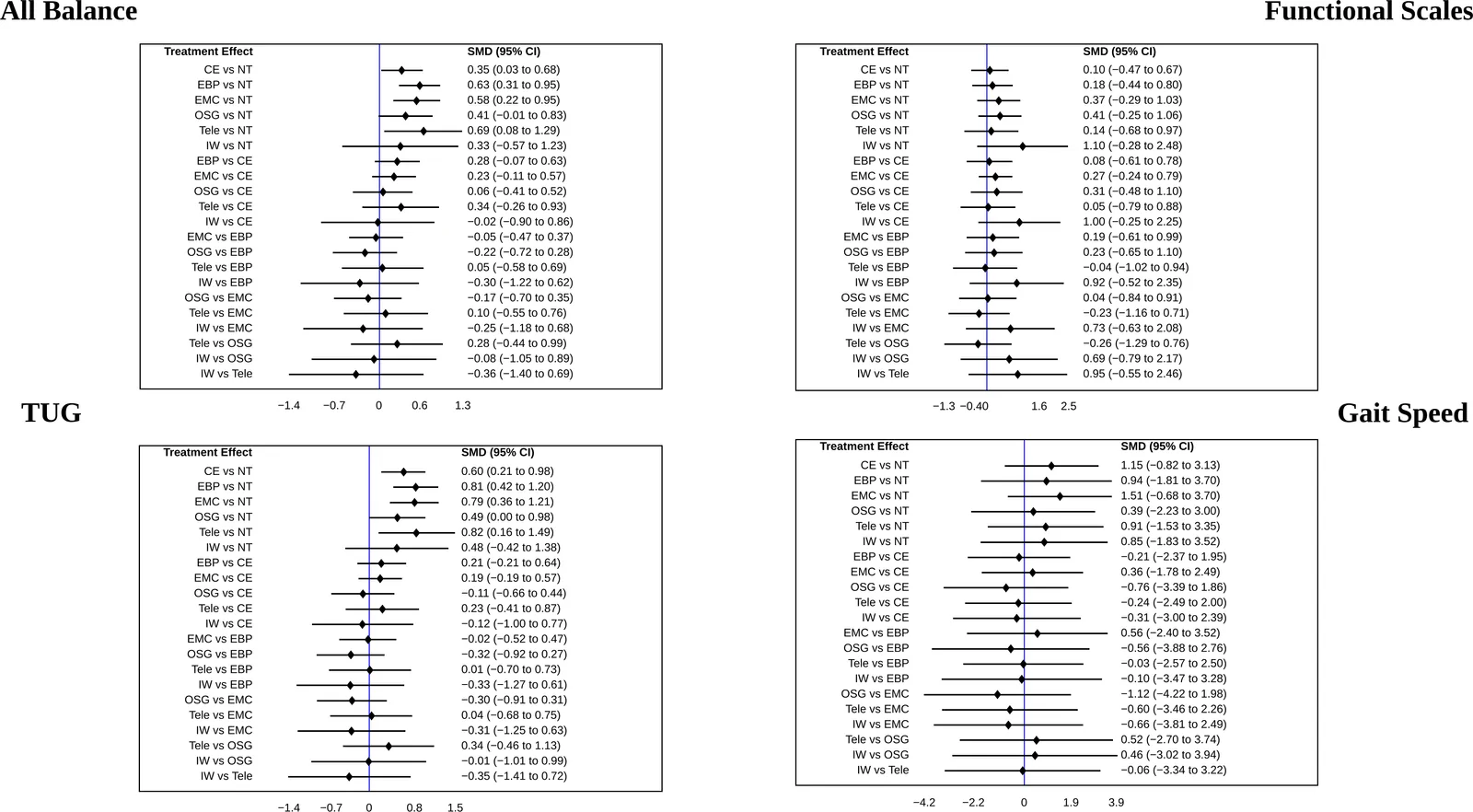
All-pairwise forest plots from the network meta-analysis consistency models for the 4 analytical networks. CE: conventional exercise; EBP: exergaming with balance platforms; EMC: exergaming with motion capture; IW: interventions with wearables; NT: no treatment; OSG: other serious games; Tele: telerehabilitation; TUG: Timed Up and Go.

**Table 3.** GRADE^a^ evidence profile for the network meta-analysis of technology-based rehabilitation interventions for balance and functional mobility in older adults (60 randomized controlled trials; 57 contributing data)^b^.

Comparison	Studies, n	Participants, n^c^	SMD^d^ (95% CI)	Reasons for rating down	Certainty
Network 1: all balance outcomes | 55 studies | global consistency test *χ*²_₁₃_=16.1; *P*=.25					
EBP^e^ versus NT^f^	21	1224	0.63 (0.31 to 0.95)	Risk of bias (serious); imprecision (serious)^g^	⊕⊕○○ Low
EMC^h^ versus NT	18	1153	0.58 (0.22 to 0.95)	Risk of bias (serious); imprecision (serious)^g^	⊕⊕○○ Low
Tele^i^ versus NT	5	991	0.69 (0.08 to 1.29)	Risk of bias (serious); imprecision (serious)^g^	⊕⊕○○ Low
OSG^j^ versus NT	10	1111	0.41 (−0.01 to 0.83)	Risk of bias (serious); imprecision (serious)	⊕⊕○○ Low
IW^k^ versus NT	3	822	0.33 (−0.57 to 1.23)	Risk of bias (serious); imprecision (very serious)	⊕○○○ Very low
CE^l^ versus NT	29	1374	0.35 (0.03 to 0.68)	Risk of bias (serious); imprecision (serious)^g^	⊕⊕○○ Low
EBP versus CE	21	1012	0.28 (−0.07 to 0.63)	Risk of bias (serious); imprecision (serious)	⊕⊕○○ Low
EMC versus CE	18	941	0.23 (−0.11 to 0.57)	Risk of bias (serious); imprecision (serious)	⊕⊕○○ Low
Tele versus CE	5	779	0.34 (−0.26 to 0.93)	Risk of bias (serious); imprecision (serious)	⊕⊕○○ Low
OSG versus CE	10	899	0.06 (−0.41 to 0.52)	Risk of bias (serious); imprecision (serious)	⊕⊕○○ Low
IW versus CE	3	610	−0.02 (−0.90 to 0.86)	Risk of bias (serious); imprecision (very serious)	⊕○○○ Very low
Network 2: functional scales | 26 studies | global consistency test *χ*²_₉_=13.8, *P*=.13					
IW versus NT	2	399	1.10 (−0.28 to 2.48)	Risk of bias (serious); imprecision (very serious)	⊕○○○ Very low
OSG versus NT	5	548	0.41 (−0.25 to 1.06)	Risk of bias (serious); imprecision (serious)	⊕⊕○○ Low
EMC versus NT	9	568	0.37 (−0.29 to 1.03)	Risk of bias (serious); imprecision (serious)	⊕⊕○○ Low
EBP versus NT	7	498	0.18 (−0.44 to 0.80)	Risk of bias (serious); imprecision (serious)	⊕⊕○○ Low
Tele versus NT	3	560	0.14 (−0.68 to 0.97)	Risk of bias (serious); imprecision (serious)	⊕⊕○○ Low
CE versus NT	15	671	0.10 (−0.47 to 0.67)	Risk of bias (serious); imprecision (serious)	⊕⊕○○ Low
IW versus CE	2	300	1.00 (−0.25 to 2.25)	Risk of bias (serious); imprecision (very serious)	⊕○○○ Very low
OSG versus CE	5	449	0.31 (−0.48 to 1.10)	Risk of bias (serious); imprecision (serious)	⊕⊕○○ Low
EMC versus CE	9	469	0.27 (−0.24 to 0.79)	Risk of bias (serious); imprecision (serious)	⊕⊕○○ Low
EBP versus CE	7	399	0.08 (−0.61 to 0.78)	Risk of bias (serious); imprecision (serious)	⊕⊕○○ Low
Tele versus CE	3	461	0.05 (−0.79 to 0.88)	Risk of bias (serious); imprecision (serious)	⊕⊕○○ Low
Network 3: Timed Up and Go test | 41 studies | global consistency test *χ*²_₁₂_=28.1; *P*=.005 (inconsistency localized to NT vs CE; all technology comparisons *P>*.08)					
EBP versus NT	14	940	0.81 (0.42 to 1.20)	Risk of bias (serious)	⊕⊕⊕○ Moderate
EMC versus NT	14	905	0.79 (0.36 to 1.21)	Risk of bias (serious)	⊕⊕⊕○ Moderate
Tele versus NT	4	823	0.82 (0.16 to 1.49)	Risk of bias (serious); imprecision (serious)	⊕⊕○○ Low
CE versus NT	22	1075	0.60 (0.21 to 0.98)	Risk of bias (serious); inconsistency (serious)^m^	⊕⊕○○ Low
OSG versus NT	7	878	0.49 (−0.005 to 0.978)	Risk of bias (serious); imprecision (serious)	⊕⊕○○ Low
IW versus NT	3	662	0.48 (−0.42 to 1.38)	Risk of bias (serious); imprecision (very serious)	⊕○○○ Very low
EBP versus CE	14	749	0.21 (−0.21 to 0.64)	Risk of bias (serious); imprecision (serious)	⊕⊕○○ Low
EMC versus CE	14	714	0.19 (−0.19 to 0.57)	Risk of bias (serious); imprecision (serious)	⊕⊕○○ Low
Tele versus CE	4	632	0.23 (−0.41 to 0.87)	Risk of bias (serious); imprecision (serious)	⊕⊕○○ Low
OSG versus CE	7	687	−0.11 (−0.66 to 0.44)	Risk of bias (serious); imprecision (serious)	⊕⊕○○ Low
IW versus CE	3	471	−0.12 (−1.00 to 0.77)	Risk of bias (serious); imprecision (very serious)	⊕○○○ Very low
Network 4: gait speed | 12 studies | global consistency test *χ*²_₆_=69.5; *P*<.001; supplementary and hypothesis-generating only					
EMC versus NT	3	285	1.51 (−0.68 to 3.70)	Risk of bias (serious); inconsistency (serious); imprecision (very serious)	⊕○○○ Very low
CE versus NT	7	367	1.15 (−0.82 to 3.13)	Risk of bias (serious); inconsistency (serious); imprecision (very serious)	⊕○○○ Very low
EBP versus NT	3	255	0.94 (−1.81 to 3.70)	Risk of bias (serious); inconsistency (serious); imprecision (very serious)	⊕○○○ Very low
Tele versus NT	3	388	0.91 (−1.53 to 3.35)	Risk of bias (serious); inconsistency (serious); imprecision (very serious)	⊕○○○ Very low
IW versus NT	2	244	0.85 (−1.83 to 3.52)	Risk of bias (serious); inconsistency (serious); imprecision (very serious)	⊕○○○ Very low
OSG versus NT	2	322	0.39 (−2.23 to 3.00)	Risk of bias (serious); inconsistency (serious); imprecision (very serious)	⊕○○○ Very low

a GRADE: Grading of Recommendations Assessment, Development, and Evaluation (⊕⊕⊕⊕ high, ⊕⊕⊕○ moderate, ⊕⊕○○ low, ⊕○○○ very low).

b All evidence begins at high certainty (randomized trials) and is rated down 1 level (serious) or 2 levels (very serious) per domain. Prespecified decision rules: imprecision was rated down 1 level when the 95% CI included the null or a trivial effect (|SMD|<0.20) and 2 levels when the CI additionally spanned appreciable benefit and appreciable harm (|SMD|>0.50) or fewer than 3 trials contributed; inconsistency was rated down where the design-by-treatment interaction test was significant and node splitting localized the discrepancy to the comparison concerned; indirectness was not rated down for any comparison, as all trials were randomized comparisons in the target population using the intended outcomes; and publication bias was not rated down for any comparison, as no comparison was supported by the ≥10 trials required for a meaningful test of funnel plot asymmetry. GRADE ratings were performed independently by 2 reviewers and finalized by consensus, following the framework adapted for network meta-analysis [15]; risk of bias was assessed with RoB 2 [25].

c Sum of arm-level n across contributing studies for both arms of each comparison.

d SMD: standardized mean difference.

e EBP: exergaming with balance platforms.

f NT: no treatment.

g In network 1, imprecision was judged against the 95% prediction interval rather than the CI because the between-study heterogeneity of the composite outcome (τ=0.597) means the CI understates the uncertainty applying to any individual balance instrument; all network 1 prediction intervals included the null.

h EMC: exergaming with motion capture.

i Tele: telerehabilitation.

j OSG: other serious games.

k IW: interventions with wearables.

l CE: conventional exercise.

m Rating down for inconsistency applies to CE vs NT only: node-splitting showed a significant discrepancy between direct (SMD 1.61) and indirect (SMD 0.19) evidence (*P*<.001), whereas all technology-based comparisons showed nonsignificant direct-indirect differences (all *P*>.08).

#### Network 1: All Balance Outcomes

The consistency model (55 studies; *τ*=0.597; τ²=0.356) identified Tele (SMD 0.69, 95% CI 0.08‐1.29; *P*=.03), EBP (SMD 0.63, 95% CI 0.31‐0.95; *P*<.001), and EMC (SMD 0.58, 95% CI 0.22‐0.95; *P*=.002) as significantly more effective than NT. CE was also significantly superior to NT (SMD 0.35, 95% CI 0.03‐0.68; *P*=.03). OSG showed a numerically positive but nonsignificant effect versus NT (SMD 0.41, 95% CI −0.01 to 0.83; *P*=.06), as did IW (SMD 0.33, 95% CI −0.57 to 1.23; *P*=.47), the latter based on only 3 trials. No technology-based intervention was statistically superior to CE. SUCRA-based ranking placed Tele first (76.3%), followed by EBP (75.2%), EMC (68.7%), OSG (47.2%), IW (41.2%), CE (36.6%), and NT (4.8%). The narrow separation between the 3 leading nodes (7.6 SUCRA percentage points) should be read together with the ranking probabilities reported in the Ranking Probabilities section, which show that no single intervention emerged as clearly best.

#### Network 2: Functional Scales

The consistency model (26 studies) yielded no statistically significant treatment contrasts. IW ranked first by SUCRA (88.9%), although this estimate rested on only 2 trials contributing 14 participants and carried a very wide CI (SMD 1.10, 95% CI −0.28 to 2.48; *P*=.12), substantially limiting its interpretability. OSG (SUCRA 62.9%), EMC (60.9%), EBP (43.4%), Tele (39.7%), CE (32.5%), and NT (21.7%) occupied subsequent positions.

#### Network 3: Timed Up and Go Test

The consistency model (41 studies) demonstrated that EBP (SMD 0.81, 95% CI 0.42‐1.20; *P*<.001), EMC (SMD 0.79, 95% CI 0.36‐1.21; *P*<.001), Tele (SMD 0.82, 95% CI 0.16‐1.49; *P*=.02), and CE (SMD 0.60, 95% CI 0.21‐0.98; *P*=.002) were each significantly superior to NT. OSG did not reach significance (SMD 0.49, 95% CI −0.005 to 0.978; *P*=.05), and neither did IW (SMD 0.48, 95% CI −0.42 to 1.38; *P*=.30). None of the technology-based interventions was significantly superior to CE. SUCRA ranking identified EBP (74.8%), Tele (72.5%), and EMC (71.7%) as the 3 highest-ranked interventions, separated by 3.1 percentage points.

#### Network 4: Gait Speed

The gait speed network (12 trials) was characterized by extreme between-study heterogeneity and very wide CIs for all treatment contrasts, none of which attained statistical significance. Given the significant global inconsistency and the extreme heterogeneity, these estimates are unsuitable for treatment-effect inference. They are reported as supplementary and hypothesis generating only, are not used to support any conclusion of this review, and should not be cited as evidence that these interventions do or do not affect gait speed.

### Inconsistency Testing

For network 1, the design-by-treatment interaction test was nonsignificant (*χ*²_₁₃_=16.1; *P*=.25), providing no evidence of meaningful inconsistency. Network 2 similarly showed no significant inconsistency (*χ*²_₉_=13.8; *P*=.13). Network 3 yielded a significant global statistic (*χ*²_₁₂_=28.1; *P*=.005). Node splitting localized this to the NT versus CE comparison exclusively (direct SMD 1.612, SE 0.331 vs indirect SMD 0.189, SE 0.205; difference 1.423; *P*<.001); all 11 remaining contrasts showed nonsignificant direct-indirect differences (all *P*>.08). Network 4 also demonstrated significant global inconsistency (*χ*²_₆_=69.5; *P*<.001).

Two features of the direct evidence bear on how this discrepancy should be read. First, it does not arise from a small number of aberrant results: the 4 trials that carry this contrast, which span 3 study designs, each produced a large positive direct effect (Hedges *g*=0.90, 1.09, 2.36, and 2.53; Table S7, Multimedia Appendix 1), against an indirect estimate of 0.19. Second, the 4 trials share a distinctive structure rather than a distinctive dose—each is a 3-arm trial containing an NT arm, each randomized only 11 to 18 participants per arm, and each prescribed CE 3 times per week, which is the median frequency across the 29 CE arms in the review.

Both sensitivity specifications attenuated every treatment estimate, and neither eliminated the discrepancy (Table S7 and Figure S3, Multimedia Appendix 1). Under specification 1, the direct-indirect difference for CE versus NT remained significant, and under specification 2, only a single trial contributed direct evidence, so the contrast was no longer estimable. The treatment hierarchy was largely preserved under specification 1 (Spearman ρ=0.86) and less so under specification 2 (ρ=0.68), where removal of 1 of only 4 Tele arms substantially destabilized that node. Across both specifications, the 2 comparisons carrying moderate certainty in the primary analysis—EBP versus NT and EMC versus NT—remained statistically significant. We therefore do not claim that consistency was restored by removing trials; the primary analysis retains all trials, and the CE versus NT comparison is instead rated down for inconsistency in the GRADE assessment.

### Transitivity Assessment

The distribution of the effect modifiers that could be extracted is summarized in Table S6 and Figure S2 in Multimedia Appendix 1. Across the 7 nodes of network 1, the mean of trial mean ages ranged from 70.7 years (Tele) to 78.6 years (IW), the mean intervention duration from 6.7 weeks (IW) to 30.0 weeks (Tele), and the mean session length from 33.8 minutes (Tele) to 50.0 minutes (IW). The 5 nodes supported by 10 or more trials (NT, CE, EBP, EMC, and OSG) were closely comparable to one another on every extracted modifier (mean age 71.7‐73.8 years; mean duration 7.7‐11.5 weeks; mean session length 38.8‐48.9 minutes), and the transitivity assumption appears defensible for comparisons among them.

Two nodes depart from this pattern and require caution. The Tele node rests on 5 trials and has a much longer mean intervention duration (30.0 wk) than any other node; this is driven entirely by a single 104-week trial [22], and the median Tele duration is 8 weeks. The IW node rests on 3 trials whose participants were on average 5 to 7 years older than those in the other nodes. Comparisons involving Tele and IW are therefore more vulnerable to violation of transitivity than comparisons among the remaining nodes and are interpreted accordingly throughout.

A further limitation of this appraisal is that several effect modifiers of recognized importance for balance outcomes could not be evaluated because they were reported too inconsistently across trials: baseline balance impairment, baseline fall risk or fall history, frailty status, sex distribution, the level of therapist supervision, the intensity of the CE comparator, prior familiarity with the technology, and the outcome measurement protocol. Residual intransitivity attributable to these unmeasured modifiers cannot be excluded, and the CE-intensity analyses reported below indicate that at least one of them—comparator intensity—does materially influence effect estimates.

### Meta-Regression of Training Dose

Post hoc random-effects meta-regressions found no statistically significant association between the technology versus control effect size and any training-dose parameter, in either the all-balance network or the TUG network (all *P*>.05; Table S10 and Figure S4, Multimedia Appendix 1). The inverse trend for session length and weekly training minutes approached but did not reach significance (*P*=.05-.10) and was opposite in direction to a simple “more-is-better” expectation, most plausibly reflecting confounding by baseline population severity: trials recruiting more impaired participants tended to prescribe longer sessions while achieving smaller standardized effects. As these analyses are based on study-level aggregate data, they are subject to ecological bias and are underpowered to detect modest dose-response relationships; the current evidence neither identifies an optimal training dose nor excludes one. What can be stated descriptively is the dose at which the observed effects were obtained: across the included trials, the median program ran for 8 weeks, with 3 sessions per week and 40 minutes per session. This describes the prescriptions that were tested rather than an established minimum effective dose, and the absence of a dose-response signal means it should not be read as a threshold below which benefit would be lost.

CE training frequency was associated with the magnitude of the direct CE versus NT effect (slope 1.46 per additional session per week; *P*=.04), while no other comparator-intensity parameter reached significance (Table S11 and Figure S5, Multimedia Appendix 1). This analysis rests on the 7 trials in network 1 that compared CE directly with NT and is hypothesis generating rather than confirmatory. It should also be noted that it does not, by itself, explain the direct-indirect discrepancy observed in the TUG network because all 4 trials contributing direct evidence to that contrast prescribed CE at the same frequency as the network median; comparator intensity is therefore a plausible general source of heterogeneity in the CE node, but not the specific explanation for the inconsistency detected in network 3.

### Ranking Probabilities

Probabilities of being the best treatment temper the interpretation of the SUCRA hierarchy (Table S12 and Figure S6, Multimedia Appendix 1). In the all-balance network, Tele had a probability of approximately 41% of being the best treatment, followed by EBP (approximately 22%), EMC (approximately 16%), IW (approximately 15%), and OSG (approximately 5%); the probabilities for CE and NT were negligible. In the TUG network, the corresponding values were approximately 36% for Tele, 26% for EBP, 21% for EMC, and 13% for IW. The substantial probability attaching to IW despite its fifth-place SUCRA position arises from the extreme width of its CI (−0.57 to 1.23 in the all-balance network, 3 trials, 29 participants) rather than from any robust signal of benefit, and this illustrates why ranking metrics must not be read independently of effect size, precision, and certainty of evidence. No intervention emerged as clearly the best option, and the SUCRA hierarchy should not be read as a definitive ordering of clinical effectiveness.

### Small-Study Effects and Publication Bias

For networks 1 and 3, visual inspection of comparison-adjusted funnel plots suggested broad symmetry around the comparison-specific pooled effects. Network 2 exhibited marginal asymmetry in the IW comparisons (Figure 5). Network 4 showed notable asymmetry. However, networks 2 and 4 contribute only 26 and 12 trials, respectively, across 7 nodes, and no comparison in either network is supported by the 10 trials conventionally regarded as the minimum for a meaningful test of funnel plot asymmetry. The apparent asymmetry in these 2 networks is therefore uninterpretable, and no comparison was rated down for publication bias on this basis; the possibility of unpublished negative trials, particularly for the sparsely studied IW and OSG categories, cannot be excluded and is treated as an unresolved limitation rather than as a graded judgment.

**Figure 5.**
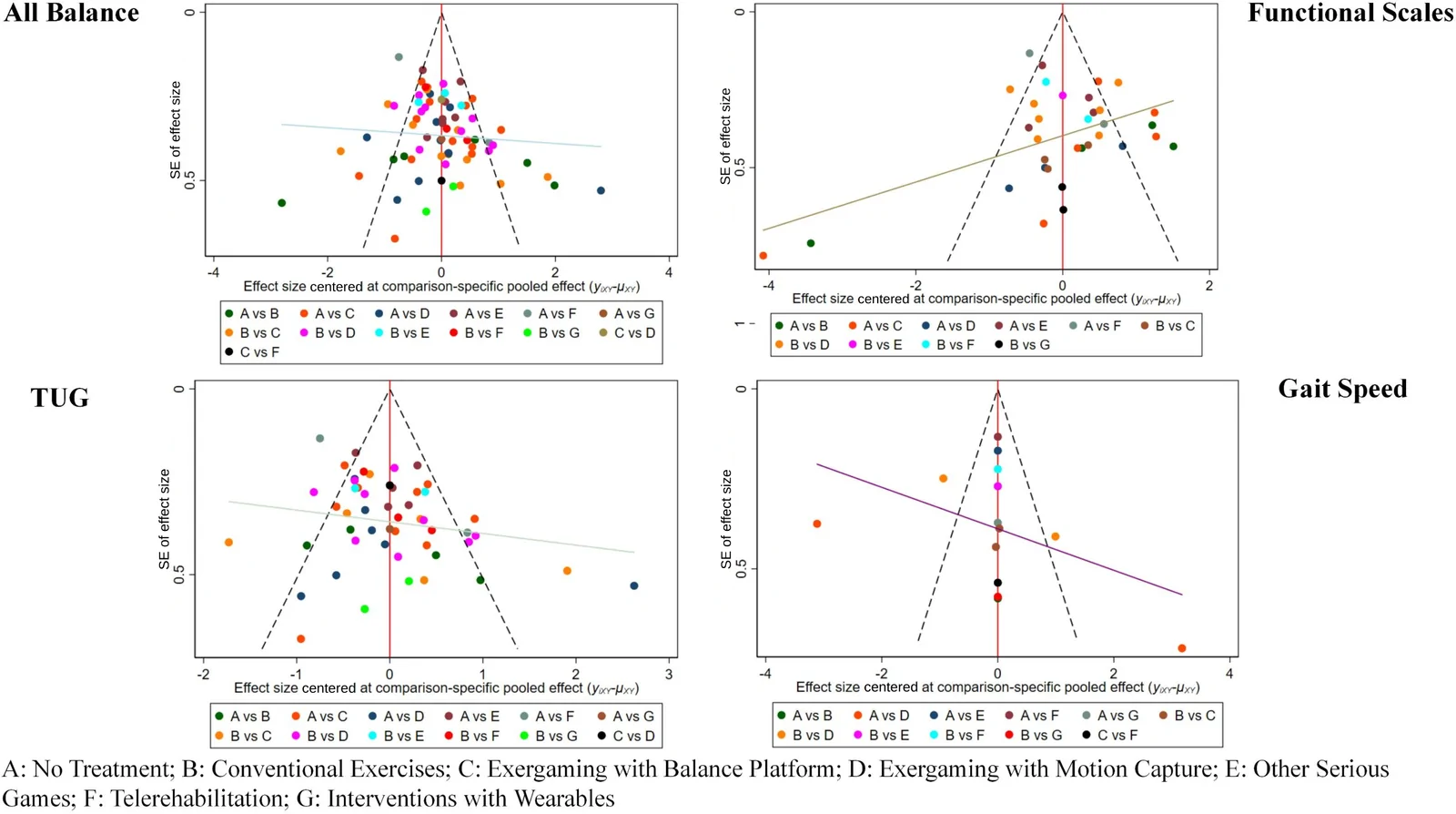
Comparison-adjusted funnel plots for assessment of small-study effects and publication bias in the 4 analytical networks. TUG: Timed Up and Go.

### Certainty of Evidence

GRADE ratings are summarized in Table 3. Certainty was rated moderate for only 2 comparisons—EBP versus NT and EMC versus NT in the TUG network—both of which were rated down 1 level for risk of bias and met the prespecified precision criterion without further downgrading. Every other comparison in the review was rated low or very low. In network 1, all comparisons were rated down for imprecision because the 95% prediction interval included the null, reflecting the substantial between-study heterogeneity inherent to a composite balance outcome (*τ*=0.597). All comparisons involving IW were rated very low owing to very serious imprecision arising from 3 or fewer contributing trials. All network 4 comparisons were rated very low, being rated down for both very serious imprecision and serious inconsistency. No comparison was rated down for indirectness, and, for the reasons given above, none was rated down for publication bias. The explicit judgment reached for each of the 5 GRADE domains, for every comparison rated, is tabulated in Table S13 in Multimedia Appendix 1.

### Acceptability, Adverse Events, and User Burden

Dropout data were available for 59 (98.3%) of the 60 included trials (all except 1 [52], for which attrition was not reported). The crude dropout rates per category were 12.5% (69/552) for EBP, 12.4% (55/443) for EMC, 15.7% (34/217) for Tele, 12% (4/33) for IW, 10.5% (31/295) for OSG, 11.5% (78/676) for CE, and 10.6% (87/823) for NT. These denominators are the numbers of participants randomized to arms that both reported attrition and were assignable to a single network node; they therefore sum to 3039 rather than to the 3469 participants randomized overall. Pairwise odds ratios for all 5 technology categories versus CE were nonsignificant (all *P*>.05; Figure 6), with a pooled overall estimate of odds ratio 1.05 (95% CI 0.76‐1.43; *I*²=0%). When NT was included as an additional control condition, the pooled overall odds ratio for technology-based interventions versus all control arms was 1.10 (95% CI 0.87‐1.40; *P*=.41; *I*²=0%), indicating no statistically significant difference in acceptability between technology-based and conventional rehabilitation (Figures 6 and 7).

Reporting of the other feasibility outcomes was too sparse to permit synthesis. Only 3 (5%) of the 60 included trials reported a formal safety or adverse event outcome, and only 3 (5%) reported participant satisfaction or acceptability using a defined instrument; technology-related difficulties such as device malfunction, connectivity failure, or cybersickness were reported in a structured manner by a single trial [19], which used the Virtual Reality Sickness Questionnaire. Where adverse events were described, they were minor and were not attributed to the technology itself. The near-absence of standardized reporting of harms, usability, and satisfaction is an important evidence gap: for interventions whose principal claimed advantage is accessibility and engagement, these outcomes are as decision relevant as the effect on balance, and dropout alone is an inadequate proxy for them.

**Figure 6.**
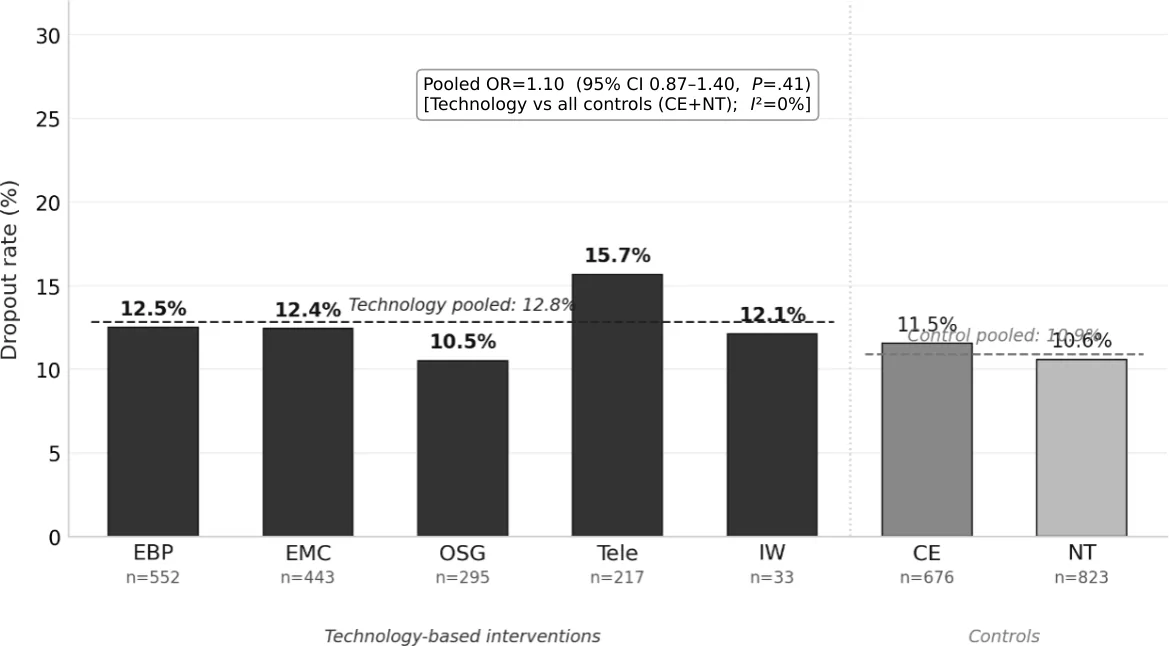
Pooled crude dropout rates by intervention category across all included trials with available dropout data (*N* below each bar denotes the total number of participants randomized per category). CE: conventional exercise; EBP: exergaming with balance platforms; EMC: exergaming with motion capture; IW: interventions with wearables; NT: no treatment; OR: odds ratio; OSG: other serious games; Tele: telerehabilitation.

**Figure 7.**
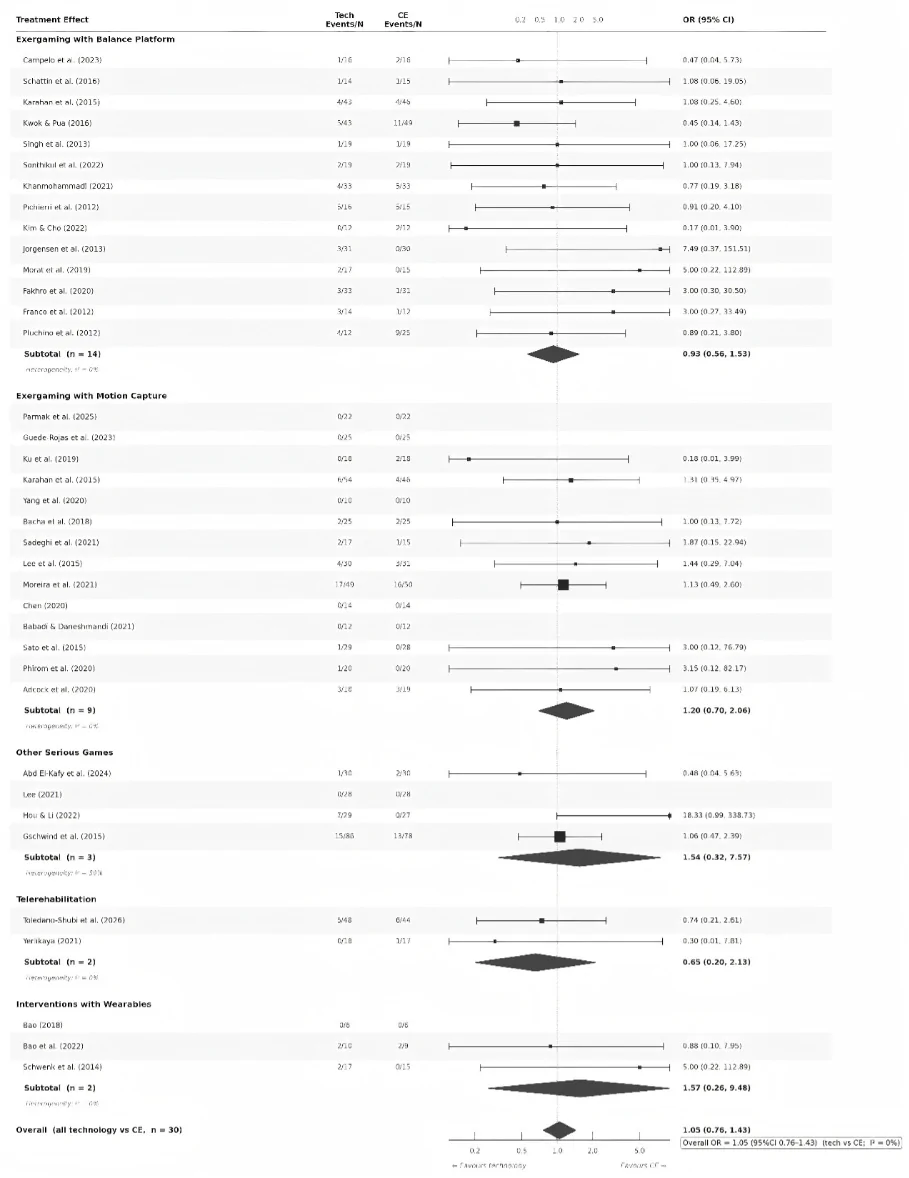
Forest plot of all-cause dropout rates comparing each technology-based intervention category against conventional exercises [18,19,34-36,41,42,44-47,49-51,54,55,58,60-68,73,74,77,79,82-86]. CE: conventional exercise; EBP: exergaming with balance platforms; EMC: exergaming with motion capture; IW: interventions with wearables; OR: odds ratio; OSG: other serious games; Tele: telerehabilitation

## Discussion

### Principal Findings

This update includes 60 RCTs, of which 57 (95%) contributed data, and extends the evidence base by approximately 2 years beyond the original review [12]. Three findings stand out.

First, more technologies now show a benefit. The original review identified only EMC as statistically superior to no treatment, and only for TUG; here EBP, EMC, and Tele each improved outcomes significantly versus NT in both the all-balance composite and the TUG networks. This reflects the entry of larger, better-powered trials, particularly in the Tele and EBP categories, into nodes that previously lacked the precision to detect plausible effects.

Second, the treatment hierarchy has shifted. Tele rose from fifth of 7 ranked interventions in the original NMA (SUCRA 43%) to first in the all-balance network. However, the node contains only 5 trials and 198 participants, and Tele’s probability of being the best treatment is approximately 41%, so this ranking is a hypothesis awaiting confirmation rather than an established hierarchy.

Third, GRADE appraisal, applied here for the first time in this technology category, shows that certainty is moderate at best and moderate for only 2 (5.1%) of the 39 pairwise comparisons rated. Risk of bias in the underlying literature is the main reason. The impossibility of blinding participants to an exercise intervention contributes to this, but it is not the whole explanation: blinded outcome assessment was absent or unclear in a substantial minority of trials, and fewer than half reported a prespecified protocol. Both deficiencies are remediable, and the moderate ratings for EBP and EMC versus NT in the TUG network show that the threshold is reachable in this field.

### Telerehabilitation: Highest Ranked but Preliminary

Tele ranked highest across several outcome networks in this update, but the wide CI (SMD 0.69, 95% CI 0.08‐1.29) and the small number of contributing trials call for caution. In the original review, only 3 Tele trials were available [12]. The addition of further large RCTs since 2023 contributed to statistically significant effects versus NT in both network 1 and network 3, consistent with a 2024 systematic review by Gamble et al [88], which found Tele to be noninferior to control interventions across 26 RCTs. The large randomized sample sizes of Tele trials (mean 124 participants per trial) confer greater statistical power per study, which may partly explain the elevation of the SUCRA ranking [22]. Three features of the evidence argue for restraint. The node rests on 5 trials, its 95% CI extends from a trivial effect (0.08) to a very large one (1.29), and its mean intervention duration is the longest and least typical of any node, driven by a single 104-week trial. The sensitivity analysis reported earlier, which removed 1 of the 4 Tele arms in the TUG network, widened the estimate further. Tele is therefore promising and highest ranked, but not demonstrably superior.

Tele works through a different mechanism from exergaming: expert supervision, error correction, and motivational support delivered in real time, which reproduces the cognitive behavioral elements of face-to-face physiotherapy without the access barriers [89,90]. The finding that Tele was not statistically superior to CE in any network is consistent with this and suggests a role as a scalable complement to in-person care rather than a replacement for it.

### Convergence of Evidence for Exergaming Platforms and Motion Capture

EBP and EMC maintained significant effects versus NT across both the all-balance composite and TUG networks, with GRADE ratings of moderate certainty for EBP versus NT (SMD 0.81, 95% CI 0.42‐1.20) and EMC versus NT (SMD 0.79, 95% CI 0.36‐1.21) in network 3—the only moderate-certainty findings in this NMA. The comparable effect sizes of EBP and EMC are consistent with a recent meta-analysis [91] that found no significant difference between platforms, reflecting their shared therapeutic mechanism of real-time visual augmented feedback enabling immediate postural error correction [6]. Clinicians may reasonably select between them based on practical considerations, including equipment cost, installation requirements, and participant mobility constraints.

### Serious Games and Wearable Interventions: Persistent Evidence Gaps

OSG fell just short of significance versus NT in network 3 (SMD 0.49, 95% CI −0.005 to 0.978; *P*=.05). This estimate should not be reported as a positive finding, and the accompanying certainty rating is low. Despite being purpose-built rehabilitation software, OSG did not outperform commercially available exergaming platforms in any network, suggesting that therapeutic benefits may depend more on movement engagement and practice volume than on software sophistication [92,93].

IW similarly failed to reach statistical significance in any comparison. The extreme width of the IW CIs reflects the small number of eligible trials (n=3), all involving custom-designed haptic or vibrotactile feedback systems. This gap has persisted over more than a decade of published work and is unlikely to reflect a lack of researcher interest. Three structural barriers are plausible. First, the wearable systems evaluated to date have been bespoke research prototypes requiring specialist calibration and supervision, which limits both trial size and clinical scalability; commercial devices with validated balance-feedback functions have only recently become available. Second, feedback modalities remain unstandardized—vibrotactile, auditory, and visual channels are used interchangeably across trials, so that the IW node aggregates mechanistically dissimilar interventions. Third, the cost of custom sensor arrays has kept sample sizes small: the 3 IW trials contributing to this review randomized 12, 16, and 33 participants, respectively. These findings indicate insufficient investigation rather than inefficacy. The priority for this category is agreement on a standardized feedback modality and outcome set that would make future trials poolable, not further small pilot trials [13].

### The All-Balance Composite Network

Network 1 demonstrates that Tele, EBP, EMC, and CE each produced statistically significant improvements relative to NT (SMDs 0.35‐0.69). The narrow span of effects across technically distinct platforms suggests a common minimum benefit achievable through any adequately structured balance program, whatever the delivery mechanism. No technology exceeded CE in any network, consistent with technology acting as a delivery vehicle rather than a source of superior physiological stimulus.

How far this network can be interpreted is nevertheless limited by its construction. It pools instruments that measure genuinely different constructs—dynamic functional mobility (TUG), multidimensional clinical rating scales (BBS, Mini-BESTest, and SPPB), static postural sway (COP parameters and single-leg stance), and lower-limb function (sit-to-stand)—into a single standardized effect. A composite SMD of 0.63 does not tell a clinician which dimension of balance improved, and the substantial between-study heterogeneity (*τ*=0.597) is in part a direct expression of this structural pooling rather than of true clinical variability. For this reason, the prediction interval rather than the CI was used to rate imprecision in this network; every network 1 comparison is rated low or very low certainty, and the principal clinical conclusions of this review rest on network 3 (TUG), which is a single-construct outcome, is supported by the largest number of trials of any single instrument, and yields the only moderate-certainty estimates in the review. Network 1 therefore tests whether any balance benefit is detectable across the evidence base as a whole, while network 3 provides the estimate on which practice decisions should be based.

### Outcome-Specific Results: Instrument Sensitivity and Task Alignment

The absence of significant effects in network 2 (functional scales) probably reflects the differential sensitivity of these instruments to the adaptations induced by short- to medium-duration technology-based training. The TUG mirrors the movement demands of many such programs, particularly sit-to-stand transitions, walking, and turning, giving it high construct validity and sensitivity to training-induced change [94]. Multidimensional scales such as the BBS, Mini-BESTest, and SPPB instead span a broader range of postural control domains, not all equally targeted by a typical exergaming program [95]. Future trials should adopt the TUG as the primary outcome for functional mobility, supplemented by domain-specific subscales where appropriate.

Network 3 (41 trials) yields the most internally consistent pattern of results and constitutes the primary basis for the moderate GRADE certainty ratings assigned to EBP and EMC. EBP (SMD 0.81), EMC (0.79), Tele (0.82), and CE (0.60) were each significantly superior to NT, while OSG (0.49) narrowly missed significance. The point estimates for EBP, EMC, and Tele were near-identical despite the mechanistically distinct pathways through which these platforms operate. The procedural demands of the TUG (rising from a chair, walking 3 m, turning, returning, and sitting) map directly onto the movement content targeted by all 3 platforms: balance board exergaming trains the postural transitions; motion capture training reinforces walking and turning kinematics; and Tele exercises typically incorporate functional mobility sequences supervised in real time. This task-training alignment plausibly explains why the TUG detects significant effects across a broader range of interventions than the functional scales network.

The gait speed network continued to yield no statistically significant treatment contrasts. This null result has now persisted across 2 successive NMAs, which points to a measurement problem: included trials used heterogeneous protocols (4-m vs 10-m tests, different starting conditions, single vs repeated trials), making meaningful pooling inherently difficult. Future trials should standardize gait speed assessment according to published consensus guidelines and should ideally supplement single-task gait speed with dual-task walking assessments, which may be more sensitive to the cognitive-motor integration benefits of technology-based training [96]. Until such standardization is achieved, gait-speed network estimates of this kind should not be used to draw treatment-effect conclusions.

### Conventional Exercise as a Problematic Comparator

The significant global inconsistency in network 3, precisely localized by node splitting to the NT versus CE comparison (direct SMD 1.612 vs indirect SMD 0.189; *P*<.001), has important methodological implications. The aggregation of diverse exercise protocols into a single CE node implicitly assumes a uniform CE versus NT effect across study contexts. This assumption was violated.

The pattern of the underlying data constrains which explanations are tenable. The discrepancy is not driven by 1 or 2 aberrant trials: all 4 trials contributing direct CE versus NT evidence produced large effects (*g=0.90*‐2.53; Table S7, Multimedia Appendix 1). Nor is it explained by comparator intensity, the hypothesis that the meta-regression reported earlier might be thought to support, because all 4 of these trials prescribed CE 3 times per week, the median frequency in the review. What the 4 trials do share is a structural feature: each is a small 3-arm trial (11‐18 participants per arm) in which an NT arm ran alongside an active technology arm. Two mechanisms plausibly follow. Small-study effects would inflate all contrasts in such trials, and the direct CE versus NT evidence rests exclusively on them, whereas the indirect estimate is dominated by larger 2-arm technology versus CE trials. In addition, participants allocated to no treatment within a trial in which others visibly receive an engaging technology may disengage in a way that participants in a 2-arm design do not, depressing the NT arm and inflating every within-trial contrast against it.

The consequence is that removing the discrepant trials does not resolve the inconsistency: it attenuates every estimate, and the direct-indirect difference persists under the less aggressive threshold and becomes inestimable under the more aggressive one (Table S7, Multimedia Appendix 1). We therefore do not claim that the sensitivity analysis restored consistency, nor use it to justify the primary estimates. It supports only the narrower claim that the 2 comparisons on which our clinical conclusions rest, EBP versus NT and EMC versus NT in the TUG network, remain significant under every specification examined. The CE versus NT contrast itself is the least reliable estimate in the review, is rated down for inconsistency in GRADE accordingly, and should not be cited as this review’s estimate of the effect of CE. The remedy is prospective. Future NMAs in this field should prespecify minimum comparator-intensity requirements or stratify the CE node, as emphasized in a 2024 Delphi process [97], and trials comparing a technology with no treatment should report enough detail about the NT condition for its adequacy as a common comparator to be judged.

### Acceptability

In contrast to the original review by Cieślik et al [12], which reported a marginally but significantly higher dropout probability in technology-based interventions versus controls (odds ratio 1.22, 95% CI 1.03‐1.45; *P*=.02), the present updated analysis found no statistically significant difference in dropout rates between technology-based interventions and all control conditions (pooled odds ratio 1.10, 95% CI 0.87‐1.40; *P*=.41; *I*²=0%). This discrepancy likely reflects the expansion of the evidence base: the addition of 10 trials published since the original search, several of which reported zero or near-zero dropout, attenuated the modest signal detected in the smaller original dataset. Acceptability was comparable across all 5 technology categories, and the marginally elevated dropout risk identified in 2023 does not appear to be a stable feature of this intervention class as the literature matures.

The numerically highest crude dropout rate, observed in the Tele category (15.7%), did not differ significantly from that for CE (odds ratio 0.65, 95% CI 0.20‐2.13) and reflects the composition of that node rather than poorer tolerability: it is driven by a single 104-week trial [22] whose comparator was no treatment, so that its attrition contributes to the crude Tele rate but not to the within-trial Tele versus CE comparison. In the trials that did compare Tele directly with CE, dropout was numerically lower in the Tele arms. A 2024 systematic review found high overall adherence (median >80%) to technology-based programs, with technology-related dropout accounting for only 3% of total withdrawals [98,99].

### GRADE Certainty and Clinical Translation

This is the first formal certainty-of-evidence assessment for this technology category in older adults. The moderate ratings for EBP versus NT and EMC versus NT in the TUG network would support a conditional recommendation for EBP or EMC as options—not as proven superior alternatives—relative to no treatment for functional mobility. A recommendation of any strength, however, requires more than a certainty rating: a full GRADE Evidence-to-Decision appraisal [100] must weigh 3 further domains that this evidence base addresses only weakly.

On benefits and harms, the TUG effects for EBP and EMC (SMD approximately 0.8) are clinically appreciable, and attrition showed no excess; but harms were formally reported by only 3 (5%) of 60 trials, so that side of the balance rests on absence of data rather than evidence of safety. On values and preferences, no trial elicited older adults’ preferences between technology-delivered and face-to-face exercise, and the choice is unlikely to be preference-neutral: some older adults value the social contact of supervised group exercise, whereas others value avoiding travel. As no technology exceeded CE, preference should carry substantial weight where both are available. On resource use, the technologies differ by more than an order of magnitude in acquisition, maintenance, and training costs, from consumer game consoles to bespoke sensor arrays, and no trial reported a cost outcome. In practical terms, where supervised CE is accessible and acceptable, this evidence provides no basis for substituting a technology-based program for it. These technologies are best placed to reach people for whom in-person supervised exercise is not accessible, the group for whom the comparison against no treatment, which carries the highest certainty, is the relevant one.

The predominance of low-certainty ratings reflects a structural feature of the current exercise rehabilitation literature rather than an absence of effect. As blinding of participants to an exercise intervention is not achievable, future GRADE assessments in this field would be better served by domain-specific rules that ask whether the absence of blinding plausibly affected adherence, cointerventions, outcome assessment, or selective reporting in the trial concerned, rather than by applying an automatic downgrade [25]. Applying that reasoning here, the remediable deficiencies are blinded outcome assessment and protocol prespecification, both of which are entirely feasible in this trial design.

### Limitations

This review has several limitations. First, aggregation of heterogeneous exercise protocols within CE and within each technology node introduces within-node heterogeneity that the NMA model cannot fully accommodate; the CE-intensity meta-regression quantifies this for the comparator node, but analogous data were not available for every technology node. Second, IW and Tele remain underpowered relative to EBP and EMC, and the apparent elevation of Tele to first rank awaits confirmation. Third, restriction to postintervention means and SDs excluded change-score studies. Fourth, the very small number of head-to-head active-technology comparisons means that most technology versus technology estimates are wholly indirect and therefore depend on the transitivity assumption examined earlier. Fifth, several recognized effect modifiers—baseline balance impairment, fall history, frailty, sex distribution, supervision level, and technology familiarity—were too inconsistently reported to be assessed, so residual intransitivity cannot be excluded. Sixth, effect sizes were computed from analyzed rather than randomized denominators, which may introduce attrition bias in the minority of trials with substantial loss to follow-up. Finally, the gait speed network remained uninterpretable owing to extreme heterogeneity.

The absence of long-term follow-up data is a further limitation, and a clinically important one. All analyses used postintervention measurements obtained at the end of the training period; no study contributed follow-up data (eg, at 3, 6, or 12 mo after intervention cessation) to the quantitative synthesis. The present conclusions therefore apply to immediate, end-of-intervention effects and cannot be extrapolated to the durability of benefit or to long-term fall prevention outcomes. Durability is central to the clinical value of fall prevention interventions, and future trials should include follow-up of at least 6 months and report the trajectory of effects after training ends.

Related to this, the outcomes synthesized here are measures of balance, functional mobility, and gait speed, not of falls. No included trial contributed data on fall incidence, fall rate, injurious falls, or time to first fall to any network. The interventions studied may plausibly reduce fall risk through the balance and mobility improvements demonstrated, and the instruments used are established predictors of falls, but this review provides no direct evidence that they prevent falls, and its findings should not be cited to that effect.

### Implementation Considerations

Several practical considerations will determine whether these interventions can be deployed effectively in real-world aging care settings. Access to suitable devices and connectivity, digital literacy, usability for older users, the level of therapist or caregiver supervision required, data privacy, equipment and maintenance costs, and the digital divide among older adults may all influence uptake, adherence, and equity of access. These factors vary substantially across the technologies compared here and should be weighed alongside effectiveness when considering implementation, particularly for Tele and wearable systems whose real-world use may differ from the supervised conditions of the included trials.

### Future Directions

The Tele evidence base most urgently requires expansion through large, rigorous RCTs that standardize the definition and intensity of remotely supervised exercise protocols. Standardizing the categorization of CE intensity across technology trial designs would substantially reduce the inconsistency problem identified in the TUG network. Trials should prioritize blinded outcome assessment, prospectively register their protocols, adopt at least 6 months of postintervention follow-up, report falls alongside balance outcomes, and report adverse events, technology-related difficulties, and participant satisfaction using standardized instruments. Two further design priorities follow from the present findings. First, instrumented or otherwise objective measurement of balance and functional mobility, applied by assessors blinded to allocation, would reduce the observer susceptibility that is inherent in timed and rated clinical tests and is the principal remediable source of risk of bias in this literature. Second, the near-absence of direct comparisons between technology categories means that almost every technology versus technology estimate reported here is wholly indirect; head-to-head trials, even of modest size, would contribute disproportionately to the precision and the certainty of the network.

Two further directions are worth noting. Individual participant data meta-analysis would overcome the ecological limitations of the study-level dose meta-regressions reported here and is the most promising route to identifying an optimal training dose. AI-adaptive exercise systems, in which machine learning continuously matches prescription to a dynamically assessed individual profile, form an emerging category that fell outside the present taxonomy because the available studies use outcome measures and designs that are not commensurable with network pooling [101-103]; incorporating them into future syntheses will require prior agreement on a core outcome set for digitally delivered exercise rather than further primary trials alone.

### Conclusions

The evidence that technology-assisted rehabilitation improves balance and functional mobility in older adults has strengthened since the original 2023 review. EBP, EMC, and Tele each produced statistically significant improvements in functional mobility and overall balance versus no treatment. The emergence of Tele as the highest-ranked intervention by SUCRA is a preliminary observation resting on 5 trials with a wide CI and a probability of being best of only approximately 41%; it requires confirmation in larger trials. Moderate GRADE certainty for EBP and EMC versus no treatment on the TUG test may support their conditional use as options, rather than as proven superior alternatives, for improving functional mobility where supervised in-person exercise is not accessible; no technology exceeded CE in any network, and the low to very low certainty of the remaining 37 comparisons is insufficient to confirm effectiveness. As no included trial contributed fall outcomes, these findings should not be interpreted as direct evidence of fall prevention. Consolidating these findings will require rigorously designed, adequately powered RCTs, particularly for Tele, wearable interventions, and head-to-head technology comparisons, with standardized comparator intensity, blinded outcome assessment, and follow-up beyond the end of training.

## Supplementary material

10.2196/101231 Multimedia Appendix 1 Supplementary tables and figures providing additional methodological details and results of the systematic review and network meta-analysis.

10.2196/101231 Checklist 1 PRISMA 2020 checklist.

10.2196/101231 Checklist 2 PRISMA-NMA checklist.

## Abbreviations

BBS: Berg Balance Scale
CE: conventional exercise
COP: center of pressure
EBP: exergaming with balance platforms
EMC: exergaming with motion capture
FAB: Fullerton Advanced Balance Scale
GRADE: Grading of Recommendations Assessment, Development, and Evaluation
IW: interventions with wearables
Mini-BESTest: Mini-Balance Evaluation Systems Test
NMA: network meta-analysis
NT: no treatment
OSG: other serious games
POMA: Performance-Oriented Mobility Assessment
PRISMA: Preferred Reporting Items for Systematic Reviews and Meta-Analyses
PRISMA-NMA: Preferred Reporting Items for Systematic Reviews and Meta-Analyses extension for network meta-analyses
PROSPERO: International Prospective Register of Systematic Reviews
RCT: randomized controlled trial
RoB 2: Cochrane Risk of Bias tool, version 2
SMD: standardized mean difference
SPPB: Short Physical Performance Battery
SUCRA: surface under the cumulative ranking curve
Tele: telerehabilitation
TUG: Timed Up and Go
