# Supplementary material for: Comparative Effectiveness of Digital Exercise-Based Interventions for Improving Balance in Older Adults: Systematic Review and Network Meta-Analysis With GRADE Certainty of Evidence
Source: JMIR Aging. 2026 Oct 28;9:e101231. doi: 10.2196/101231 (PMC13717434; doi:10.2196/101231)
Supplement: Supplementary file 1 [file aging-v9-e101231-s001.docx]

# **Multimedia Appendix 3**

*Comparative Effectiveness of Digital Exercise-Based Interventions for Improving Balance in Older Adults: A Network Meta-Analysis With GRADE Certainty of Evidence*

Tables and figures in this appendix are numbered in the order in which they are first cited in the main text. Figures S1-S6 are supplied as separate image files; all 13 tables follow below. Two trials by Gschwind et al were published in 2015 and are distinguished throughout as Gschwind et al (2015a) (n=148; comparison of 2 exergame interventions; reference 67) and Gschwind et al (2015b) (n=153; iStoppFalls; reference 68). Every estimate that also appears in the main text is reproduced here exactly as reported in Tables 2 and 3.

## **Table S1. Database search strategies**

Strategies are reproduced exactly as run. The primary search covered inception to April 2025; the supplementary update search of 31 January 2026 reran each strategy unchanged. Record counts are the combined yield of the 2 searches.

| **Database (platform)** | **Date executed** | **Full strategy as run** | **Records** |
| --- | --- | --- | --- |
| PubMed | Primary: April 2025 Supplementary: 31 Jan 2026 | (((((((((((((((((((((((((((((((((((((((("Aged"[MeSH Terms] OR "older"[All Fields] OR "elder*"[All Fields] OR "senior"[All Fields]) NOT "stroke"[All Fields]) NOT "parkinson's"[All Fields]) NOT "parkinson"[All Fields]) NOT "Chronic Obstructive Pulmonary Disease"[All Fields]) NOT "COPD"[All Fields]) NOT "cardiac"[All Fields]) NOT "pulmonary"[All Fields]) NOT "neurologic"[All Fields]) NOT "hemiplegia"[All Fields]) NOT "paraplegia"[All Fields]) NOT "neuropathy"[All Fields]) NOT "arthroplasty"[All Fields]) NOT "kidney"[All Fields]) NOT "vestibular loss"[All Fields]) NOT "amblyopia"[All Fields]) NOT "fracture"[All Fields]) NOT "Meniere's"[All Fields]) NOT "breast cancer"[All Fields]) NOT "cancer"[All Fields]) NOT "replacement"[All Fields]) NOT "Alzheimer's"[All Fields]) NOT "dementia"[All Fields]) NOT "down syndrome"[All Fields]) NOT "low back pain"[All Fields]) NOT "amputation"[All Fields]) NOT "Huntington disease"[All Fields]) NOT "multiple sclerosis"[All Fields]) NOT "insomnia"[All Fields]) NOT "Fibromyalgia"[All Fields]) NOT "cochleovestibular loss"[All Fields]) NOT "osteoarthritis"[All Fields]) NOT "osteoporosis"[All Fields]) NOT "surgery"[All Fields]) NOT "Diabetes"[All Fields]) NOT "review"[All Fields]) NOT "meta-analysis"[All Fields]) NOT "study protocol"[All Fields]) NOT "quasi-randomized"[All Fields]) NOT "Cerebral palsy"[All Fields]) AND ("Virtual Reality"[MeSH Terms] OR "Virtual Reality Exposure Therapy"[MeSH Terms] OR "Exergaming"[MeSH Terms] OR "game-based rehabilitation"[All Fields] OR "head mounted display"[All Fields] OR "commercial video gam*"[All Fields] OR "video gam*"[All Fields] OR "Virtual Reality"[All Fields] OR "Nintendo"[All Fields] OR "WiiPD"[All Fields] OR "WII fit"[All Fields] OR "Wii-hab"[All Fields] OR "Xbox"[All Fields] OR "PlayStation"[All Fields] OR "Kinect"[All Fields] OR "VR"[All Fields] OR "exergam*"[All Fields] OR "virtual reality exercis*"[All Fields] OR "active video gam*"[All Fields] OR "serious gam*"[All Fields] OR "augmented reality"[All Fields] OR "mixed reality"[All Fields] OR "Telemedicine"[MeSH Terms] OR "Telemedicine"[All Fields] OR "Telehealth"[All Fields] OR "Telerehabilitation"[All Fields] OR "videoconferenc*"[All Fields] OR "Teletherapy"[All Fields] OR "teleconferenc*"[All Fields] OR "Telepractice"[All Fields] OR "teletreatment*"[All Fields] OR "Virtual conference"[All Fields] OR"ehealth"[All Fields] OR "artificial intelligence"[All Fields] OR "AI-assisted"[All Fields] OR "machine learning"[All Fields] OR "deep learning"[All Fields] OR "reinforcement learning"[All Fields] OR "intelligent tutoring"[All Fields] OR "adaptive feedback"[All Fields] OR "AI-based rehabilitation"[All Fields] OR "AI-powered"[All Fields] OR "robot-assisted"[All Fields] OR "intelligent rehabilitation"[All Fields] OR "smart rehabilitation"[All Fields] OR "sensor-based feedback"[All Fields] OR "personalized feedback"[All Fields] OR "automated feedback"[All Fields] OR "digital therapeutics"[All Fields] OR "conversational agent"[All Fields] OR "chatbot"[All Fields] OR "virtual agent"[All Fields] OR "virtual coach"[All Fields] OR "embodied agent"[All Fields]) AND ("gait"[MeSH Terms] OR "Postural Balance"[MeSH Terms] OR "gait analysis"[MeSH Terms] OR "Walk Test"[MeSH Terms] OR "postural stability"[All Fields] OR "balance"[All Fields] OR "gait"[All Fields] OR "posture"[All Fields] OR "force plate"[All Fields] OR "tug"[All Fields] OR "up and go"[All Fields] OR "bestest"[All Fields] OR "bbs"[All Fields] OR "berg balance scale"[All Fields] OR "kistler"[All Fields] OR "Dynamic Gait Index"[All Fields] OR "stability"[All Fields] OR "leg stance"[All Fields] OR "SLS"[All Fields] OR "Functional reach test"[All Fields] OR "FRT"[All Fields] OR "Fullerton Advance Balance Scale"[All Fields] OR "FABS"[All Fields] OR "Tinetti"[All Fields] OR "postural sway"[All Fields] OR "limb stance"[All Fields] OR "cop"[All Fields] OR "centre of pressure"[All Fields] OR "center of pressure"[All Fields]) AND ("Randomized clinical trial"[All Fields] OR "RCT"[All Fields] OR "random*"[All Fields] OR "Randomized controlled trial"[All Fields] OR "Randomised clinical trial"[All Fields] OR "randomised"[All Fields] OR "Randomised controlled trial"[All Fields]) | 408 |
| Embase (Elsevier) | Primary: April 2025 Supplementary: 31 Jan 2026 | (("aged" OR "older" OR "elder*" OR "senior"):ti,ab,kw AND ("virtual reality" OR "virtual reality exposure therapy" OR "exergaming" OR "telemedicine" OR "game-based rehabilitation" OR "head mounted display" OR "commercial video gam*" OR "video gam*" OR "Nintendo" OR "WiiPD" OR "WII fit" OR "Wii-hab" OR "Xbox" OR "PlayStation" OR "Kinect" OR "VR" OR "exergam*" OR "virtual reality exercis*" OR "active video gam*" OR "serious gam*" OR "augmented reality" OR "mixed reality" OR "Telehealth" OR "Telerehabilitation" OR "videoconferenc*" OR "Teletherapy" OR "teleconferenc*" OR "Telepractice" OR "teletreatment*" OR "Virtual conference" OR "ehealth" OR "artificial intelligence" OR "AI-assisted" OR "machine learning" OR "deep learning" OR "reinforcement learning" OR "intelligent tutoring" OR "adaptive feedback" OR "AI-based rehabilitation" OR "AI-powered" OR "robot-assisted" OR "intelligent rehabilitation" OR "smart rehabilitation" OR "sensor-based feedback" OR "personalized feedback" OR "automated feedback" OR "digital therapeutics" OR "conversational agent" OR "chatbot" OR "virtual agent" OR "virtual coach" OR "embodied agent"):ti,ab,kw AND ("gait" OR "postural balance" OR "gait analysis" OR "walk test" OR "postural stability" OR "balance" OR "posture" OR "force plate" OR "tug" OR "up and go" OR "bestest" OR "bbs" OR "berg balance scale" OR "kistler" OR "Dynamic Gait Index" OR "stability" OR "leg stance" OR "SLS" OR "Functional reach test" OR "FRT" OR "Fullerton Advance Balance Scale" OR "FABS" OR "Tinetti" OR "postural sway" OR "limb stance" OR "cop" OR "centre of pressure" OR "center of pressure"):ti,ab,kw AND ("randomized controlled trial" OR "Randomized clinical trial" OR "RCT" OR "random*" OR "Randomised clinical trial" OR "randomised"):ti,ab,kw) NOT ("stroke" OR "parkinson*" OR "Chronic Obstructive Pulmonary Disease" OR "COPD" OR "cardiac" OR "pulmonary" OR "neurologic" OR "hemiplegia" OR "paraplegia" OR "neuropathy" OR "arthroplasty" OR "kidney" OR "vestibular loss" OR "amblyopia" OR "fracture" OR "meniere*" OR "breast cancer" OR "cancer" OR "replacement" OR "alzheimer*" OR "dementia" OR "down syndrome" OR "low back pain" OR "amputation" OR "Huntington disease" OR "multiple sclerosis" OR "insomnia" OR "Fibromyalgia" OR "cochleovestibular loss" OR "osteoarthritis" OR "osteoporosis" OR "surgery" OR "Diabetes" OR "review" OR "meta-analysis" OR "study protocol" OR "quasi-randomized" OR "Cerebral palsy"):ti | 775 |
| Web of Science (Core Collection) | Primary: April 2025 Supplementary: 31 Jan 2026 | ((((ALL=("Aged" OR "older" OR "elder*" OR "senior")) AND ALL=("Virtual Reality" OR "Virtual Reality Exposure Therapy" OR "Exergaming" OR "game-based rehabilitation" OR "head mounted display" OR "commercial video gam*" OR "video gam*" OR "Virtual Reality" OR "Nintendo" OR "WiiPD" OR "WII fit" OR "Wii-hab" OR "Xbox" OR "PlayStation" OR "Kinect" OR "VR" OR "Exergam*" OR "Virtual Reality Exercis*" OR "Active Video Gam*" OR "Serious gam*" OR "augmented reality" OR "mixed reality" OR "Telemedicine" OR "Telemedicine" OR "Telehealth" OR "Telerehabilitation" OR "Videoconferenc*" OR "Teletherapy" OR "Teleconferenc*" OR "Telepractice" OR "Teletreatment*" OR "Virtual conference" OR "ehealth"[All Fields] OR "artificial intelligence"[All Fields] OR "AI-assisted"[All Fields] OR "machine learning"[All Fields] OR "deep learning"[All Fields] OR "reinforcement learning"[All Fields] OR "intelligent tutoring"[All Fields] OR "adaptive feedback"[All Fields] OR "AI-based rehabilitation"[All Fields] OR "AI-powered"[All Fields] OR "robot-assisted"[All Fields] OR "intelligent rehabilitation"[All Fields] OR "smart rehabilitation"[All Fields] OR "sensor-based feedback"[All Fields] OR "personalized feedback"[All Fields] OR "automated feedback"[All Fields] OR "digital therapeutics"[All Fields] OR "conversational agent"[All Fields] OR "chatbot"[All Fields] OR "virtual agent"[All Fields] OR "virtual coach"[All Fields] OR "embodied agent"[All Fields]))) AND ALL=("postural stability" OR "balance" OR "gait" OR "posture" OR "force plate" OR "tug" OR "up and go" OR "bestest" OR "bbs" OR "berg balance scale" OR "kistler" OR "Dynamic Gait Index" OR "stability" OR "leg stance" OR "SLS" OR "Functional reach test" OR "FRT" OR "Fullerton Advance Balance Scale" OR "FABS" OR "Tinetti" OR "postural sway" OR "limb stance" OR "cop" OR "centre of pressure" OR "center of pressure")) AND ALL=("Randomized clinical trial" OR "RCT" OR "random*" OR "Randomized controlled trial" OR "Randomized clinical trial" OR "RCT" OR "random*" OR "Randomized controlled trial" OR "Randomised clinical trial" OR "randomised" OR "Randomised controlled trial")) NOT TI=("stroke" OR "parkinson’s" OR "parkinson" OR "Chronic Obstructive Pulmonary Disease" OR "COPD" OR "cardiac" OR "pulmonary" OR "neurologic" OR "hemiplegia" OR "paraplegia" OR "neuropathy" OR "arthroplasty" OR "kidney" OR "vestibular loss" OR "amblyopia" OR "fracture" OR "Ménière's" OR "breast cancer" OR "cancer" OR "replacement" OR "Alzheimer's" OR "dementia" OR "down syndrome" OR "low back pain" OR "amputation" OR "Huntington disease" OR "multiple sclerosis" OR "insomnia" OR "Fibromyalgia" OR "cochleovestibular loss" OR "osteoarthritis" OR "osteoporosis" OR "surgery" OR "Diabetes" OR "review" OR "meta-analysis" OR "study protocol" OR "quasi-randomized" OR "Cerebral palsy") | 491 |
| Cochrane CENTRAL (Wiley) | Primary: April 2025 Supplementary: 31 Jan 2026 | aged OR elderly OR "older adult" OR senior* in Title Abstract Keyword NOT "stroke" OR "parkinson's" OR "parkinson" OR "Chronic Obstructive Pulmonary Disease" OR "COPD" OR "cardiac" OR "pulmonary" OR "neurologic" OR "hemiplegia" OR "paraplegia" OR "neuropathy" OR "arthroplasty" OR "kidney" OR "vestibular loss" OR "amblyopia" OR "fracture" OR "Ménière's" OR "breast cancer" OR "cancer" OR "replacement" OR "Alzheimer's" OR "dementia" OR "down syndrome" OR "low back pain" OR "amputation" OR "Huntington disease" OR "multiple sclerosis" OR "insomnia" OR "Fibromyalgia" OR "cochleovestibular loss" OR "osteoarthritis" OR "osteoporosis" OR "surgery" OR "Diabetes" OR "review" OR "meta-analysis" OR "study protocol" OR "quasi-randomized" OR "Cerebral palsy" in Title Abstract Keyword AND "virtual reality" OR "augmented reality" OR "mixed reality" OR exergam OR "active video gam" OR "serious gam" OR "video game" OR "game-based rehabilitation" OR "Wii fit" OR Nintendo OR Kinect OR Xbox OR PlayStation OR "telerehabilitation" OR telehealth OR telemedicine OR teletherapy OR videoconferenc* OR teleconferenc* OR "digital rehabilitation" OR "digital therapeutics" OR "artificial intelligence" OR "AI-assisted" OR "AI-based" OR "AI-powered" OR "robot-assisted" OR "intelligent rehabilitation" OR "adaptive feedback" OR "smart rehabilitation" in Title Abstract Keyword AND gait OR balance OR "postural control" OR "postural stability" OR "berg balance scale" OR BBS OR "timed up and go" OR TUG OR "gait speed" OR "walk test" OR "functional reach test" OR FRT OR "dynamic gait index" in Title Abstract Keyword - (Word variations have been searched) | 785 |
| **Total** |  |  | **2459** |

Record counts are the combined yield of the primary search (to April 2025) and the supplementary update search (to 31 January 2026), and correspond to the records identified per database in the PRISMA flow diagram (Figure 1). The Cochrane Central Register of Controlled Trials contains only trial records, so no randomized-design filter was applied to that strategy.

## **Table S2. Full-text articles excluded at the eligibility stage, by reason for exclusion**

Category totals are those of the PRISMA flow diagram (Figure 1) and sum to the 128 full-text articles excluded at the eligibility stage.

| **Reason for exclusion** | **Records, n** |
| --- | --- |
| Incorrect outcomes (no eligible balance or functional mobility outcome reported) | 62 |
| Incorrect intervention (not an eligible technology category, or robotic exoskeleton) | 26 |
| Incorrect study type (not a randomized controlled trial; conference abstract only) | 21 |
| Incorrect participants (mean age <60 years, or neurological, orthopedic or oncological comorbidity) | 19 |
| **Total** | **128** |

## **Table S3. Intervention classification rationale for every arm of every included trial**

| **#** | **Trial** | **Intervention arms as reported** | **Node assignment and rationale** |
| --- | --- | --- | --- |
| 1 | Toledano-Shubi et al. (2026) | G1: Otago-based exercise training via Zoom videoconferencing (telerehabilitation) G2: Face-to-face Otago-based exercise training (conventional exercises) | G1: Telerehabilitation G2: CE |
| 2 | Campelo et al. (2023) | G1: Exergame training (ET): Nintendo Wii-U (Wii Fit-U), including aerobic, strength, balance, and flexibility games G2: Conventional training (CT): aerobic, strength, balance, and flexibility exercises (seated, standing, floor mat) G3: No training (NT): maintain usual daily routine, no structured exercise | G1: Exergaming with Balance Platform G2: CE G3: NT |
| 3 | Parmak et al. (2025) | G1: Immersive VR (IVR): Oculus Meta Quest 2 HMD + FIT-XR application (BOX and SLAM modes); delivered under physiotherapist supervision at participants' homes G2: Active control group (ACG): physiotherapist-designed home-based traditional exercise program (15 exercises including balance, strengthening, flexibility, relaxation) | G1: Exergaming with Motion Capture G2: CE |
| 4 | Abd El-Kafy et al. (2024) | G1: C-Mill VR/AR treadmill training 30 min (VR/AR interactive games, 4 sets, progressive difficulty, obstacle avoidance, stepping targets, virtual environment walking) + Conventional exercise 30 min (balance board/perturbation training 20 min + indoor gait training 10 min) G2: Same C-Mill treadmill but without VR/AR games or visual projections, regular walking 30 min + identical conventional exercise | G1: Other Serious Games G2: CE |
| 5 | Guede-Rojas et al. (2023) | G1: Conventional physical therapy 60 min + Kinemotion platform exergames 30 min G2: Same conventional physical therapy | G1: Exergaming with Motion Capture G2: CE |
| 6 | Lee (2023) | G1: Home-based exergame using Nintendo Switch Ring Fit Adventure G2: Online education on fall prevention and musculoskeletal health management | G1: Other Serious Games G2: NT |
| 7 | Sheehy et al. (2025) | G1: intronix non-immersive VR software + Kinect v2 camera (Microsoft), Customized gamified exercises: balance, stepping, strengthening, aerobic conditioning, range of motion, coordination, cognition/memory; parameters remotely adjusted by Research Therapist G2: Usual activity (no exercise intervention) | G1: Exergaming with Motion Capture G2: NT |
| 8 | Wang (2023) | G1: Semi-immersive VR exercise (Uniigym Interactive Somatosensory Fitness system); 3 large laser projectors creating a wrap-around VR environment; content: yoga/Tai Chi warm-up/cool-down (15-20 min) + boxing and aerobic dance main exercises (55-60 min); targeting flexibility, strength, cardiorespiratory fitness, balance, and agility G2: No intervention (maintain usual daily activities) | G1: Other Serious Games G2: NT |
| 9 | Ghous et al. (2024) | G1 (VRTG): Non-immersive VR training using Nintendo Wii Fit Plus software + Wii Balance Board (Bluetooth); LED screen displays Avatar reflecting patient's COG; games include skiing slalom, heading, tightrope walking, penguin slide, balance slalom, jogging (7 programs); difficulty adjusted per participant ability; participant stands 2m from screen; physiotherapist supervision throughout G2 (TOCTG): Task-oriented circuit training; 10 exercise stations: sit-to-stand, self-sways, standing balance, step-ups, standing hamstring curls, tandem walking, Swiss ball squats, backward walks, side leg lifts, marching in place; progressive intensity and repetition increase | G1: Exergaming with Balance Platform G2: CE |
| 10 | Elmas (2024) | G1: Calisthenic exercises performed online via WhatsApp video conference with a physiotherapist providing real-time feedback and encouragement; pre-recorded exercise videos sent in advance for familiarization G2: Same calisthenic exercises performed at home using self-administered pre-recorded video guide; weekly phone check for compliance; no real-time therapist interaction | G1: Telerehabilitation G2: CE |
| 11 | Park (2025) | G1: AR-based exercise on DIDIM platform with LiDAR sensors; floor-projected visual cues; 10 structured exercises (stretching, tap step, on-spot running, diagonal step, cross back step, shuttle run, catching bugs, rhythm dance, image memory game, color word matching); shuttle run introduced from week 4 G2: Usual daily activities; no new structured exercise program | G1: Other Serious Games G2: NT |
| 12 | Gatica-Rojas (2025) | G1: Telerehabilitation exergame: Nintendo Wii + Wii Balance Board via synchronous videoconference; remote physiotherapist supervision; peer-monitor (tactile + verbal guidance weeks 1-3, verbal only weeks 4-6); games: Snowboard, Penguin Slide, Super Hula Hoop, Yoga G2: Face-to-face exergame: identical games and parameters; conducted at Human Motor Control Laboratory, Universidad de Talca; direct physiotherapist supervision | G1: Telerehabilitation G2: Exergaming with Balance Platform |
| 13 | Chan et al. (2024) | G1: Nintendo Ring Fit Adventure (NRFA)-based exercise program. Content: yoga, lower limb strengthening, balance, and rhythmic exergames. Three progressive stages (easy/medium/hard) with increasing repetitions and variations. Stage 3 included standing on a balance board for selected exercises. Supervised by a trained exercise instructor throughout with verbal feedback, manual support, and intensity adjustment G2: Usual care. Participants received advice from the exercise instructor on how to improve physical performance and continued to receive care from the community center and other healthcare providers for 8 weeks; no structured exercise program | G1: Other Serious Games G2: NT |
| 14 | Rendon et al. (2012) | G1: Nintendo® Wii Fit + Wii Balance Board (WBB); 3 balance games (lunges, single leg extensions, twists); 3×/week × 6 weeks = 18 sessions; 35-45 min/session; supervised by physical therapist; assistive device available; 8-min warm-up + 8-min cool-down on stationary bicycle or recumbent stepper G2: Pre- and post-test measurements only; no intervention; instructed to maintain usual daily activities | G1: Exergaming with Balance Platform G2: NT |
| 15 | Ku et al. (2019) | G1: 3D Interactive Augmented Reality System (3D-ARS) training. Uses a Kinect sensor to track whole-body motion and interact with virtual objects on a large screen. Includes three tasks: Balloon game (hip), Cave game (knee), and Rhythm game (single-leg stance) G2: Conventional physical fitness program (lower-extremity strengthening, balance, and endurance training) | G1: Exergaming with Motion Capture G2: CE |
| 16 | Liepa et al. (2022) | G1: Immersive VR exergame using HTC VIVE headset + chest-mounted sensor (tracking trunk movement) + balance board (Togu Balanza Ballstep), playing "Falling diamonds" game. 20 min gameplay + 5 min warm-up, 2x/week for 9 weeks G2: Non-immersive exergame using same chest sensor + balance board, but played on a laptop screen without VR headset G3: No intervention, maintained usual daily activities | G1: Other Serious Games G2: Other Serious Games G3: NT |
| 17 | Pluchino et al. (2012) | G1: Balance training using Wii Balance Board with 8 games (soccer heading, ski slalom, ski jump, tightrope walk, penguin slide, etc.). Difficulty adjusted based on game scores G2: Sun-style Tai Chi, 12 movements, taught by a certified instructor. G3: 14 functional balance activities (standing on compliant surfaces, walking over obstacles, single-leg stance, etc.) | G1: Exergaming with Balance Platform G2: CE G3: CE |
| 18 | Schättin et al. (2016) | G1: Cognitive-motor training using a pressure-sensitive dance platform (Impact Dance Platform, 87.5×87.5cm) with foot pushes on arrows (right, left, up, down) to interact with video games. Games: Balloon, Step, Season, Space. 30 min/session (5 min warm-up + 20 min gameplay + 5 min cool-down), 3x/week for 24 sessions (8-10 weeks). Difficulty individually adjusted G2: Conventional balance training with repetitive static and dynamic exercises on stable and unstable surfaces. 30 min/session, 3x/week for 24 sessions. Progression through changes in base of support, eye closure, external perturbations, etc | G1: Exergaming with Balance Platform G2: CE |
| 19 | Karahan et al. (2015) | G1: Xbox 360 Kinect™ console with exergames (Kinect Adventures, Kinect Sports, Kinect Sports Season Two), including soccer, tennis, table tennis, skiing, golf, volleyball, bowling simulations. Supervised by a nurse G2: Home-based balance exercise including stretching (hamstring, quadriceps, pelvic girdle, pectoral muscles), strengthening (quadriceps, hamstring, biceps, abdominal muscles), standing on two feet, semi-tandem stance, single-leg stance, heel standing, toe standing | G1: Exergaming with Motion Capture G2: CE |
| 20 | Kwok & Pua (2016) | G1: Supervised 1-hour session weekly (20 min WiiActive gaming + resistance band) + home exercises (≥2 days/week). 12 weeks total (12 sessions). Used Wii Balance Board G2: Supervised 1-hour session weekly (stationary bike, treadmill, elliptical, free weights, balance discs, gym ball) + same home exercises. 12 weeks | G1: Exergaming with Balance Platform G2: CE |
| 21 | De Lima et al. (2021) | G1: Xbox Kinect console with game “Your Shape Fitness evolved”, including Zen (stretching/balance/flexibility), Pump it, Wall Breaker (boxing-like), Kick it (soccer), Hurricane, Stack in Up (balance) G2: No intervention, maintained usual daily activities, instructed not to change lifestyle | G1: Exergaming with Motion Capture G2: NT |
| 22 | Whyatt et al. (2015) | G1: Custom-made balance games using Wii Balance Board (WBB), including Apple Catch (mediolateral COP control), Bubble Pop, Avoid the Shark, Smart Shrimp (multidimensional COP control + cognitive tasks) G2: Recorded daily activity (activity diary), no structured exercise | G1: Exergaming with Balance Platform G2: NT |
| 23 | Sonthikul et al. (2022) | G1: Active video gaming using a movable balance platform (Sensamove Miniboard), controlling a ball on screen through weight shifting G2: Core stabilization exercise (abdominal hollowing, bridging, lower trunk rotation, plank, etc.).Supervised by physiotherapist, home exercises monitored by phone and logbook | G1: Exergaming with Balance Platform G2: CE |
| 24 | Zhao et al. (2022) | G1: Active video games using Nintendo Switch + Joy-Con controllers, including: (1) Zumba Fitness (dance game); (2) Fitboxing2 (boxing virtual game); (3) Mario Tennis Ace (tennis game). Designed based on Self-Determination Theory (SDT) emphasizing competence, autonomy, and relatedness G2: No intervention, continued usual daily activities | G1: Other Serious Games G2: NT |
| 25 | Yang et al. (2020) | G1: Exergame training using Xbox Kinect with game “Your Shape: Fitness Evolved II”, including Zen Energy, Yoga, Destination Bollywood, Cardio Boxing, Humana Vatility, Cardio G2: Balance training program based on Campbell et al. (1997), including lower-body strengthening (squats, leg lifts), tandem standing, tandem walking, single-leg stance, etc | G1: Exergaming with Motion Capture G2: CE |
| 26 | Toulotte et al. (2012) | G1: Adapted Physical Activities including step length/height exercises, cervical spine mobility, ocular mobility, strength, proprioception, flexibility, static/dynamic balance G2: Nintendo Wii Fit console training with games including soccer heading, ski jump, yoga, downhill skiing, balance balls, tightrope walk G3: 30 min APA + 30 min Wii Fit G4: No training, watched television, played board games | G1: CE G2: Exergaming with Balance Platform G3: Exergaming with WBP+CE G4: NT |
| 27 | Gallardo-Meza et al. (2022) | G1: Nintendo Wii Fit Plus with Wii Balance Board. Games: (1) Sky Slalom (10 min); (2) Sky Jump (10 min); (3) Balance Bubble (10 min). 2 sessions/week for 4 weeks (8 sessions) G2: No additional intervention, maintained usual daily activities | G1: Exergaming with Balance Platform G2: NT |
| 28 | Singh et al. (2013) | G1: Balance-focused interactive virtual-reality games using Nintendo Wii Balance Board, including Ski Slalom, Table Tilt, Penguin Slide, Soccer Heading, Tight Rope Walk, Perfect 10, Tilt City G2: Therapeutic balance exercises, including one-leg standing, free leg swinging, moving objects forward/backward/sideways, walking in place/sideways/backwards | G1: Exergaming with Balance Platform G2: CE |
| 29 | Wu et al. (2010) | G1: Telecommunication-based Tai Chi exercise. Participants exercised at home connected to instructor via custom video conferencing device (DocBox). 24-form Yang-style Tai Chi G2: Community center-based Tai Chi exercise. Participants exercised at YMCA with live instructor G3: Home video-based Tai Chi exercise. Participants exercised at home using a custom DVD set (45 one-hour sessions). No real-time interaction | G1: Telerehabilitation G2: CE G3: NT |
| 30 | Pichierri et al. (2012) | G1: 2 sessions/week for 12 weeks (24 sessions). ~40 min/session including warm-up (5 min), progressive resistance training (25 min, core and lower extremities, using weight vests), balance exercises (10 min, using air-filled cushions and grip balls). Additionally, 10-15 min of dance video game (StepMania on metal dance pad) after each session, following on-screen arrows. G2: Same strength and balance training (content, frequency, duration as dance group), without dance video game | G1: Exergaming with Balance Platform G2: CE |
| 31 | Hou & Li (2022) | G1: Dance mat version of Stepmania game. Participants stepped on the dance mat following on-screen arrows (forward, backward, left, right). 3 sessions/week, 30 min gameplay + 5 min warm-up/cool-down for 12 weeks (36 sessions). 18 songs, 4 difficulty levels. G2: Computer keyboard version of Stepmania game. Participants pressed keyboard keys with fingers following the same game content. 3 sessions/week, 30 min gameplay + 5 min warm-up/cool-down for 12 weeks (36 sessions). G3 : No intervention, maintained usual daily activities. | G1: Exergaming with Balance Platform G2: Other Serious Games G3: NT |
| 32 | Sadeghi et al. (2021) | G1: Traditional balance training (single-leg stance, heel/toe standing, tandem stance, walking forward/backward, weight shifting). 3 sessions/week, 40 min/session (5 min warm-up + 30 min training + 5 min cool-down) for 8 weeks. Progressive intensity. G2: Xbox Kinect virtual reality training with games Light Race, Target Kick, Goalkeeper. 3 sessions/week, 40 min/session for 8 weeks. Progressive difficulty. G3 : 15 min BT + 15 min VR + 5 min warm-up/cool-down, 3 sessions/week for 8 weeks. G4: Waitlist control, continued usual daily activities, offered training after study. | G1: CE G2: Exergaming with Motion Capture G3: CE +Exergaming with Motion Capture G4: NT |
| 33 | Bacha et al. (2018) | G1: Xbox Kinect Adventures games (Space Pop, 20,000 Leaks, Reflex Ridge, River Rush). 1 h/session, 2 sessions/week for 14 sessions. Full-body motion interaction with visual/auditory feedback. G2: Conventional physical therapy including warm-up, static/dynamic balance, endurance, muscle strength, coordination, stretching, cool-down. 1 h/session, 2 sessions/week for 14 sessions. | G1: Exergaming with Motion Capture G2: CE |
| 34 | Franco et al. (2012) | G1: Balance games on Wii Balance Board (soccer heading, ski jump, ski slalom, tightrope, table tilt, balance bubble). 2 individual sessions/week ×10-15 min + daily home exercises (15 min) for 3 weeks (6 sessions). G2: Matter of Balance exercise component (strength + balance). Group sessions, 2 sessions/week ×30-45 min for 3 weeks (6 sessions). G3 : No intervention, maintained usual daily activities. | G1: Exergaming with Balance Platform G2: CE G3: NT |
| 35 | Lim et al. (2017) | G1: Complex VR training using Wii-Fit Plus (balance + strength + flexibility + aerobic). 10 sessions (2/week, 60 min) over 5 weeks. G2: Balance-only VR training using same games (6 balance games). Same frequency and duration. | G1: Exergaming with Balance Platform G2: Exergaming with Balance Platform |
| 36 | Park & Yim (2016) | G1: Conventional exercise (30 min, side stepping, tandem walking, backward walking, one-leg standing, etc.) + 3D virtual reality kayak program (20 min, using real paddle, 3D projection of river/lake). 2 sessions/week for 6 weeks (12 sessions). G2: Conventional exercise only (30 min, same content as experimental group). 2 sessions/week for 6 weeks. | G1: Other Serious Games G2: CE |
| 37 | Padala et al. (2017) | G1: Wii-Fit balance board training including yoga, strength, aerobics, balance games, Training Plus. 45 min/session, 3 sessions/week for 8 weeks (24 sessions). Self-selected games with encouragement to progress. G2: Computer-based cognitive training (Brain-Fitness program) covering memory, language, attention, executive function, visuospatial domains. Same frequency and duration, no exercise component. | G1: Exergaming with Balance Platform G2: NT |
| 38 | Bieryla & Dold (2013) | G1: Wii Fit training (yoga: half moon, chair, warrior; aerobic: torso twists; balance games: soccer heading, ski jump). 30 min/session, 3 sessions/week for 3 weeks (9 sessions). One-on-one supervision, no physical assistance, chairs for safety. G2: Continue normal daily activities, no intervention | G1: Exergaming with Balance Platform G2: NT |
| 39 | Bieryla (2016) | G1: Xbox Kinect training including Your Shape: Fitness Evolved (Zen Sessions, 15 min yoga/tai chi) and Kinect Adventures (20,000 leaks, rally ball, reflex ridge, 15 min). 30 min/session, 3 sessions/week for 3 weeks (9 sessions). One-on-one supervision, verbal instructions, no physical assistance. G2: Normal daily activities, no intervention | G1: Exergaming with Motion Capture G2: NT |
| 40 | Sato et al. (2015) | G1: Exergame using Kinect sensor, including apple game (reaching), tightrope standing game (tandem stance reaching), balloon popping game (squatting), one-leg standing game (knee lifting). 1-2 sessions/week for 24 sessions, 40-60 min/session. G2: No intervention, usual daily activities | G1: Exergaming with Motion Capture G2: NT |
| 41 | Delbaere et al. (2021) | G1: StandingTall telerehabilitation balance training delivered via tablet computer at home. Includes standing balance, targeted stepping, and step-up exercises. Minimum 2 hours/week for 2 years. Two home visits in first month and incidental phone calls in first 6 months, then unsupervised. G2: Health education (weekly fact sheets) delivered via tablet, no exercise component. Same frequency of contact. | G1: Telerehabilitation G2: NT |
| 42 | Phirom et al. (2020) | G1: Xbox Kinect exergames (Fruits Hunter, Where am I?, Whack a mole, Sky fall, Crossing poison river) simultaneously training physical (stepping, balance) and cognitive functions. 3 sessions/week, 60 min/session for 12 weeks (36 sessions). G2: Educational material (fall prevention and cognitive enhancement) + weekly phone call, no exercise. | G1: Exergaming with Motion Capture G2: NT |
| 43 | Lee (2021) | G1: Virtual reality gait training using smartphone app (BitGym) providing video flow synchronized with walking speed, on a non-motorized (self-powered) treadmill. 5 sessions/week, 50 min/session (10 min warm-up + 30 min main + 10 min cool-down) for 4 weeks (20 sessions). G2: Conventional treadmill training using the same non-motorized treadmill, no virtual reality video. Same frequency and duration. | G1: Other Serious Games G2: CE |
| 44 | Adcock et al. (2020) | G1: In-home multicomponent exergame including Tai Chi-inspired exercises, dancing, and step-based cognitive games. Uses IMU sensors (wrists and ankles) for motion capture. 3 sessions/week, 30-40 min/session for 16 weeks (48 sessions). Progressive difficulty. G2: Usual care, continued normal daily living | G1: Exergaming with Motion Capture G2: NT |
| 45 | Morat et al. (2019) | G1: Volitional stepping exergames under unstable conditions (Dividat Senso platform suspended on springs). 11 stepping games training cognitive and motor abilities. 3 sessions/week, 40 min/session (10-12 min net gaming) for 8 weeks (24 sessions). G2: Volitional stepping exergames under stable conditions (fixed platform). Same games and training parameters. G3 : No intervention, maintained usual daily activities. | G1: Exergaming with Balance Platform G2: Exergaming with Balance Platform G3: NT |
| 46 | Benitez-Lugo et al. (2022) | G1: Nintendo Wii Balance Board games “Penguin Slide” (weight shifting) and “Step Plus” (stepping up/down). 2 sessions/week, 30 min/session for 8 weeks (16 sessions). Also received usual care (memory and joint mobility workshops). G2: Usual care only (memory and joint mobility workshops), no additional exercise intervention. | G1: Exergaming with Balance Platform G2: NT |
| 47 | Jorgensen et al. (2013) | G1: Nintendo Wii Balance Board training including 5 balance games and 1 strengthening exercise (standing rowing squat). 2 sessions/week, ~35 min net training for 10 weeks (20 sessions). Supervised by physiotherapist. G2: Daily use of EVA shoe insoles, told to improve balance (placebo control). No exercise intervention. | G1: Exergaming with Balance Platform G2: NT |
| 48 | Schwenk et al. (2014) | G1: Balance training using wearable sensors (LegSys, 5 inertial sensors) with interactive game-based interface. Includes: (1) ankle point-to-point reaching tasks (weight shifting, AP/ML directions, with visuomotor rotation); (2) virtual obstacle crossing tasks (alternating leg lifting to cross virtual boulders). 2 sessions/week, 45 min/session for 4 weeks (8 sessions). Real-time visual/auditory feedback from sensors. G2: No intervention, maintained usual daily activities | G1: Interventions with Wearables G2: NT |
| 49 | Lee et al. (2015) | G1: Xbox Kinect + “Zen Energy” game (Tai Chi-style movements). Real-time visual and auditory feedback with 3D avatar. 3 sessions/week, 60 min/session for 8 weeks (24 sessions). G2: Group-based exercise (postural, balance, functional, lower body coordination and strength training) with auditory feedback from physiotherapist. Same frequency and duration. | G1: Exergaming with Motion Capture G2: CE |
| 50 | Fakhro et al. (2020) | G1: Nintendo Wii Fit balance board training, games: first 4 weeks “Soccer Heading”, last 4 weeks “Table Tilt”. 40 min/session (5 min warm-up + 30 min gaming + 5 min cool-down), 3 sessions/week for 8 weeks (24 sessions). G2: No intervention, maintained usual daily activities | G1: Exergaming with Balance Platform G2: CE |
| 51 | Moreira et al. (2021) | G1: Xbox 360 (Microsoft) with Kinect motion capture, using "Your Shape™: Fitness Evolved." Structure: 5 min warm-up (global movements), 25 min strength training (squats and lunges, 4-6 sets × 8-16 reps), 15 min balance and cardiorespiratory training (boxing, lateral and anteroposterior displacements), 5 min cool-down (Tai Chi Chuan slow-pace). Progression determined by in-game difficulty levels. G2: Traditional multicomponent exercise: knee/hip flexion-extension, hip adduction-abduction, internal/external rotation, ankle dorsi-plantar flexion (2-3 sets × 8-12 reps, 60 s rest between sets), using ankle cuff weights 1-4 kg (initial increment ~3% body mass); balance exercises: single-leg stance, semi-tandem, tandem, forward/backward/lateral displacements, plantar/dorsiflexion walking. | G1: Exergaming with Motion Capture G2: CE |
| 52 | Chen (2020) | G1: AR-assisted selected Tai-Chi (sTC): Microsoft Kinect motion capture sensor used to detect full-body movements and construct digital skeleton. Skeletal data compared in real-time with Tai-Chi master's movements, providing visual feedback. 8 simplified movements extracted from 24-form Yang-style Tai-Chi, individually assigned based on each participant's maximum COP displacement and endurance; progressive in difficulty G2: Traditional Tai-Chi (tTC): Complete 24-form Yang-style Tai-Chi under instruction of a certified Tai-Chi master; typically 2-3 complete sequences per session | G1: Exergaming with Motion Capture G2: CE |
| 53 | Khanmohammadi (2021) | G1: Nintendo Wii Fit balance board (WBB) training; 6 balance games (2 static: single leg extension, torso twist; 4 dynamic: table tilt, penguin fishing, soccer heading, tightrope walk); games randomly selected each session, ~10 min per game; individualized progression across 3 difficulty levels G2: Motor-cognitive dual-task training with variable-priority instruction: simultaneous motor tasks (static/dynamic balance, walking, sit-to-stand, etc.) and cognitive tasks (verbal fluency, backward counting), alternating focus between motor and cognitive priority | G1: Exergaming with Balance Platform G2: CE |
| 54 | Yousefi Babadi & Daneshmandi (2021) | G1: Virtual reality training (VRT) using Xbox Kinect (infrared camera motion-capture sensor). Games from Sports Pack 1 (boxing, table tennis, soccer) and Sports Pack 2 (golf, ski, American football); all performed standing, requiring upper and lower extremity use. Participant placed 1.5-2 m from sensor; real-time visual feedback provided on monitor G2: Conventional balance training (CBT) based on Hirase et al. (2015): 10 standing exercises in 3 progressive phases. Weeks 1-3: double-stance standing, heel/toe raises, walking in place; Weeks 4-6: adding neck hyperextension, free-leg swinging, trunk rotation; Weeks 7-9: adding floor touch, sideways walking, one-leg standing. Each session: 10 min warm-up + 40 min training + 10 min cool-down G3 : No treatment control (NT): maintained routine daily activities, no specific exercise | G1: Exergaming with Motion Capture G2: CE G3: NT |
| 55 | Kim & Cho (2022) | G1: Nintendo Wii Fit program (Wii Balance Board + remote); games: Balance ski, Table tile (weight transfer/support/speed), Jogging, Rhythm step (aerobic/muscular endurance/strength); 5 min warm-up + 5 min per game + 5 min rest G2: Motor Imagery Training (MIT); conducted at 18:00 in quiet, dimly lit room; seated; guided by auditory cues; motor-sensory imagery (foot pressure, weight-shift sensation) + visual-sensory imagery (3rd-person perspective); 150s relaxation + 15 min main training G3 : No treatment (NT); no intervention | G1: Exergaming with Balance Platform G2: CE G3: NT |
| 56 | Bao (2018) | G1: In-home balance training with vibrotactile sensory augmentation (SA) via smartphone balance trainer. Each session: 6 exercise categories (static standing, compliant surface standing, weight shifting, modified COG, gait), 1-2 exercises per category, 6 reps each for 30 s. Vibrotactile SA provided during 4 of 6 repetitions per exercise (67% feedback). Device: two Apple iPod Touch units (sensing + user interface) + elastic belt + tactor bud with 4 vibration motors worn at L4/L5 G2: Same in-home balance training protocol; wore identical device but received NO vibrotactile SA | G1: Interventions with Wearables G2: CE |
| 57 | Bao et al. (2022) | G1: 8-week home-based balance training (24 sessions) with vibrotactile sensory augmentation (SA): smartphone-based balance trainer (two iPods); SA device mounted at L4/L5; vibrotactile cues delivered to navel, spine, and bilateral sides when trunk motion exceeded thresholds; instruction: "move away from the vibration"; exercise types: static standing (firm/compliant surfaces), weight shifting, modified center of gravity (arm raises), gait (no SA during gait) G2: Identical 8-week home-based balance training (24 sessions) using same device but without vibrotactile SA activation | G1: Interventions with Wearables G2: CE |
| 58 | Yerlikaya (2021) | G1: Interactive Telerehabilitation Home Exercise (ITHE): participants exercised with a physiotherapist via video platforms (WhatsApp, Google Meet) in groups of 2-3; program included strengthening and balance exercises; 1-2 familiarization sessions conducted prior G2: Non-supervised Home Exercise (NHE): first session supervised by physiotherapist in person; subsequent sessions performed independently at home using instructional videos; therapist followed up via phone; same exercise content as ITHE G3 : no exercise intervention during the study period; exercise training and videos provided after study completion | G1: Telerehabilitation G2: CE G3: NT |
| 59 | Gschwind et al (2015a) | G1: Microsoft Kinect® (3D depth sensor), 3 balance exergames (walking, stepping, weight-shifting based on WEBB) + 5 strength exercises (knee extension/flexion, hip abduction, calf/toe raises based on Otago Exercise Program), Movements recorded by Kinect and displayed as avatar on screen, Unsupervised, home-based, Balance exergames: 120 min/week; Strength exercises: 60 min/week, Balance exergames: 120 min/week; Strength exercises: 60 min/week G2: Pressure-sensitive electronic step mat (6-direction arrows), StepMania (divided attention, inhibition, processing speed); Stepper (processing speed, selective attention); Trail-stepping (visual attention, set-shifting); Tetris-stepping (visuospatial, problem-solving), Real-time stepping feedback via on-screen arrow cues, Unsupervised, home-based, Minimum 3 sessions × 20 min/week, including all 4 games, Selection of higher difficulty levels with greater cognitive load and stepping speed G3: Educational booklet on evidence-based health and fall prevention advice; asked to maintain usual activities | G1: Exergaming with Motion Capture G2: Exergaming with Balance Platform G3: NT |
| 60 | Gschwind et al (2015b) | G1: 16-week iStoppFalls ICT system: ① Balance exergames (Kinect motion capture): 3 games (Bumble Bee Park, Hills & Skills, Balance Bistro); recommended ≥3 sessions/week × ~40 min; progressive dual-task cognitive elements added at higher levels; ② Lower limb strength training (Otago-based): recommended ≥3 sessions/week × 15-20 min, 2-3 sets × 10-15 reps, progressive load with ankle cuff weights (1-3 kg); ③ Tablet-based reminders and automated fall risk monitoring; ④ Educational booklet G2: Educational booklet (healthy lifestyle and fall prevention advice) + maintenance of habitual exercise routines; no additional intervention | G1: Other Serious Games G2: NT |

Arms were assigned independently by 2 reviewers, with disagreements resolved by discussion and, where necessary, by a third reviewer. Where an intervention showed features of more than 1 category, assignment followed the dominant source of augmented feedback. Node abbreviations are defined in the footnote to Table 2 of the main text. Trial numbers correspond to the order of citation in the main text (references 28-87).

## **Table S4. Characteristics of the 60 included trials**

| **#** | **Trial** | **Population** | **Intervention arms** | **Duration / frequency / total sessions / session length** | **Outcomes extracted** | **Dropouts** |
| --- | --- | --- | --- | --- | --- | --- |
| 1 | Toledano-Shubi et al. (2026) | Community-dwelling older adults n=81 age: 73.2±4.0 | G1: Otago-based exercise training via Zoom videoconferencing (telerehabilitation) G2: Face-to-face Otago-based exercise training (conventional exercises) | 6 months/2 sessions a week/48 sessions/45-60 minutes | TUG, BBS, MiniBESTest, One Leg Stance, 4MWT, 30s STS, Fear of Falling (MFES), QOL (SF-12) | G1 (Tele): 5 G2 (FTF): 6 |
| 2 | Campelo et al. (2023) | Community-dwelling older adults n=40 age:72±7 | G1: Exergame training (ET): Nintendo Wii-U (Wii Fit-U), including aerobic, strength, balance, and flexibility games G2: Conventional training (CT): aerobic, strength, balance, and flexibility exercises (seated, standing, floor mat) G3: No training (NT): maintain usual daily routine, no structured exercise | 6 weeks/3 sessions a week/18 sessions/40-50minutes | TUG, ECS (exercise confidence), K&UQ (PA knowledge), MPAM-R (motivation for PA), daily step count (Fitbit Flex 2) | G1 (ET): 1 G2 (CT): 2 G3 (NT):1 |
| 3 | Parmak et al. (2025) | Community-dwelling older adults n=44 age:75.36±4.83 | G1: Immersive VR (IVR): Oculus Meta Quest 2 HMD + FIT-XR application (BOX and SLAM modes); delivered under physiotherapist supervision at participants' homes G2: Active control group (ACG): physiotherapist-designed home-based traditional exercise program (15 exercises including balance, strengthening, flexibility, relaxation) | 8 weeks/3 sessions a week/24 sessions/35 minutes | Physical fitness (SFT subtests), fall risk (MFS), fear of falling (FES-I), fatigue (FACIT), quality of life (WHOQOL-OLD), treatment satisfaction (VAS), VR sickness (VRSQ) | 0 |
| 4 | Abd El-Kafy et al. (2024) | Community-dwelling older adults n=60 age:66.53±3.82 | G1: C-Mill VR/AR treadmill training 30 min (VR/AR interactive games, 4 sets, progressive difficulty, obstacle avoidance, stepping targets, virtual environment walking) + Conventional exercise 30 min (balance board/perturbation training 20 min + indoor gait training 10 min) G2: Same C-Mill treadmill but without VR/AR games or visual projections, regular walking 30 min + identical conventional exercise | 6 weeks/3 sessions a week/18 sessions/60 minutes | TUG, Fall Risk OSI (FR test, %); LOS OSI (%); Time to complete LOS (s) | G1 (Other serious games): 1 G2 (ACE): 2 |
| 5 | Guede-Rojas et al. (2023) | Community-dwelling older adults n=50 age:71.8±6.8 | G1: Conventional physical therapy 60 min + Kinemotion platform exergames 30 min G2: Same conventional physical therapy | 8 weeks/2 sessions a week/16 sessions/90 minutes | 30s STS, 8-Foot Up-and-Go(seconds), 30-s arm curl, 2-min step test, chair sit-and-reach; back scratch | 0 |
| 6 | Lee (2023) | Community-dwelling older adults n=60 age:80.39±2.57 | G1: Home-based exergame using Nintendo Switch Ring Fit Adventure G2: Online education on fall prevention and musculoskeletal health management | 8 weeks/3 sessions a week/24 sessions/50 minutes | TUG, BBS, FRT, 30s STS, FTSTS, Fear of Falling (MFES) | G1: Other interventions 2 G2: No treatment (NT) 1 |
| 7 | Sheehy et al. (2025) | Community-dwelling Older adults n=16 age:75.1±6.0 | G1: intronix non-immersive VR software + Kinect v2 camera (Microsoft), Customized gamified exercises: balance, stepping, strengthening, aerobic conditioning, range of motion, coordination, cognition/memory; parameters remotely adjusted by Research Therapist G2: Usual activity (no exercise intervention) | 8 weeks/3-5 sessions a week/24-40 sessions/20-30 minutes | TUG, BBS, FRT, 30s STS | G1: 1 |
| 8 | Wang (2023) | Healthy older adults n = 98 age:72.16 ± 4.9 | G1: Semi-immersive VR exercise (Uniigym Interactive Somatosensory Fitness system); 3 large laser projectors creating a wrap-around VR environment; content: yoga/Tai Chi warm-up/cool-down (15-20 min) + boxing and aerobic dance main exercises (55-60 min); targeting flexibility, strength, cardiorespiratory fitness, balance, and agility G2: No intervention (maintain usual daily activities) | 12 weeks/2 sessions a week/24 sessions/75-90 minutes | 30s STS, Back Scratch Test, Chair Sit-and-Reach Test, Arm Curl Test, Chair-Stand Test, 2-Minute Step Test, 8-ft Up-and-Go Test | G1: Other Serious Games 4 G2: No treatment (NT) 3 |
| 9 | Ghous et al. (2024) | Community-dwelling Older adults n=28 age:66.91.1±3.79 | G1 (VRTG): Non-immersive VR training using Nintendo Wii Fit Plus software + Wii Balance Board (Bluetooth); LED screen displays Avatar reflecting patient's COG; games include skiing slalom, heading, tightrope walking, penguin slide, balance slalom, jogging (7 programs); difficulty adjusted per participant ability; participant stands 2m from screen; physiotherapist supervision throughout G2 (TOCTG): Task-oriented circuit training; 10 exercise stations: sit-to-stand, self-sways, standing balance, step-ups, standing hamstring curls, tandem walking, Swiss ball squats, backward walks, side leg lifts, marching in place; progressive intensity and repetition increase | 8 weeks/3 sessions a week/24 sessions/30 minutes | TUG, BBS(only baseline),SF-12 (PCS + MCS), MoCA (0-30, ICC=0.99) | 0 |
| 10 | Elmas (2024) | Healthy older adults n = 30 age:68.8 ± 3.57 | G1: Calisthenic exercises performed online via WhatsApp video conference with a physiotherapist providing real-time feedback and encouragement; pre-recorded exercise videos sent in advance for familiarization G2: Same calisthenic exercises performed at home using self-administered pre-recorded video guide; weekly phone check for compliance; no real-time therapist interaction | 6 weeks/3 sessions a week/18 sessions/25-40 minutes | TUG, SLS, Chair sit-stand 30s, 6-minute walk test (m) | G1: Telerehabilitation 3 G2: Conventional exercises (CE)2 |
| 11 | Park (2025) | Community-dwelling Older adults n=40 age:71.25.1±3.86 | G1: AR-based exercise on DIDIM platform with LiDAR sensors; floor-projected visual cues; 10 structured exercises (stretching, tap step, on-spot running, diagonal step, cross back step, shuttle run, catching bugs, rhythm dance, image memory game, color word matching); shuttle run introduced from week 4 G2: Usual daily activities; no new structured exercise program | 6 weeks/3 sessions a week/18 sessions/30 minutes | TUG, 1-Minute Sit-to-Stand Test, 30s STS, Trail Making Test A/B, FVC/FEV1, MIP/MEP | 0 |
| 12 | Gatica-Rojas (2025) | Community-dwelling Older adults n=16 age:69.3±3.1 | G1: Telerehabilitation exergame: Nintendo Wii + Wii Balance Board via synchronous videoconference; remote physiotherapist supervision; peer-monitor (tactile + verbal guidance weeks 1-3, verbal only weeks 4-6); games: Snowboard, Penguin Slide, Super Hula Hoop, Yoga G2: Face-to-face exergame: identical games and parameters; conducted at Human Motor Control Laboratory, Universidad de Talca; direct physiotherapist supervision | 6 weeks/3 sessions a week/18 sessions/25 minutes | SPPB, OLS Test, CoPSway, SDML, SDAP, VML, VAP (cm², cm, cm/s), 10-m Gait Speed Test (m/s) | 0 |
| 13 | Chan et al. (2024) | Community-dwelling Older adults n=42 age:69.8±5.7 | G1: Nintendo Ring Fit Adventure (NRFA)-based exercise program. Content: yoga, lower limb strengthening, balance, and rhythmic exergames. Three progressive stages (easy/medium/hard) with increasing repetitions and variations. Stage 3 included standing on a balance board for selected exercises. Supervised by a trained exercise instructor throughout with verbal feedback, manual support, and intensity adjustment G2: Usual care. Participants received advice from the exercise instructor on how to improve physical performance and continued to receive care from the community center and other healthcare providers for 8 weeks; no structured exercise program | 8 weeks/2 sessions a week/16 sessions/60 minutes | TUG, Mini-BESTest, 30s STS, Five-Time-Sit-to-Stand Test, Iconographical Falls Efficacy Scale, Color Trails Test (CTT-1, CTT-2, CTT-difference) | G1: 6 drop-outs: 3 personal circumstances (e.g., frequent medical appointments), 3 health issues unrelated to the intervention G2: 5 drop-outs: all due to personal issues |
| 14 | Rendon et al. (2012) | Community-dwelling Older adults n=40 age:85.7±4.3 | G1: Nintendo® Wii Fit + Wii Balance Board (WBB); 3 balance games (lunges, single leg extensions, twists); 3×/week × 6 weeks = 18 sessions; 35-45 min/session; supervised by physical therapist; assistive device available; 8-min warm-up + 8-min cool-down on stationary bicycle or recumbent stepper G2: Pre- and post-test measurements only; no intervention; instructed to maintain usual daily activities | 6 weeks/3 sessions a week/18 sessions/35-45 minutes | 8ftUG, FES-I/MFES, GDS | G1: Exergaming with balance platform 4 G2: No treatment (NT) 2 |
| 15 | Ku et al. (2019) | Community-dwelling Older adults n=36 age:65.63±5.18 | G1: 3D Interactive Augmented Reality System (3D-ARS) training. Uses a Kinect sensor to track whole-body motion and interact with virtual objects on a large screen. Includes three tasks: Balloon game (hip), Cave game (knee), and Rhythm game (single-leg stance) G2: Conventional physical fitness program (lower-extremity strengthening, balance, and endurance training) | 4 weeks/3 sessions a week/12 sessions/30 minutes | TUG, BBS, COP | G1: 0 G2: 2 |
| 16 | Liepa et al. (2022) | Community-dwelling Older adults n=44 age:71.67±5.97 | G1: Immersive VR exergame using HTC VIVE headset + chest-mounted sensor (tracking trunk movement) + balance board (Togu Balanza Ballstep), playing "Falling diamonds" game. 20 min gameplay + 5 min warm-up, 2x/week for 9 weeks G2: Non-immersive exergame using same chest sensor + balance board, but played on a laptop screen without VR headset G3: No intervention, maintained usual daily activities | 9 weeks/2 sessions a week/18 sessions/25 minutes | SPPB | 0 |
| 17 | Pluchino et al. (2012) | Community-dwelling older adults, n = 40; age:72.5 ± 8.4 | G1: Balance training using Wii Balance Board with 8 games (soccer heading, ski slalom, ski jump, tightrope walk, penguin slide, etc.). Difficulty adjusted based on game scores G2: Sun-style Tai Chi, 12 movements, taught by a certified instructor. G3: 14 functional balance activities (standing on compliant surfaces, walking over obstacles, single-leg stance, etc.) | 8 weeks/2 sessions a week/16 sessions/50 minutes | SLS; TUG; FRT; POMA; COP | G1: 4 G2: 3 G3: 6 |
| 18 | Schättin et al. (2016) | Community-dwelling older adults, n = 29; age:79.2 ± 7.3 | G1: Cognitive-motor training using a pressure-sensitive dance platform (Impact Dance Platform, 87.5×87.5cm) with foot pushes on arrows (right, left, up, down) to interact with video games. Games: Balloon, Step, Season, Space. 30 min/session (5 min warm-up + 20 min gameplay + 5 min cool-down), 3x/week for 24 sessions (8-10 weeks). Difficulty individually adjusted G2: Conventional balance training with repetitive static and dynamic exercises on stable and unstable surfaces. 30 min/session, 3x/week for 24 sessions. Progression through changes in base of support, eye closure, external perturbations, etc | 8 weeks/3 sessions a week/24 sessions/30 minutes | Gait Speed | G1: 1 G2: 1 |
| 19 | Karahan et al. (2015) | Community-dwelling older adults, n = 100; age:71.4 ± 5.4 | G1: Xbox 360 Kinect™ console with exergames (Kinect Adventures, Kinect Sports, Kinect Sports Season Two), including soccer, tennis, table tennis, skiing, golf, volleyball, bowling simulations. Supervised by a nurse G2: Home-based balance exercise including stretching (hamstring, quadriceps, pelvic girdle, pectoral muscles), strengthening (quadriceps, hamstring, biceps, abdominal muscles), standing on two feet, semi-tandem stance, single-leg stance, heel standing, toe standing | 6 weeks/5 sessions a week/30 sessions/30 minutes | BBS; TUG | G1: 6 G2: 4 |
| 20 | Kwok & Pua (2016) | Community-dwelling older adults, n = 80; age:69.4 ± 5.6 | G1: Supervised 1-hour session weekly (20 min WiiActive gaming + resistance band) + home exercises (≥2 days/week). 12 weeks total (12 sessions). Used Wii Balance Board G2: Supervised 1-hour session weekly (stationary bike, treadmill, elliptical, free weights, balance discs, gym ball) + same home exercises. 12 weeks | 12 weeks/1 sessions a week/12 sessions/60 minutes | TUG; Gait Speed; Fall Rate; Fear of Falling | G1: 5 G2: 11 |
| 21 | De Lima et al. (2021) | Community-dwelling older adults, n = 38; age:69.4 ± 5.6 | G1: Xbox Kinect console with game “Your Shape Fitness evolved”, including Zen (stretching/balance/flexibility), Pump it, Wall Breaker (boxing-like), Kick it (soccer), Hurricane, Stack in Up (balance) G2: No intervention, maintained usual daily activities, instructed not to change lifestyle | 6 weeks/3 sessions a week/18 sessions/60 minutes | 8-FUG; Chair sit-stand 30s; 6-MWT | G1: 5 G2: 4 |
| 22 | Whyatt et al. (2015) | Facility-dwelling older adults, n = 84; age:76.9 ± 6.9 | G1: Custom-made balance games using Wii Balance Board (WBB), including Apple Catch (mediolateral COP control), Bubble Pop, Avoid the Shark, Smart Shrimp (multidimensional COP control + cognitive tasks) G2: Recorded daily activity (activity diary), no structured exercise | 5 weeks/2 sessions a week/10 sessions/30 minutes | COP; BBS | G1: 2 G2: 0 |
| 23 | Sonthikul et al. (2022) | Community-dwelling older women, n = 34; age:63.3 ± 2.3 | G1: Active video gaming using a movable balance platform (Sensamove Miniboard), controlling a ball on screen through weight shifting G2: Core stabilization exercise (abdominal hollowing, bridging, lower trunk rotation, plank, etc.).Supervised by physiotherapist, home exercises monitored by phone and logbook | 6 weeks/3 sessions a week/18 sessions/60 minutes | TUG; Five Times Sit to Stand Test | G1: 2 G2: 2 |
| 24 | Zhao et al. (2022) | Community-dwelling older adults, n = 48; age:65.7 ± 3.8 | G1: Active video games using Nintendo Switch + Joy-Con controllers, including: (1) Zumba Fitness (dance game); (2) Fitboxing2 (boxing virtual game); (3) Mario Tennis Ace (tennis game). Designed based on Self-Determination Theory (SDT) emphasizing competence, autonomy, and relatedness G2: No intervention, continued usual daily activities | 12 weeks/3 sessions a week/36 sessions/50-55 minutes | COP (Kistler 9287CA) | G1: 2 G2: 8 |
| 25 | Yang et al. (2020) | Community-dwelling older adults, n = 20; age:68.7 | G1: Exergame training using Xbox Kinect with game “Your Shape: Fitness Evolved II”, including Zen Energy, Yoga, Destination Bollywood, Cardio Boxing, Humana Vatility, Cardio G2: Balance training program based on Campbell et al. (1997), including lower-body strengthening (squats, leg lifts), tandem standing, tandem walking, single-leg stance, etc | 5 weeks/2 sessions a week/10 sessions/45 minutes | TUG; FRT; SLS; 30s STS | 0 |
| 26 | Toulotte et al. (2012) | Community-dwelling older adults, n = 36; age:75.1 ± 10.2 | G1: Adapted Physical Activities including step length/height exercises, cervical spine mobility, ocular mobility, strength, proprioception, flexibility, static/dynamic balance G2: Nintendo Wii Fit console training with games including soccer heading, ski jump, yoga, downhill skiing, balance balls, tightrope walk G3: 30 min APA + 30 min Wii Fit G4: No training, watched television, played board games | 20 weeks/1 sessions a week/20 sessions/60 minutes | SLS; Tinetti; The position of center of gravity (percentage of difference) | 0 |
| 27 | Gallardo-Meza et al. (2022) | Community-dwelling older women, n = 82; age:68.7 ± 3.5 | G1: Nintendo Wii Fit Plus with Wii Balance Board. Games: (1) Sky Slalom (10 min); (2) Sky Jump (10 min); (3) Balance Bubble (10 min). 2 sessions/week for 4 weeks (8 sessions) G2: No additional intervention, maintained usual daily activities | 4 weeks/2 sessions a week/8 sessions/30 minutes | TUG; SLS | G1: 6 G2: 4 |
| 28 | Singh et al. (2013) | Community-dwelling older women, n = 38; age:77.2 ± 7.0 | G1: Balance-focused interactive virtual-reality games using Nintendo Wii Balance Board, including Ski Slalom, Table Tilt, Penguin Slide, Soccer Heading, Tight Rope Walk, Perfect 10, Tilt City G2: Therapeutic balance exercises, including one-leg standing, free leg swinging, moving objects forward/backward/sideways, walking in place/sideways/backwards | 6 weeks/2 sessions a week/12 sessions/40 minutes | TUG; COP (Probalance (OPI)) | G1: 1 G2: 1 |
| 29 | Wu et al. (2010) | Community-dwelling older adults, n = 64; age:69.8 ± 3.8 | G1: Telecommunication-based Tai Chi exercise. Participants exercised at home connected to instructor via custom video conferencing device (DocBox). 24-form Yang-style Tai Chi G2: Community center-based Tai Chi exercise. Participants exercised at YMCA with live instructor G3: Home video-based Tai Chi exercise. Participants exercised at home using a custom DVD set (45 one-hour sessions). No real-time interaction | 2 weeks/7 sessions a week/10-14 sessions/30-45 minutes | SLS; TUG; COP; Fear of Falling; Fall Rate; QOL | 0 |
| 30 | Pichierri et al. (2012) | Facility-dwelling older adults, n = 31; age:86.2 ± 4.6 | G1: 2 sessions/week for 12 weeks (24 sessions). ~40 min/session including warm-up (5 min), progressive resistance training (25 min, core and lower extremities, using weight vests), balance exercises (10 min, using air-filled cushions and grip balls). Additionally, 10-15 min of dance video game (StepMania on metal dance pad) after each session, following on-screen arrows. G2: Same strength and balance training (content, frequency, duration as dance group), without dance video game | 12 weeks/2 sessions a week/24 sessions/40-50 minutes | Gait analysis (Velocity, cm/s);(Cadence, steps/min);(Step length, cm); Fear of Falling (FES-I) | G1: 5 G2: 5 |
| 31 | Hou & Li (2022) | Community-dwelling older adults, n = 84; age:67.5 ± 4.6 | G1: Dance mat version of Stepmania game. Participants stepped on the dance mat following on-screen arrows (forward, backward, left, right). 3 sessions/week, 30 min gameplay + 5 min warm-up/cool-down for 12 weeks (36 sessions). 18 songs, 4 difficulty levels. G2: Computer keyboard version of Stepmania game. Participants pressed keyboard keys with fingers following the same game content. 3 sessions/week, 30 min gameplay + 5 min warm-up/cool-down for 12 weeks (36 sessions). G3 : No intervention, maintained usual daily activities. | 12 weeks/3 sessions a week/36 sessions/30 minutes | Balance (z, tug); (Lower limb strength); (Lower limb flexibility); (Aerobic endurance) | G1: 5 G2: 7 G3: 6 |
| 32 | Sadeghi et al. (2021) | Community-dwelling older men, n = 64; age:71.8 ± 5.9 | G1: Traditional balance training (single-leg stance, heel/toe standing, tandem stance, walking forward/backward, weight shifting). 3 sessions/week, 40 min/session (5 min warm-up + 30 min training + 5 min cool-down) for 8 weeks. Progressive intensity. G2: Xbox Kinect virtual reality training with games Light Race, Target Kick, Goalkeeper. 3 sessions/week, 40 min/session for 8 weeks. Progressive difficulty. G3 : 15 min BT + 15 min VR + 5 min warm-up/cool-down, 3 sessions/week for 8 weeks. G4: Waitlist control, continued usual daily activities, offered training after study. | 8 weeks/3 sessions a week/24 sessions/40 minutes | TUG; SLS; Tandem; Stance | G1: 1 G2: 2 G3: 2 G4: 1 |
| 33 | Bacha et al. (2018) | Community-dwelling older adults, n = 46; age:69.3 ± 5.3 | G1: Xbox Kinect Adventures games (Space Pop, 20,000 Leaks, Reflex Ridge, River Rush). 1 h/session, 2 sessions/week for 14 sessions. Full-body motion interaction with visual/auditory feedback. G2: Conventional physical therapy including warm-up, static/dynamic balance, endurance, muscle strength, coordination, stretching, cool-down. 1 h/session, 2 sessions/week for 14 sessions. | 7 weeks/2 sessions a week/14 sessions/60 minutes | Mini-BESTest; FGA | G1: 2 G2: 2 |
| 34 | Franco et al. (2012) | Facility-dwelling older adults, n = 32; age:78.3 ± 6.0 | G1: Balance games on Wii Balance Board (soccer heading, ski jump, ski slalom, tightrope, table tilt, balance bubble). 2 individual sessions/week ×10-15 min + daily home exercises (15 min) for 3 weeks (6 sessions). G2: Matter of Balance exercise component (strength + balance). Group sessions, 2 sessions/week ×30-45 min for 3 weeks (6 sessions). G3 : No intervention, maintained usual daily activities. | 3 weeks/2 sessions a week/6 sessions/25-30 minutes | BBS; Tinetti; SF-36 | G1: 3 G2: 1 G3: 0 |
| 35 | Lim et al. (2017) | Facility-dwelling older adults, n = 20; age:79.1 ± 5.9 | G1: Complex VR training using Wii-Fit Plus (balance + strength + flexibility + aerobic). 10 sessions (2/week, 60 min) over 5 weeks. G2: Balance-only VR training using same games (6 balance games). Same frequency and duration. | 5 weeks/2 sessions a week/10 sessions/60 minutes | TUG | 0 |
| 36 | Park & Yim (2016) | Facility-dwelling older adults, n = 72; age:73.5 ± 2.9 | G1: Conventional exercise (30 min, side stepping, tandem walking, backward walking, one-leg standing, etc.) + 3D virtual reality kayak program (20 min, using real paddle, 3D projection of river/lake). 2 sessions/week for 6 weeks (12 sessions). G2: Conventional exercise only (30 min, same content as experimental group). 2 sessions/week for 6 weeks. | 6 weeks/2 sessions a week/12 sessions/50 minutes | COP: Good Balance system (COP); (MoCA, 0-30) |  |
| 37 | Padala et al. (2017) | Community-dwelling older adults, n = 30; age:68.0 ± 6.7 | G1: Wii-Fit balance board training including yoga, strength, aerobics, balance games, Training Plus. 45 min/session, 3 sessions/week for 8 weeks (24 sessions). Self-selected games with encouragement to progress. G2: Computer-based cognitive training (Brain-Fitness program) covering memory, language, attention, executive function, visuospatial domains. Same frequency and duration, no exercise component. | 8 weeks/3 sessions a week/24 sessions/45 minutes | BBS | G1: 3 G2: 0 |
| 38 | Bieryla & Dold (2013) | Community-dwelling older adults, n = 12; age:81.5 ± 4.7 | G1: Wii Fit training (yoga: half moon, chair, warrior; aerobic: torso twists; balance games: soccer heading, ski jump). 30 min/session, 3 sessions/week for 3 weeks (9 sessions). One-on-one supervision, no physical assistance, chairs for safety. G2: Continue normal daily activities, no intervention | 3 weeks/3 sessions a week/9 sessions/30 minutes | BBS; TUG; FAB; FRT | G1: 1 G2: 0 |
| 39 | Bieryla (2016) | Community-dwelling older adults, n = 13; age:82.3 ± 4.6 | G1: Xbox Kinect training including Your Shape: Fitness Evolved (Zen Sessions, 15 min yoga/tai chi) and Kinect Adventures (20,000 leaks, rally ball, reflex ridge, 15 min). 30 min/session, 3 sessions/week for 3 weeks (9 sessions). One-on-one supervision, verbal instructions, no physical assistance. G2: Normal daily activities, no intervention | 3 weeks/3 sessions a week/9 sessions/30 minutes | BBS; TUG; FAB; FRT | G1: 1 G2: 0 |
| 40 | Sato et al. (2015) | Community-dwelling older adults, n = 57; age:69.2 ± 5.4 | G1: Exergame using Kinect sensor, including apple game (reaching), tightrope standing game (tandem stance reaching), balloon popping game (squatting), one-leg standing game (knee lifting). 1-2 sessions/week for 24 sessions, 40-60 min/session. G2: No intervention, usual daily activities | 12-24 weeks/1-2 sessions a week/24 sessions/40-60 minutes | Gait analysis; BBS; FRT; 30-second chair_x0002_stand (CS-30 test) test | G1: 1 G2: 2 |
| 41 | Delbaere et al. (2021) | Community-dwelling older adults, n = 503; age:69.8 ± 3.8 | G1: StandingTall telerehabilitation balance training delivered via tablet computer at home. Includes standing balance, targeted stepping, and step-up exercises. Minimum 2 hours/week for 2 years. Two home visits in first month and incidental phone calls in first 6 months, then unsupervised. G2: Health education (weekly fact sheets) delivered via tablet, no exercise component. Same frequency of contact. | 2 years/2 hours a week/208 sessions | TUG; 5XSST; SPPB | G1: 29 G2: 26 |
| 42 | Phirom et al. (2020) | Community-dwelling older adults, n = 40; age:69.8 ± 3.8 | G1: Xbox Kinect exergames (Fruits Hunter, Where am I?, Whack a mole, Sky fall, Crossing poison river) simultaneously training physical (stepping, balance) and cognitive functions. 3 sessions/week, 60 min/session for 12 weeks (36 sessions). G2: Educational material (fall prevention and cognitive enhancement) + weekly phone call, no exercise. | 12 weeks/3 sessions a week/36 sessions/60 minutes | PPA; TUG; Postural sway (mm) | G1: 1 G2: 0 |
| 43 | Lee (2021) | Community-dwelling older adults, n = 56; age:80.2 ± 6.5 | G1: Virtual reality gait training using smartphone app (BitGym) providing video flow synchronized with walking speed, on a non-motorized (self-powered) treadmill. 5 sessions/week, 50 min/session (10 min warm-up + 30 min main + 10 min cool-down) for 4 weeks (20 sessions). G2: Conventional treadmill training using the same non-motorized treadmill, no virtual reality video. Same frequency and duration. | 4 weeks/5 sessions a week/20 sessions/50 minutes | SLS; BBS; FRT; TUG | 0 |
| 44 | Adcock et al. (2020) | Community-dwelling older adults, n = 37; age:73.9 ± 6.4 | G1: In-home multicomponent exergame including Tai Chi-inspired exercises, dancing, and step-based cognitive games. Uses IMU sensors (wrists and ankles) for motion capture. 3 sessions/week, 30-40 min/session for 16 weeks (48 sessions). Progressive difficulty. G2: Usual care, continued normal daily living | 8 weeks/3 sessions a week/24 sessions/50 minutes | Gait analysis; SPPB | G1: 3 G2: 3 |
| 45 | Morat et al. (2019) | Community-dwelling older adults, n = 51; age:69.4 ± 5.6 | G1: Volitional stepping exergames under unstable conditions (Dividat Senso platform suspended on springs). 11 stepping games training cognitive and motor abilities. 3 sessions/week, 40 min/session (10-12 min net gaming) for 8 weeks (24 sessions). G2: Volitional stepping exergames under stable conditions (fixed platform). Same games and training parameters. G3 : No intervention, maintained usual daily activities. | 8 weeks/3 sessions a week/24 sessions/40 minutes | TUG; COP | G1: 2 G2: 1 G3: 0 |
| 46 | Benitez-Lugo et al. (2022) | Community-dwelling older adults, n = 48; age:72.6 ± 5.9 | G1: Nintendo Wii Balance Board games “Penguin Slide” (weight shifting) and “Step Plus” (stepping up/down). 2 sessions/week, 30 min/session for 8 weeks (16 sessions). Also received usual care (memory and joint mobility workshops). G2: Usual care only (memory and joint mobility workshops), no additional exercise intervention. | 8 weeks/2 sessions a week/16 sessions/30 minutes | TUG; BBS; Tinetti Test | G1: 2 G2: 0 |
| 47 | Jorgensen et al. (2013) | Community-dwelling older adults, n = 58; age:76.4 ± 6.6 | G1: Nintendo Wii Balance Board training including 5 balance games and 1 strengthening exercise (standing rowing squat). 2 sessions/week, ~35 min net training for 10 weeks (20 sessions). Supervised by physiotherapist. G2: Daily use of EVA shoe insoles, told to improve balance (placebo control). No exercise intervention. | 10 weeks/2 sessions a week/20 sessions/35 minutes | TUG, 30s STS, Short Falls Efficacy Scale-International (FES-I), RFD (N/s) | G1: 3 G2: 0 |
| 48 | Schwenk et al. (2014) | Facility-dwelling older adults, n = 33; age:84.6 ± 6.9 | G1: Balance training using wearable sensors (LegSys, 5 inertial sensors) with interactive game-based interface. Includes: (1) ankle point-to-point reaching tasks (weight shifting, AP/ML directions, with visuomotor rotation); (2) virtual obstacle crossing tasks (alternating leg lifting to cross virtual boulders). 2 sessions/week, 45 min/session for 4 weeks (8 sessions). Real-time visual/auditory feedback from sensors. G2: No intervention, maintained usual daily activities | 4 weeks/2 sessions a week/8 sessions/45 minutes | COM; AST; TUG; Gait analysis | G1: 2 G2: 1 |
| 49 | Lee et al. (2015) | Community-dwelling older adults, n = 54; age:68.2 ± 4.4 | G1: Xbox Kinect + “Zen Energy” game (Tai Chi-style movements). Real-time visual and auditory feedback with 3D avatar. 3 sessions/week, 60 min/session for 8 weeks (24 sessions). G2: Group-based exercise (postural, balance, functional, lower body coordination and strength training) with auditory feedback from physiotherapist. Same frequency and duration. | 8 weeks/3 sessions a week/24 sessions/60 minutes | TUG (8FUGT), 30s STS, 2-Minute Step Test; SF-36 | G1: 4 G2: 3 |
| 50 | Fakhro et al. (2020) | Community-dwelling older adults, n = 64; age:72.2 ± 5.2 | G1: Nintendo Wii Fit balance board training, games: first 4 weeks “Soccer Heading”, last 4 weeks “Table Tilt”. 40 min/session (5 min warm-up + 30 min gaming + 5 min cool-down), 3 sessions/week for 8 weeks (24 sessions). G2: No intervention, maintained usual daily activities | 8 weeks/3 sessions a week/24 sessions/40 minutes | TUG | G1: 3 G2: 1 |
| 51 | Moreira et al. (2021) | Community-dwelling older adults, n = 99; age:70.8 ± 5.1 | G1: Xbox 360 (Microsoft) with Kinect motion capture, using "Your Shape™: Fitness Evolved." Structure: 5 min warm-up (global movements), 25 min strength training (squats and lunges, 4-6 sets × 8-16 reps), 15 min balance and cardiorespiratory training (boxing, lateral and anteroposterior displacements), 5 min cool-down (Tai Chi Chuan slow-pace). Progression determined by in-game difficulty levels. G2: Traditional multicomponent exercise: knee/hip flexion-extension, hip adduction-abduction, internal/external rotation, ankle dorsi-plantar flexion (2-3 sets × 8-12 reps, 60 s rest between sets), using ankle cuff weights 1-4 kg (initial increment ~3% body mass); balance exercises: single-leg stance, semi-tandem, tandem, forward/backward/lateral displacements, plantar/dorsiflexion walking. | 12 weeks/3 sessions a week/36 sessions/50 minutes | TUG; MiniBESTest; Gait analysis; 4MWT\10MWT (m/s); Falls Efficacy Scale-International | G1: 17 G2: 16 |
| 52 | Chen (2020) | Community-dwelling older adults, n = 28; age:73.6 ± 4.1 | G1: AR-assisted selected Tai-Chi (sTC): Microsoft Kinect motion capture sensor used to detect full-body movements and construct digital skeleton. Skeletal data compared in real-time with Tai-Chi master's movements, providing visual feedback. 8 simplified movements extracted from 24-form Yang-style Tai-Chi, individually assigned based on each participant's maximum COP displacement and endurance; progressive in difficulty G2: Traditional Tai-Chi (tTC): Complete 24-form Yang-style Tai-Chi under instruction of a certified Tai-Chi master; typically 2-3 complete sequences per session | 8 weeks/3 sessions a week/24 sessions/30 minutes | BBS; TUG; FRT | 0 |
| 53 | Khanmohammadi (2021) | Community-dwelling older adults, n = 66; age:71.5 ± 5.0 | G1: Nintendo Wii Fit balance board (WBB) training; 6 balance games (2 static: single leg extension, torso twist; 4 dynamic: table tilt, penguin fishing, soccer heading, tightrope walk); games randomly selected each session, ~10 min per game; individualized progression across 3 difficulty levels G2: Motor-cognitive dual-task training with variable-priority instruction: simultaneous motor tasks (static/dynamic balance, walking, sit-to-stand, etc.) and cognitive tasks (verbal fluency, backward counting), alternating focus between motor and cognitive priority | 5.3 weeks/3 sessions a week/16 sessions/60 minutes | COP | G1: 4 G2: 5 |
| 54 | Yousefi Babadi & Daneshmandi (2021) | Facility-dwelling older adults, n = 36; age:66.9 ± 4.5 | G1: Virtual reality training (VRT) using Xbox Kinect (infrared camera motion-capture sensor). Games from Sports Pack 1 (boxing, table tennis, soccer) and Sports Pack 2 (golf, ski, American football); all performed standing, requiring upper and lower extremity use. Participant placed 1.5-2 m from sensor; real-time visual feedback provided on monitor G2: Conventional balance training (CBT) based on Hirase et al. (2015): 10 standing exercises in 3 progressive phases. Weeks 1-3: double-stance standing, heel/toe raises, walking in place; Weeks 4-6: adding neck hyperextension, free-leg swinging, trunk rotation; Weeks 7-9: adding floor touch, sideways walking, one-leg standing. Each session: 10 min warm-up + 40 min training + 10 min cool-down G3 : No treatment control (NT): maintained routine daily activities, no specific exercise | 9 weeks/3 sessions a week/27 sessions/60 minutes | TUG; FAB; SLS; Fullerton Advanced Balance Scale (FAB) | 0 |
| 55 | Kim & Cho (2022) | Facility-dwelling older adults, n = 36; age:79.9 ± 7.1 | G1: Nintendo Wii Fit program (Wii Balance Board + remote); games: Balance ski, Table tile (weight transfer/support/speed), Jogging, Rhythm step (aerobic/muscular endurance/strength); 5 min warm-up + 5 min per game + 5 min rest G2: Motor Imagery Training (MIT); conducted at 18:00 in quiet, dimly lit room; seated; guided by auditory cues; motor-sensory imagery (foot pressure, weight-shift sensation) + visual-sensory imagery (3rd-person perspective); 150s relaxation + 15 min main training G3 : No treatment (NT); no intervention | 6 weeks/3 sessions a week/18 sessions/30 minutes | COP; (FES) | G1: 0 G2: 2 G3: 0 |
| 56 | Bao (2018) | Community-dwelling older adults, n = 12; age:75.8 ± 5.2 | G1: In-home balance training with vibrotactile sensory augmentation (SA) via smartphone balance trainer. Each session: 6 exercise categories (static standing, compliant surface standing, weight shifting, modified COG, gait), 1-2 exercises per category, 6 reps each for 30 s. Vibrotactile SA provided during 4 of 6 repetitions per exercise (67% feedback). Device: two Apple iPod Touch units (sensing + user interface) + elastic belt + tactor bud with 4 vibration motors worn at L4/L5 G2: Same in-home balance training protocol; wore identical device but received NO vibrotactile SA | 8 weeks/3 sessions a week/24 sessions/45 minutes | Mini-BESTest; 5XSST; FRT; TUG; Gait analysis | 0 |
| 57 | Bao et al. (2022) | Community-dwelling older adults, n = 16; age:75.4 ± 4.7 | G1: 8-week home-based balance training (24 sessions) with vibrotactile sensory augmentation (SA): smartphone-based balance trainer (two iPods); SA device mounted at L4/L5; vibrotactile cues delivered to navel, spine, and bilateral sides when trunk motion exceeded thresholds; instruction: "move away from the vibration"; exercise types: static standing (firm/compliant surfaces), weight shifting, modified center of gravity (arm raises), gait (no SA during gait) G2: Identical 8-week home-based balance training (24 sessions) using same device but without vibrotactile SA activation | 8 weeks/3 sessions a week/24 sessions/60 minutes | TUG; Mini-BESTest; FRT; SOT (Sensory Organization Test composite score, NeuroCom/CDP); 5xSTS | G1: 2 G2: 2 |
| 58 | Yerlikaya (2021) | Community-dwelling older adults, n = 52; age:72.5 ± 6.9 | G1: Interactive Telerehabilitation Home Exercise (ITHE): participants exercised with a physiotherapist via video platforms (WhatsApp, Google Meet) in groups of 2-3; program included strengthening and balance exercises; 1-2 familiarization sessions conducted prior G2: Non-supervised Home Exercise (NHE): first session supervised by physiotherapist in person; subsequent sessions performed independently at home using instructional videos; therapist followed up via phone; same exercise content as ITHE G3 : no exercise intervention during the study period; exercise training and videos provided after study completion | 8 weeks/3 sessions a week/24 sessions/40 minutes | TUG; BBS; Sway Balance Mobile Application (SWAY Medical, 5, 0-100); WHOQOL-OLD; Trait Anxiety Inventory | G1: 0 G2: 1 G3: 1 |
| 59 | Gschwind et al (2015a) | Community-dwelling older adults, n = 148; age:80.9 ± 6.6 | G1: Microsoft Kinect® (3D depth sensor), 3 balance exergames (walking, stepping, weight-shifting based on WEBB) + 5 strength exercises (knee extension/flexion, hip abduction, calf/toe raises based on Otago Exercise Program), Movements recorded by Kinect and displayed as avatar on screen, Unsupervised, home-based, Balance exergames: 120 min/week; Strength exercises: 60 min/week, Balance exergames: 120 min/week; Strength exercises: 60 min/week G2: Pressure-sensitive electronic step mat (6-direction arrows), StepMania (divided attention, inhibition, processing speed); Stepper (processing speed, selective attention); Trail-stepping (visual attention, set-shifting); Tetris-stepping (visuospatial, problem-solving), Real-time stepping feedback via on-screen arrow cues, Unsupervised, home-based, Minimum 3 sessions × 20 min/week, including all 4 games, Selection of higher difficulty levels with greater cognitive load and stepping speed G3: Educational booklet on evidence-based health and fall prevention advice; asked to maintain usual activities | 16 weeks/3 sessions a week/48 sessions/20 minutes | TUG; COP (Sway meter, mm); Five Times Sit-to-Stand | G1: 10 G2: 15 G3: 10 |
| 60 | Gschwind et al (2015b) | Community-dwelling older adults, n = 153; age:74.7 ± 6.3 | G1: 16-week iStoppFalls ICT system: ① Balance exergames (Kinect motion capture): 3 games (Bumble Bee Park, Hills & Skills, Balance Bistro); recommended ≥3 sessions/week × ~40 min; progressive dual-task cognitive elements added at higher levels; ② Lower limb strength training (Otago-based): recommended ≥3 sessions/week × 15-20 min, 2-3 sets × 10-15 reps, progressive load with ankle cuff weights (1-3 kg); ③ Tablet-based reminders and automated fall risk monitoring; ④ Educational booklet G2: Educational booklet (healthy lifestyle and fall prevention advice) + maintenance of habitual exercise routines; no additional intervention | 16 weeks/3 sessions a week/48 sessions/40 minutes | TUG; SPPB; COP (Sway Area, mm²); 10m; Icon-FES (10, 10-40); EQ-5D (index + VAS) | G1: 15 G2: 13 |

Ages are means unless otherwise stated. Dose parameters are reported as extracted from each trial report; a blank cell indicates that the parameter was not reported. Dropouts are all-cause withdrawals as reported by arm. Trial numbers correspond to the order of citation in the main text (references 28-87). This table supersedes the per-study characteristics table of the previous version, which appeared in the main manuscript.

## **Table S5. Risk of bias (RoB 2) domain-level judgments for each included trial**

| **Trial** | **D1 randomization** | **D2 deviations** | **D3 missing data** | **D4 outcome measurement** | **D5 selective reporting** | **Overall** |
| --- | --- | --- | --- | --- | --- | --- |
| Toledano-Shubi et al. (2026) | Low | Some concerns | Some concerns | Low | Low | Some concerns |
| Campelo et al. (2023) | Low | Some concerns | Low | Some concerns | Some concerns | Some concerns |
| Parmak et al. (2025) | Low | Some concerns | Low | High | Some concerns | High |
| Abd El-Kafy et al. (2024) | Some concerns | Some concerns | Low | Low | Some concerns | Some concerns |
| Guede-Rojas et al. (2023) | Low | Some concerns | Low | Some concerns | Some concerns | Some concerns |
| Lee (2023) | Some concerns | Some concerns | Low | Low | Some concerns | Some concerns |
| Sheehy et al. (2025) | Low | Some concerns | Some concerns | Low | Low | Some concerns |
| Wang (2023) | Some concerns | High | Low | Low | Some concerns | Some concerns |
| Ghous et al. (2024) | Low | Some concerns | Low | Low | Some concerns | Some concerns |
| Elmas (2024) | Low | Some concerns | Low | Some concerns | Low | Some concerns |
| Park (2025) | Low | Some concerns | Low | Some concerns | Low | Some concerns |
| Gatica-Rojas (2025) | Low | Some concerns | Some concerns | Low | Some concerns | Some concerns |
| Chan et al. (2024) | Low | Some concerns | Some concerns | Low | Low | Some concerns |
| Rendon et al. (2012) | Some concerns | Some concerns | Some concerns | Low | Some concerns | Some concerns |
| Ku et al. (2019) | Low | Some concerns | Low | Low | Some concerns | Some concerns |
| Liepa et al. (2022) | Low | Some concerns | Low | Some concerns | Some concerns | Some concerns |
| Pluchino et al. (2012) | Some concerns | Some concerns | High | Some concerns | Some concerns | High |
| Schättin et al. (2016) | Low | Some concerns | Low | Some concerns | Low | Some concerns |
| Karahan et al. (2015) | Some concerns | Some concerns | Some concerns | Low | Some concerns | Some concerns |
| Kwok & Pua (2016) | Low | Some concerns | Some concerns | Low | Low | Some concerns |
| De Lima et al. (2021) | Low | Some concerns | Some concerns | Some concerns | Low | Some concerns |
| Whyatt et al. (2015) | Low | Some concerns | Low | Some concerns | Some concerns | Some concerns |
| Sonthikul et al. (2022) | Low | Some concerns | Low | Low | Some concerns | Some concerns |
| Zhao et al. (2022) | Low | Some concerns | Some concerns | Low | Some concerns | Some concerns |
| Yang et al. (2020) | Some concerns | Some concerns | Low | Low | Some concerns | Some concerns |
| Toulotte et al. (2012) | Some concerns | Some concerns | Low | Some concerns | Some concerns | Some concerns |
| Gallardo-Meza et al. (2022) | Low | Some concerns | Some concerns | Low | Some concerns | Some concerns |
| Singh et al. (2013) | Some concerns | Some concerns | Low | Low | Some concerns | Some concerns |
| Wu et al. (2010) | Low | Some concerns | Some concerns | Low | Some concerns | Some concerns |
| Pichierri et al. (2012) | Low | Some concerns | Some concerns | Low | Low | Some concerns |
| Hou & Li (2022) | Low | Some concerns | Some concerns | Low | Low | Some concerns |
| Sadeghi et al. (2021) | Low | Some concerns | Low | Low | Some concerns | Some concerns |
| Bacha et al. (2018) | Low | Some concerns | Low | Low | Low | Some concerns |
| Franco et al. (2012) | Some concerns | Some concerns | Some concerns | High | Some concerns | High |
| Lim et al. (2017) | Some concerns | Some concerns | Low | Low | Some concerns | Some concerns |
| Park & Yim (2016) | Low | Some concerns | Low | Some concerns | Some concerns | Some concerns |
| Padala et al. (2017) | Low | Some concerns | Low | High | Low | High |
| Bieryla & Dold (2013) | Some concerns | Some concerns | Some concerns | High | Some concerns | High |
| Bieryla (2016) | Some concerns | Some concerns | Low | High | Some concerns | High |
| Sato et al. (2015) | Low | Some concerns | Some concerns | Some concerns | Some concerns | Some concerns |
| Delbaere et al. (2021) | Low | Some concerns | Low | Low | Low | Some concerns |
| Phirom et al. (2020) | High | Some concerns | Low | Low | Some concerns | High |
| Lee (2021) | Low | Some concerns | Low | Low | Some concerns | Some concerns |
| Adcock et al. (2020) | Low | Some concerns | Some concerns | Low | Low | Some concerns |
| Morat et al. (2019) | Low | Some concerns | Some concerns | High | Some concerns | High |
| Benitez-Lugo et al. (2022) | Low | Some concerns | Some concerns | Low | Low | Some concerns |
| Jorgensen et al. (2013) | Low | Some concerns | Some concerns | Low | Low | Some concerns |
| Schwenk et al. (2014) | Low | Some concerns | Some concerns | Low | Low | Some concerns |
| Lee et al. (2015) | Low | Some concerns | Some concerns | Some concerns | Some concerns | Some concerns |
| Fakhro et al. (2020) | Low | Some concerns | Some concerns | Low | Low | Some concerns |
| Moreira et al. (2021) | Low | Some concerns | Some concerns | Low | Low | Some concerns |
| Chen (2020) | Some concerns | Low | Low | Low | Some concerns | Some concerns |
| Khanmohammadi (2021) | Low | Some concerns | Low | Low | Some concerns | Some concerns |
| Yousefi Babadi & Daneshmandi (2021) | Some concerns | Some concerns | Low | Some concerns | Low | Some concerns |
| Kim & Cho (2022) | Low | Some concerns | Some concerns | Low | Low | Some concerns |
| Bao (2018) | Some concerns | Some concerns | Low | Low | Some concerns | Some concerns |
| Bao et al. (2022) | Some concerns | Some concerns | Low | Low | Some concerns | Some concerns |
| Yerlikaya (2021) | Low | Low | Low | Low | Low | Low |
| Gschwind et al (2015a) | Low | Some concerns | Low | Low | Some concerns | Some concerns |
| Gschwind et al (2015b) | Low | Some concerns | Low | Low | Low | Low |
| **Summary (n=60)** | 42 Low | 2 Low | 34 Low | 6 High | 23 Low | **2 / 50 / 8** |

Overall summary row reports low risk / some concerns / high risk. D2 was rated as some concerns or high risk in 58 of 60 trials (97%), overwhelmingly because participants cannot be blinded to an exercise intervention; in the GRADE assessment this was not by itself treated as a reason to rate down, as set out in the Methods.

## **Table S6. Distribution of extractable effect modifiers across treatment nodes (transitivity assessment, Network 1)**

| **Node** | **Trials, n** | **Mean age, y** | **Duration, wk** | **Frequency/wk** | **Total sessions** | **Session length, min** |
| --- | --- | --- | --- | --- | --- | --- |
| NT | 32 | 73.8 (72.2) | 11.5 (8.0) | 3.0 | 20 | 40.5 (40) |
| CE | 29 | 71.9 (71.8) | 8.1 (8.0) | 3.0 | 18 | 46.9 (45) |
| EBP | 21 | 73.7 (72.5) | 7.7 (6.0) | 3.0 | 18 | 38.8 (35) |
| EMC | 18 | 71.9 (71.1) | 8.2 (8.0) | 3.0 | 24 | 45.0 (43) |
| OSG | 10 | 71.7 (71.7) | 8.8 (8.0) | 3.0 | 18 | 48.9 (50) |
| Tele | 5 | 70.7 (69.8) | 30.0 (8.0) | 3.0 | 21 | 33.8 (33) |
| IW | 3 | 78.6 (75.8) | 6.7 (8.0) | 3.0 | 24 | 50.0 (45) |

Values are means with medians in parentheses, except frequency and total sessions, which are medians. The five nodes supported by 10 or more trials are closely comparable on every extractable modifier. The telerehabilitation node has a mean duration of 30.0 weeks but a median of 8 weeks, the mean being driven by a single 104-week trial. The following modifiers were reported too inconsistently to be tabulated: baseline balance impairment, baseline fall risk or fall history, frailty status, sex distribution, level of therapist supervision, comparator intensity, prior familiarity with the technology, and outcome measurement protocol.

## **Table S7. Sensitivity of the Timed Up and Go network to removal of trials contributing outlying direct conventional-exercise-versus-no-treatment evidence**

*Panel A. Trials contributing direct conventional-exercise-versus-no-treatment evidence in the Timed Up and Go network.*

| **Trial** | **Arms (participants)** | **Direct SMD (SE)** | **CE frequency** | **Removed** |
| --- | --- | --- | --- | --- |
| Campelo et al. (2023) | NT (11), CE (14), EBP (15) | 2.534 (0.549) | 3/week | Both specifications |
| Sadeghi et al. (2021) | NT (15), CE (14), EMC (15) | 2.361 (0.491) | 3/week | Both specifications |
| Yerlikaya (2021) | NT (16), CE (16), Tele (18) | 1.093 (0.381) | 3/week | Specification 2 only |
| Yousefi Babadi & Daneshmandi (2021) | NT (12), CE (12), EMC (12) | 0.898 (0.430) | 3/week | Retained |

Direct SMD is the trial-level Hedges g for conventional exercise versus no treatment, computed from post-intervention means and pooled post-intervention SDs exactly as for the main analysis; positive values favor conventional exercise. These are observed trial-level values, not model-based estimates, and they are the values quoted in the Results and Discussion. Sadeghi et al and Yousefi Babadi and Daneshmandi share the same design (NT, CE, EMC), so the 4 trials span 3 study designs. All 4 are three-arm trials containing a no-treatment arm, all randomized 11 to 18 participants per arm, and all prescribed conventional exercise 3 times per week, which is the median frequency across the 29 conventional-exercise arms in the review. The discrepancy between direct and indirect evidence is therefore not attributable to a small number of aberrant results, nor to conventional-exercise intensity.

*Panel B. Network estimates and rankings before and after removal.*

| **Node** | **Primary analysis (41 trials)** | **SUCRA** | **Specification 1: 2 trials removed (SMD >2.0)** | **SUCRA** | **Specification 2: 3 trials removed (SMD >1.0)** |
| --- | --- | --- | --- | --- | --- |
| EBP | 0.81 (0.42 to 1.20), P<.001 | 74.8 | 0.74 (0.43 to 1.05), P<.001 | 90.1 | 0.71 (0.41 to 1.01), P<.001 |
| Tele | 0.82 (0.16 to 1.49), P=.015 | 72.5 | 0.59 (0.09 to 1.10), P=.021 | 72.2 | 0.36 (−0.19 to 0.90), P=.198 |
| EMC | 0.79 (0.36 to 1.21), P<.001 | 71.7 | 0.46 (0.11 to 0.81), P=.010 | 58.2 | 0.41 (0.07 to 0.74), P=.019 |
| CE | 0.60 (0.21 to 0.98), P=.002 | 47.2 | 0.29 (−0.03 to 0.61), P=.072 | 33.7 | 0.21 (−0.11 to 0.53), P=.197 |
| OSG | 0.49 (−0.005 to 0.978), P=.052 | 39.5 | 0.40 (0.03 to 0.76), P=.035 | 49.3 | 0.37 (0.02 to 0.72), P=.038 |
| IW | 0.48 (−0.42 to 1.38), P=.297 | 41.3 | 0.32 (−0.42 to 1.06), P=.394 | 42.2 | 0.28 (−0.44 to 0.99), P=.446 |
| NT | Reference | 3.0 | Reference | 4.4 | Reference |
| **τ** | 0.581 |  | 0.405 |  | 0.378 |
| **Spearman ρ vs primary** | — |  | 0.86 |  | 0.68 |
| **Direct vs indirect, CE vs NT** | 1.612 vs 0.189, P<.001 |  | 1.007 vs 0.151, P=.010 |  | Not estimable (1 direct trial) |

SMD: standardized mean difference versus no treatment. The primary-analysis column reproduces the estimates and SUCRA values reported in Table 2 of the main text; the two sensitivity specifications were computed from the arm-level dataset underlying the four networks. Both specifications attenuate every estimate, and neither eliminates the direct-indirect discrepancy for conventional exercise versus no treatment. The two comparisons that carry moderate certainty in the primary analysis, exergaming with balance platforms and exergaming with motion capture, remain statistically significant under both specifications. Because removal does not resolve the discrepancy and does materially reduce precision, the primary analysis retains all trials and the conventional-exercise-versus-no-treatment comparison is instead rated down for inconsistency in the GRADE assessment.

## **Table S8. Network geometry: trials and participants contributing direct evidence to each contrast**

| **Contrast** | **Trials, n** | **Participants, n** |
| --- | --- | --- |
| ***Network 1: all balance*** | | |
| EBP vs NT | 14 | 616 |
| EMC vs CE | 11 | 485 |
| EBP vs CE | 10 | 338 |
| EMC vs NT | 9 | 321 |
| CE vs NT | 7 | 171 |
| OSG vs NT | 7 | 440 |
| OSG vs CE | 3 | 185 |
| Tele vs CE | 3 | 145 |
| Tele vs NT | 2 | 260 |
| IW vs CE | 2 | 27 |
| IW vs NT | 1 | 30 |
| EMC vs EBP | 1 | 63 |
| Tele vs EBP | 1 | 16 |
| ***Network 2: functional scales*** | | |
| EMC vs CE | 7 | 332 |
| EBP vs NT | 6 | 206 |
| CE vs NT | 4 | 95 |
| OSG vs NT | 4 | 264 |
| EMC vs NT | 3 | 53 |
| EBP vs CE | 3 | 56 |
| Tele vs NT | 2 | 260 |
| Tele vs CE | 2 | 115 |
| IW vs CE | 2 | 27 |
| OSG vs CE | 1 | 56 |
| ***Network 3: TUG*** | | |
| EBP vs NT | 9 | 441 |
| EMC vs CE | 9 | 395 |
| EMC vs NT | 7 | 236 |
| EBP vs CE | 6 | 219 |
| OSG vs NT | 5 | 373 |
| CE vs NT | 4 | 110 |
| Tele vs CE | 3 | 145 |
| Tele vs NT | 2 | 260 |
| OSG vs CE | 2 | 113 |
| IW vs CE | 2 | 27 |
| IW vs NT | 1 | 30 |
| EMC vs EBP | 1 | 63 |
| ***Network 4: gait speed*** | | |
| EMC vs NT | 2 | 61 |
| EBP vs CE | 2 | 48 |
| EMC vs CE | 2 | 95 |
| CE vs NT | 1 | 29 |
| OSG vs NT | 1 | 136 |
| Tele vs NT | 1 | 226 |
| IW vs NT | 1 | 30 |
| OSG vs CE | 1 | 56 |
| Tele vs CE | 1 | 81 |
| IW vs CE | 1 | 12 |
| Tele vs EBP | 1 | 16 |

Contrasts not listed for a given network have no direct evidence and are estimated indirectly. Across all four networks, direct evidence for technology-versus-technology comparisons is available for only a small number of contrasts, which is why most active-treatment comparisons rest wholly on indirect evidence.

## **Table S9. Exact *P* values and standard errors for all network estimates**

Effect estimates, standard errors and P values are those returned by the primary Stata analysis (mvmeta, REML) and are reported here at full precision; the confidence limits shown are the estimate ± 1.96 standard errors and therefore reproduce the intervals given in Tables 2 and 3 of the main text. P values are given to 3 decimal places where P<.10 and to 2 decimal places otherwise, whereas the main tables round to 2 decimal places throughout. This table covers all 44 network estimates; the GRADE profile in Table 3 rates 39 of them, the 5 versus-conventional-exercise contrasts in Network 4 not being separately rated because that network is reported as hypothesis-generating only.

| **Network** | **Comparison** | **SMD (95% CI), unrounded** | **SE** | **Exact *P*** |
| --- | --- | --- | --- | --- |
| Network 1 | Tele vs NT | 0.686 (0.078 to 1.295) | 0.310 | .027 |
| Network 1 | EBP vs NT | 0.631 (0.312 to 0.951) | 0.163 | <.001 |
| Network 1 | EMC vs NT | 0.582 (0.215 to 0.948) | 0.187 | .002 |
| Network 1 | OSG vs NT | 0.408 (−0.011 to 0.828) | 0.214 | .057 |
| Network 1 | IW vs NT | 0.331 (−0.565 to 1.227) | 0.457 | .47 |
| Network 1 | CE vs NT | 0.351 (0.025 to 0.677) | 0.166 | .035 |
| Network 1 | Tele vs CE | 0.335 (−0.261 to 0.931) | 0.304 | .27 |
| Network 1 | EBP vs CE | 0.280 (−0.067 to 0.627) | 0.177 | .11 |
| Network 1 | EMC vs CE | 0.231 (−0.110 to 0.571) | 0.174 | .18 |
| Network 1 | OSG vs CE | 0.057 (−0.409 to 0.524) | 0.238 | .81 |
| Network 1 | IW vs CE | −0.020 (−0.904 to 0.864) | 0.451 | .96 |
| Network 2 | IW vs NT | 1.099 (−0.278 to 2.475) | 0.702 | .12 |
| Network 2 | OSG vs NT | 0.409 (−0.247 to 1.064) | 0.334 | .22 |
| Network 2 | EMC vs NT | 0.371 (−0.291 to 1.033) | 0.338 | .27 |
| Network 2 | EBP vs NT | 0.183 (−0.436 to 0.802) | 0.316 | .56 |
| Network 2 | Tele vs NT | 0.145 (−0.682 to 0.971) | 0.422 | .73 |
| Network 2 | CE vs NT | 0.098 (−0.472 to 0.669) | 0.291 | .74 |
| Network 2 | IW vs CE | 1.000 (−0.253 to 2.253) | 0.639 | .12 |
| Network 2 | OSG vs CE | 0.310 (−0.479 to 1.100) | 0.403 | .44 |
| Network 2 | EMC vs CE | 0.273 (−0.241 to 0.786) | 0.262 | .30 |
| Network 2 | EBP vs CE | 0.085 (−0.610 to 0.779) | 0.354 | .81 |
| Network 2 | Tele vs CE | 0.046 (−0.786 to 0.878) | 0.425 | .91 |
| Network 3 | EBP vs NT | 0.811 (0.425 to 1.197) | 0.197 | <.001 |
| Network 3 | Tele vs NT | 0.824 (0.161 to 1.486) | 0.338 | .015 |
| Network 3 | EMC vs NT | 0.786 (0.362 to 1.211) | 0.217 | <.001 |
| Network 3 | CE vs NT | 0.596 (0.210 to 0.983) | 0.197 | .002 |
| Network 3 | IW vs NT | 0.478 (−0.421 to 1.378) | 0.459 | .30 |
| Network 3 | OSG vs NT | 0.487 (−0.005 to 0.978) | 0.251 | .052 |
| Network 3 | EBP vs CE | 0.215 (−0.207 to 0.637) | 0.215 | .32 |
| Network 3 | Tele vs CE | 0.227 (−0.411 to 0.866) | 0.326 | .49 |
| Network 3 | EMC vs CE | 0.190 (−0.193 to 0.573) | 0.196 | .33 |
| Network 3 | OSG vs CE | −0.110 (−0.663 to 0.444) | 0.282 | .70 |
| Network 3 | IW vs CE | −0.118 (−1.001 to 0.765) | 0.451 | .79 |
| Network 4 | EMC vs NT | 1.509 (−0.679 to 3.697) | 1.116 | .18 |
| Network 4 | CE vs NT | 1.153 (−0.824 to 3.130) | 1.009 | .25 |
| Network 4 | EBP vs NT | 0.945 (−1.807 to 3.696) | 1.404 | .50 |
| Network 4 | Tele vs NT | 0.910 (−1.527 to 3.347) | 1.243 | .46 |
| Network 4 | IW vs NT | 0.847 (−1.826 to 3.520) | 1.364 | .53 |
| Network 4 | OSG vs NT | 0.389 (−2.226 to 3.004) | 1.334 | .77 |
| Network 4 | EMC vs CE | 0.356 (−1.784 to 2.495) | 1.092 | .74 |
| Network 4 | EBP vs CE | −0.209 (−2.365 to 1.948) | 1.100 | .85 |
| Network 4 | Tele vs CE | −0.243 (−2.486 to 2.000) | 1.145 | .83 |
| Network 4 | IW vs CE | −0.306 (−3.001 to 2.389) | 1.375 | .82 |
| Network 4 | OSG vs CE | −0.764 (−3.385 to 1.857) | 1.337 | .57 |

## **Table S10. Random-effects meta-regression of the technology-versus-control effect on training-dose parameters**

| **Covariate** | **Trials, n** | **Slope (SE)** | **95% CI** | ***P* value** |
| --- | --- | --- | --- | --- |
| ***Network 1 (all balance)*** | | | | |
| Intervention duration (weeks) | 53 | -0.003 (0.003) | -0.009 to 0.002 | .212 |
| Training frequency (sessions/week) | 53 | 0.007 (0.091) | -0.172 to 0.185 | .942 |
| Total sessions (n) | 52 | -0.009 (0.007) | -0.022 to 0.004 | .168 |
| Session length (minutes) | 52 | -0.009 (0.005) | -0.019 to 0.001 | .076 |
| Weekly training dose (minutes/week) | 52 | -0.002 (0.002) | -0.006 to 0.001 | .143 |
| Mean age (years) | 53 | -0.018 (0.017) | -0.052 to 0.016 | .310 |
| ***Network 3 (TUG)*** | | | | |
| Intervention duration (weeks) | 40 | -0.004 (0.003) | -0.009 to 0.002 | .164 |
| Training frequency (sessions/week) | 40 | 0.010 (0.102) | -0.189 to 0.210 | .922 |
| Total sessions (n) | 39 | -0.014 (0.007) | -0.028 to 0.000 | .062 |
| Session length (minutes) | 39 | -0.007 (0.006) | -0.018 to 0.004 | .218 |
| Weekly training dose (minutes/week) | 39 | -0.002 (0.002) | -0.006 to 0.002 | .277 |
| Mean age (years) | 40 | -0.022 (0.019) | -0.059 to 0.015 | .258 |

Outcome: study-level standardized mean difference for the technology arm versus the within-trial control arm. No dose parameter reached statistical significance in either network. Slopes are per unit of the covariate. Because these analyses use study-level aggregate data they are subject to ecological bias and are underpowered to detect modest dose-response relationships.

## **Table S11. Random-effects meta-regression of the direct conventional-exercise-versus-no-treatment effect on comparator-intensity parameters (Network 1)**

| **Covariate** | **Trials, n** | **Slope (SE)** | **95% CI** | ***P* value** |
| --- | --- | --- | --- | --- |
| CE training frequency (sessions/week) | 7 | 1.457 (0.529) | 0.420 to 2.494 | .040 |
| CE weekly training dose (minutes/week) | 7 | 0.017 (0.011) | −0.008 to 0.038 | .178 |
| CE intervention duration (weeks) | 7 | -0.119 (0.113) | -0.341 to 0.102 | .339 |
| CE total sessions (n) | 7 | 0.068 (0.075) | -0.078 to 0.215 | .401 |
| CE session length (minutes) | 7 | -0.006 (0.044) | -0.092 to 0.079 | .890 |

Outcome: direct within-trial standardized mean difference for conventional exercise versus no treatment. Only training frequency reached nominal significance, and this analysis rests on 7 trials; it should be regarded as hypothesis-generating.

## **Table S12. Treatment ranking: SUCRA and probability of being the best treatment**

| **Intervention** | **SUCRA, %** | **Probability best, %** | **SUCRA, %** | **Probability best, %** |
| --- | --- | --- | --- | --- |
|  | **Network 1: all balance** | | **Network 3: Timed Up and Go** | |
| Tele | 76.3 | 41.2 | 72.5 | 35.9 |
| EBP | 75.2 | 21.9 | 74.8 | 25.5 |
| EMC | 68.7 | 16.3 | 71.7 | 21.2 |
| OSG | 47.2 | 5.4 | 39.5 | 3.5 |
| IW | 41.2 | 15.0 | 41.3 | 12.9 |
| CE | 36.6 | 0.2 | 47.2 | 0.9 |
| NT | 4.8 | 0.0 | 3.0 | 0.0 |

SUCRA values are those reported in Table 2 of the main text and sum to 350.0 (7 nodes × 50) within each network. Probabilities of being the best treatment are derived from the same 5000 parametric bootstrap draws used to compute SUCRA and sum to 100% within each network; columns may not sum to exactly 100.0 because of rounding. The substantial probability attaching to interventions with wearables despite its low SUCRA position arises from the extreme width of its confidence interval rather than from evidence of benefit.

## **Table S13. Domain-level GRADE judgments for every comparison rated**

| **Comparison** | **Studies, n** | **Participants, n** | **Risk of bias** | **Inconsistency** | **Indirectness** | **Imprecision** | **Publication bias** | **Certainty** |
| --- | --- | --- | --- | --- | --- | --- | --- | --- |
| **Network 1: all balance outcomes (55 studies)** | | | | | | | | |
| EBP vs NT | 21 | 1224 | Serious | — | — | Serious | — | Low |
| EMC vs NT | 18 | 1153 | Serious | — | — | Serious | — | Low |
| Tele vs NT | 5 | 991 | Serious | — | — | Serious | — | Low |
| OSG vs NT | 10 | 1111 | Serious | — | — | Serious | — | Low |
| IW vs NT | 3 | 822 | Serious | — | — | Very serious | — | Very low |
| CE vs NT | 29 | 1374 | Serious | — | — | Serious | — | Low |
| EBP vs CE | 21 | 1012 | Serious | — | — | Serious | — | Low |
| EMC vs CE | 18 | 941 | Serious | — | — | Serious | — | Low |
| Tele vs CE | 5 | 779 | Serious | — | — | Serious | — | Low |
| OSG vs CE | 10 | 899 | Serious | — | — | Serious | — | Low |
| IW vs CE | 3 | 610 | Serious | — | — | Very serious | — | Very low |
| **Network 2: functional scales (26 studies)** | | | | | | | | |
| IW vs NT | 2 | 399 | Serious | — | — | Very serious | — | Very low |
| OSG vs NT | 5 | 548 | Serious | — | — | Serious | — | Low |
| EMC vs NT | 9 | 568 | Serious | — | — | Serious | — | Low |
| EBP vs NT | 7 | 498 | Serious | — | — | Serious | — | Low |
| Tele vs NT | 3 | 560 | Serious | — | — | Serious | — | Low |
| CE vs NT | 15 | 671 | Serious | — | — | Serious | — | Low |
| IW vs CE | 2 | 300 | Serious | — | — | Very serious | — | Very low |
| OSG vs CE | 5 | 449 | Serious | — | — | Serious | — | Low |
| EMC vs CE | 9 | 469 | Serious | — | — | Serious | — | Low |
| EBP vs CE | 7 | 399 | Serious | — | — | Serious | — | Low |
| Tele vs CE | 3 | 461 | Serious | — | — | Serious | — | Low |
| **Network 3: Timed Up and Go test (41 studies)** | | | | | | | | |
| EBP vs NT | 14 | 940 | Serious | — | — | — | — | Moderate |
| EMC vs NT | 14 | 905 | Serious | — | — | — | — | Moderate |
| Tele vs NT | 4 | 823 | Serious | — | — | Serious | — | Low |
| CE vs NT | 22 | 1075 | Serious | Serious | — | — | — | Low |
| OSG vs NT | 7 | 878 | Serious | — | — | Serious | — | Low |
| IW vs NT | 3 | 662 | Serious | — | — | Very serious | — | Very low |
| EBP vs CE | 14 | 749 | Serious | — | — | Serious | — | Low |
| EMC vs CE | 14 | 714 | Serious | — | — | Serious | — | Low |
| Tele vs CE | 4 | 632 | Serious | — | — | Serious | — | Low |
| OSG vs CE | 7 | 687 | Serious | — | — | Serious | — | Low |
| IW vs CE | 3 | 471 | Serious | — | — | Very serious | — | Very low |
| **Network 4: gait speed (12 studies; supplementary and hypothesis-generating only)** | | | | | | | | |
| EMC vs NT | 3 | 285 | Serious | Serious | — | Very serious | — | Very low |
| CE vs NT | 7 | 367 | Serious | Serious | — | Very serious | — | Very low |
| EBP vs NT | 3 | 255 | Serious | Serious | — | Very serious | — | Very low |
| Tele vs NT | 3 | 388 | Serious | Serious | — | Very serious | — | Very low |
| IW vs NT | 2 | 244 | Serious | Serious | — | Very serious | — | Very low |
| OSG vs NT | 2 | 322 | Serious | Serious | — | Very serious | — | Very low |

Every comparison begins at high certainty because all contributing studies were randomized trials, and is rated down 1 level (serious) or 2 levels (very serious) per domain; an em dash denotes no rating down. The judgments shown are those summarized in the "Reasons for rating down" column of Table 3 of the main text, set out here domain by domain; the final column reproduces the certainty rating given there, and each rating is exactly recoverable from the domain judgments in its own row. Decision rules were fixed in advance of rating and are stated in full in the Methods and in the footnote to Table 3. Risk of bias was not rated down for the absence of participant blinding alone. Indirectness was not rated down for any comparison, as every trial was a randomized comparison in the target population using the intended outcomes. Publication bias was not rated down for any comparison, as no comparison was supported by the 10 or more trials required for a meaningful test of funnel plot asymmetry. Participant counts are the sum of arm-level n across contributing studies for both arms of each comparison. Intervention abbreviations are defined in the footnote to Table 2 of the main text. The 5 versus-conventional-exercise contrasts in Network 4 were not separately rated, that network being reported as hypothesis-generating only; all 44 network estimates appear in Table S9.

## **Figure S1. RoB 2 traffic-light plot**

## **Figure S2. effect-modifier distributions**

## **Figure S3. TUG sensitivity analysis**

## **Figure S4. dose-response meta-regression**

## **Figure S5. comparator-intensity meta-regression**

## **Figure S6. ranking probabilities**
