# Supplementary material for: Comparative Effectiveness of Digital Exercise-Based Interventions for Improving Balance in Older Adults: Systematic Review and Network Meta-Analysis With GRADE Certainty of Evidence
Source: JMIR Aging. 2026 Oct 28;9:e101231. doi: 10.2196/101231 (PMC13717434; doi:10.2196/101231)
Supplement: Supplementary file 3 [file aging-v9-e101231-s003.docx]

| **Section/Topic** | **Item #** | **Checklist item** | **Location where item is reported** |
| --- | --- | --- | --- |
| **TITLE** | | |  |
| Title | 1 | Identify the report as a systematic review incorporating a network meta-analysis (or related form of meta-analysis). | Title |
| **ABSTRACT** | | |  |
| Structured summary | 2 | Provide a structured summary including, as applicable: Background: main objectives. Methods: data sources; study eligibility criteria, participants, and interventions; study appraisal; and synthesis methods, such as network meta-analysis. Results: number of studies and participants identified; summary estimates with corresponding confidence/credible intervals; treatment rankings may also be discussed. Authors may choose to summarize pairwise comparisons against a chosen treatment included in their analyses for brevity. Discussion/Conclusions: limitations; conclusions and implications of findings. Other: primary source of funding; systematic review registration number with registry name. | Abstract |
| **INTRODUCTION** | | |  |
| Rationale | 3 | Describe the rationale for the review in the context of what is already known, including mention of why a network meta-analysis has been conducted. | Introduction: Background; Prior Work and Rationale for an Update |
| Objectives | 4 | Provide an explicit statement of questions being addressed, with reference to participants, interventions, comparisons, outcomes, and study design (PICOS). | Introduction: Objectives |
| **METHODS** | | |  |
| Protocol and registration | 5 | Indicate whether a review protocol exists and if and where it can be accessed (e.g., Web address); and, if available, provide registration information, including registration number. | Abstract (Trial Registration); Methods: Study Design and Registration |
| Eligibility criteria | 6 | Specify study characteristics (e.g., PICOS, length of follow-up) and report characteristics (e.g., years considered, language, publication status) used as criteria for eligibility, giving rationale. Clearly describe eligible treatments included in the treatment network, and note whether any have been clustered or merged into the same node (with justification). | Methods: Eligibility Criteria and Study Selection; Intervention Classification; Multimedia Appendix 3 (Table S3) |
| Information sources | 7 | Describe all information sources (e.g., databases with dates of coverage, contact with study authors to identify additional studies) in the search and date last searched. | Methods: Literature Search |
| Search | 8 | Present full electronic search strategy for at least one database, including any limits used, such that it could be repeated. | Methods: Literature Search; Multimedia Appendix 3 (Table S1) |
| Study selection | 9 | State the process for selecting studies (i.e., screening, eligibility, included in systematic review, and, if applicable, included in the meta-analysis). | Methods: Eligibility Criteria and Study Selection |
| Data collection process | 10 | Describe method of data extraction from reports (e.g., piloted forms, independently, in duplicate) and any processes for obtaining and confirming data from investigators. | Methods: Data Extraction |
| Data items | 11 | List and define all variables for which data were sought (e.g., PICOS, funding sources) and any assumptions and simplifications made. | Methods: Outcome Measures and Network Structure; Data Extraction |
| Geometry of the network | S1 | Describe methods used to explore the geometry of the treatment network under study and potential biases related to it. This should include how the evidence base has been graphically summarized for presentation, and what characteristics were compiled and used to describe the evidence base to readers. | Methods: Outcome Measures and Network Structure; Statistical Analysis (transitivity); Results: Network Structure |
| Risk of bias within individual studies | 12 | Describe methods used for assessing risk of bias of individual studies (including specification of whether this was done at the study or outcome level), and how this information is to be used in any data synthesis. | Methods: Risk of Bias Assessment |
| Summary measures | 13 | State the principal summary measures (e.g., risk ratio, difference in means). Also describe the use of additional summary measures assessed, such as treatment rankings and surface under the cumulative ranking curve (SUCRA) values, as well as modified approaches used to present summary findings from meta-analyses. | Methods: Statistical Analysis (Hedges g SMD; SUCRA and probability of being best) |
| Planned methods of analysis | 14 | Describe the methods of handling data and combining results of studies for each network meta-analysis. This should include, but not be limited to: handling of multi-arm trials; selection of variance structure; selection of prior distributions in Bayesian analyses; and assessment of model fit. | Methods: Statistical Analysis (augmented format for multi-arm trials; random-effects consistency model, REML, common τ) |
| Assessment of inconsistency | S2 | Describe the statistical methods used to evaluate the agreement of direct and indirect evidence in the treatment network(s) studied. Describe efforts taken to address its presence when found. | Methods: Statistical Analysis (design-by-treatment interaction model, node-splitting, post hoc sensitivity analysis) |
| Risk of bias across studies | 15 | Specify any assessment of risk of bias that may affect the cumulative evidence (e.g., publication bias, selective reporting within studies). | Methods: Statistical Analysis (comparison-adjusted funnel plots; GRADE) |
| Additional analyses | 16 | Describe methods of additional analyses if done, indicating which were pre-specified. This may include, but not be limited to, the following: sensitivity or subgroup analyses; meta-regression analyses; alternative formulations of the treatment network; and use of alternative prior distributions for Bayesian analyses (if applicable). | Methods: Study Design and Registration (post hoc analyses identified); Statistical Analysis |
| **RESULTS** | | |  |
| Study selection | 17 | Give numbers of studies screened, assessed for eligibility, and included in the review, with reasons for exclusions at each stage, ideally with a flow diagram. | Results: Study Selection and Characteristics; Figure 1; Multimedia Appendix 3 (Table S2) |
| Presentation of network structure | S3 | Provide a network graph of the included studies to enable visualization of the geometry of the treatment network. | Results: Network Structure; Figure 2 |
| Summary of network geometry | S4 | Provide a brief overview of characteristics of the treatment network. This may include commentary on the abundance of trials and randomized patients for the different interventions and pairwise comparisons in the network, gaps of evidence in the treatment network, and potential biases reflected by the network structure. | Results: Network Structure; Table 1; Multimedia Appendix 3 (Table S8) |
| Study characteristics | 18 | For each study, present characteristics for which data were extracted (e.g., study size, PICOS, follow-up period) and provide the citations. | Results: Study Selection and Characteristics; Multimedia Appendix 3 (Tables S3 and S4) |
| Risk of bias within studies | 19 | Present data on risk of bias of each study and, if available, any outcome level assessment. | Results: Risk of Bias; Multimedia Appendix 3 (Table S5 and Figure S1) |
| Results of individual studies | 20 | For all outcomes considered (benefits or harms), present, for each study: 1) simple summary data for each intervention group, and 2) effect estimates and confidence intervals. Modified approaches may be needed to deal with information from larger networks. | Results: Network Meta-Analysis Results (Figures 3 and 4); Multimedia Appendix 3 (Tables S4 and S8); Data Availability |
| Synthesis of results | 21 | Present results of each meta-analysis done, including confidence/credible intervals. In larger networks, authors may focus on comparisons versus a particular comparator (e.g., placebo or standard care), with full findings presented in an appendix. League tables and forest plots may be considered to summarize pairwise comparisons. If additional summary measures were explored (such as treatment rankings), these should also be presented. | Results: Network Meta-Analysis Results; Ranking Probabilities; Table 2; Figures 3 and 4; Multimedia Appendix 3 (Tables S9 and S12; Figure S6) |
| Exploration for inconsistency | S5 | Describe results from investigations of inconsistency. This may include such information as measures of model fit to compare consistency and inconsistency models, P values from statistical tests, or summary of inconsistency estimates from different parts of the treatment network. | Results: Inconsistency Testing; Multimedia Appendix 3 (Table S7 and Figure S3) |
| Risk of bias across studies | 22 | Present results of any assessment of risk of bias across studies for the evidence base being studied. | Results: Small-Study Effects and Publication Bias (Figure 5); Certainty of Evidence (Table 3); Multimedia Appendix 3 (Table S13) |
| Results of additional analyses | 23 | Give results of additional analyses, if done (e.g., sensitivity or subgroup analyses, meta-regression analyses, alternative network geometries studied, alternative choice of prior distributions for Bayesian analyses, and so forth). | Results: Inconsistency Testing; Transitivity Assessment; Meta-Regression of Training Dose; Acceptability, Adverse Events and User Burden (Figures 6 and 7); Multimedia Appendix 3 (Tables S6, S7, S10 and S11) |
| **DISCUSSION** | | |  |
| Summary of evidence | 24 | Summarize the main findings, including the strength of evidence for each main outcome; consider their relevance to key groups (e.g., healthcare providers, users, and policy-makers). | Discussion: Principal Findings; GRADE Certainty and Clinical Translation; Implementation Considerations |
| Limitations | 25 | Discuss limitations at study and outcome level (e.g., risk of bias), and at review level (e.g., incomplete retrieval of identified research, reporting bias). Comment on the validity of the assumptions, such as transitivity and consistency. Comment on any concerns regarding network geometry (e.g., avoidance of certain comparisons). | Discussion: Limitations; Conventional Exercise as a Problematic Comparator; The All-Balance Composite Network |
| Conclusions | 26 | Provide a general interpretation of the results in the context of other evidence, and implications for future research. | Discussion: Future Directions; Conclusions |
| **FUNDING** | | |  |
| Funding | 27 | Describe sources of funding for the systematic review and other support (e.g., supply of data); role of funders for the systematic review. This should also include information regarding whether funding has been received from manufacturers of treatments in the network and/or whether some of the authors are content experts with professional conflicts of interest that could affect use of treatments in the network. | Funding; Conflicts of Interest |

PICOS = population, intervention, comparators, outcomes, study design. Items S1-S5 are the items added by the network meta-analysis extension. *From:* Hutton B, Salanti G, Caldwell DM, Chaimani A, Schmid CH, Cameron C, et al. The PRISMA extension statement for reporting of systematic reviews incorporating network meta-analyses of health care interventions: checklist and explanations. Ann Intern Med 2015;162(11):777-784. doi: 10.7326/M14-2385.
